# Templated deprotonative metalation of polyaryl systems: Facile access to simple, previously inaccessible multi-iodoarenes

**DOI:** 10.1126/sciadv.1700832

**Published:** 2017-06-30

**Authors:** Antonio J. Martínez-Martínez, Stephen Justice, Ben J. Fleming, Alan R. Kennedy, Iain D. H. Oswald, Charles T. O’Hara

**Affiliations:** 1 WestCHEM, Department of Pure and Applied Chemistry, University of Strathclyde, 295 Cathedral Street, Glasgow G1 1XL, UK.; 2 Strathclyde Institute of Pharmacy and Biomedical Sciences, University of Strathclyde, 161 Cathedral Street, Glasgow G4 0RE, UK.

**Keywords:** metalation, arenes, iodoarenes, X-ray diffraction, NMR Spectroscopy, biphenyl

## Abstract

The development of new methodologies to affect non–*ortho*-functionalization of arenes has emerged as a globally important arena for research, which is key to both fundamental studies and applied technologies. A range of simple arene feedstocks (namely, biphenyl, *meta*-terphenyl, *para*-terphenyl, 1,3,5-triphenylbenzene, and biphenylene) is transformed to hitherto unobtainable multi-iodoarenes via an s-block metal sodium magnesiate templated deprotonative approach. These iodoarenes have the potential to be used in a whole host of high-impact transformations, as precursors to key materials in the pharmaceutical, molecular electronic, and nanomaterials industries. To prove the concept, we transformed biphenyl to 3,5-bis(*N*-carbazolyl)-1,1′-biphenyl, a novel isomer of 4,4′-bis(*N*-carbazolyl)-1,1′-biphenyl (CPB), a compound which is currently widely used as a host material for organic light-emitting diodes.

## Teaser

Building blocks for pharmaceutical/electronic materials have been prepared using a templated C–H bond–breaking approach.

## INTRODUCTION

Until 1938, organometallic reagents were considered as exotic rarities, commanding little application in synthetic chemistry (*1*). However, the discoveries by Gilman and Bebb (*2*) and Wittig *et al*. (*3*) that alkyllithium reagents could be used to selectively metalate anisole (and other heterosubstituted arenes) at an *ortho* position opened the doors to the first major utilization of organometallics in synthesis. This discovery was later coined directed *ortho*-metalation (DoM) (*4*), and from this juncture, organometallic reagents were seen as “indispensable tools in chemical synthesis” (*1*). DoM can be used to install new functionalities on arenes, thus allowing the construction of new molecules (*5*–*7*), and relies on converting a relatively inert C–H bond in an organic molecule into a more reactive and versatile C–metal bond by treating it with a metalating reagent (normally an organolithium reagent). The beauty of the DoM reaction is it efficiently and selectively metalates the C–H bond adjacent to a directing group (DG), which promotes reaction due to its dual coordination/inductive roles. Since the pioneering works of Gilman and Bebb (*2*) and Wittig *et al*. (*3*), the DoM concept has been advanced using monometallic (*8*–*12*) and bimetallic (*13*–*19*) systems.

**Fig. 1:**
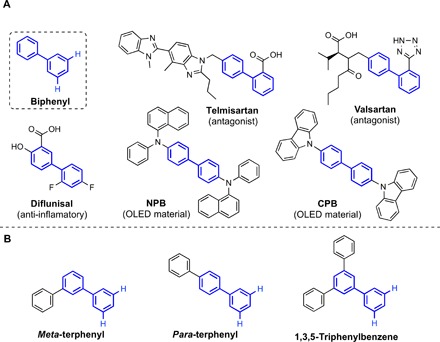
Prevalence of the biphenyl skeleton in pharmaceutical compounds and organic light-emitting diode materials. (**A**) Biphenyl and the structural prevalence of its framework in pharmaceutical agents and host materials for organic light-emitting diodes (OLED). (**B**) Biphenyl-based polyaryl hydrocarbons.

In his influential text, Schlosser stated, “deviations from the *ortho*-rule do exist but are scarce” (*1*). Enabling activation of arene C–H bonds at positions other than *ortho* has become a high interest and impact area of research, with the goal of allowing easy access to key pharmaceuticals, agrochemicals, and novel materials. In the main, new methodologies involve transition metal C–H activation chemistry (*20*–*28*). In 2014, we exploited s-block organometallic chemistry and reported directed *ortho*-*meta′*– and *meta*-*meta′*–dimetalations using a sodium magnesiate reagent [Na_4_Mg_2_(TMP)_6_(^*n*^Bu)_2_] **1** (TMP is 2,2,6,6-tetramethylpiperidide) as an alkyl base (*29*). It functions in a templated manner, allowing double metalations to occur, and has been shown to exhibit regioselective 2,5-double alkyl basicity toward a range of substituted arenes, including amides and carbamates and 3,5-double basicity toward anilines, culminating in the isolation of a group of heterometallic inverse crown molecules (*30*). Here, we use the template metalation strategy to functionalize unactivated multiaryl compounds (non-DoM substrates) that lack acidity due to the absence of functional groups, regioselectively at meta sites. The biphenyl motif (Fig. 1A) is an important building block in the pharmaceutical, agrochemical, and polymer/materials sciences. It is present in telmisartan, valsartan, and oritavancin and is a key component of molecules for organic light-emitting devices, such as *N*,*N′*-di(1-naphthyl)-*N*,*N′*-diphenyl-(1,1′-biphenyl)-4,4′-diamine (NPB) and 4,4′-bis(*N*-carbazolyl)-1,1′-biphenyl (CPB). Biphenyl-based hydrocarbons, such as terphenyls and 1,3,5-triphenylbenzene (tpb) (Fig. 1B), are also cores for molecules used in the devices and for the synthesis of nanomaterials, such as graphene/graphene nanoribbons (*31*). Novel methods to improve the preparation of functionalized multiarenes for these applications are continuously being sought. Reflecting the importance of developing new methods to access novel polyaryl systems, Suzuki *et al.* (*32*) have recently reported the elegant synthesis of a new class of “multitalented” hexaarylbenzenes (*33*) that contain five or six different arene substituents on a single benzene molecule.

## RESULTS AND DISCUSSION

We targeted the templated metalation of simple polyaryl compounds (namely, biphenyl, *para*-terphenyl, *meta*-terphenyl, tpb, and antiaromatic biphenylene) using **1** and subsequently transformed the organometallic intermediates into new versatile organic precursors (*34*). Because of their lack of DGs (hence, the absence of docking site and strong acidifying group), the substrates studied are difficult to metalate using conventional s-block organometallics, unless a Lewis basic functionality activates them. Inklings that this work may be successful stemmed from the fact that we had previously shown that **1** can be used to doubly metalate naphthalene at the 1 and 4 positions regioselectively, which can be subsequently converted to 1,4-diiodonaphthalene (*35*).

**Fig. 2:**
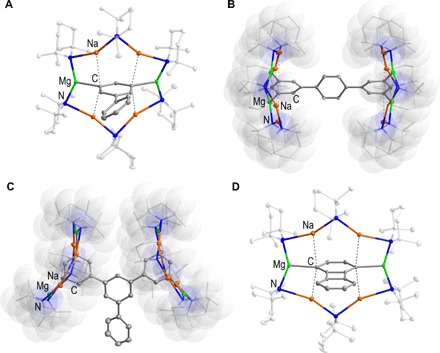
Molecular structures of isolated intermediates. (**A**) **2**. (**B**) **6**. (**C**) **12**. (**D**) **14**. Hydrogen atoms, methylcyclohexane solvent molecules of crystallization, and disordered components of TMP and arene moieties are omitted for clarity. Displacement ellipsoids are displayed at 35% probability with the TMP frameworks pictures as capped sticks with translucent space-filling van der Waals surfaces for a probe of 1.5 Å radius for **6** and **12**.

**Fig. 3:**
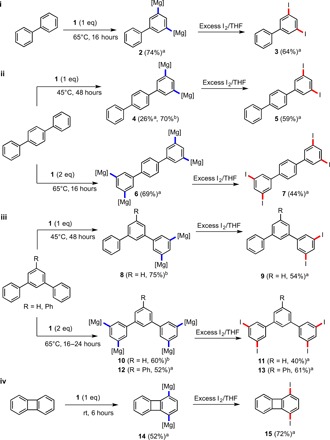
Synthetic studies for the preparation of multi-iodo polyaryl and antiaromatic compounds. ^a^Isolated yield. ^b^Extent of dimetalation determined by NMR spectroscopic studies based on in situ reaction of **1** with the corresponding polyaryl substrate. [Mg] equates to [Na_2_MgTMP_3_]. rt, room temperature.

Substituted biphenyls are often prepared by coupling reactions (*36*). In terms of metalation chemistry, both rings of biphenyl can be singly deprotonated using two equivalents of *n*-butyllithium/TMEDA (*N*,*N*,*N′*,*N′*-tetramethylethylenediamine) (*37*). Synthetically useful yields of monolithiobiphenyls can be obtained using lithium-halogen exchange reactions (*38*, *39*). Introducing a wholly new situation, by treating biphenyl with a methylcyclohexane solution of **1** in a 1:1 molar ratio, it is possible to regioselectively dimagnesiate biphenyl at the 3 and 5 positions of a single phenyl ring, as evidenced by nuclear magnetic resonance (NMR) spectroscopic analysis of the reaction mixture. [Na_4_Mg_2_(TMP)_6_(3,5-biphenyl-di-ide)] **2** was isolated in 70% yield and characterized by x-ray crystallography (Fig. 2A). When two or more equivalents of **1** were used, **2** was again observed, presumably because of the high steric protection afforded to the second phenyl ring, disfavoring further reactivity. When biphenyl is treated with one or two equivalents of the monometallic reagents NaTMP or TMPMg^*n*^Bu, intact unreacted biphenyl was obtained (Supplementary Materials). The key bond parameters of **2** (Supplementary Materials) resemble those of other comparable inverse crown molecules (*29*, *40*). When **2** is reacted with an excess of iodine/tetrahydrofuran (THF) solution, 3,5-diiodobiphenyl **3** can be prepared in 64% isolated yield (Fig. 3, i).

The next arene molecules studied were *meta*- and *para*-terphenyl. Terphenylide anions—prepared via lithium-halogen exchange reactions of 1-halo-2,5-diphenylbenzenes and *n*-butyllithium—are useful ligands in coordination chemistry because they provide a pocket, which protects the metal from forming oligomers despite occupying only one coordination site. Power exploited this special chemistry using a bulky *meta*-terphenyl, 2,6-bis-[(2,6-diisopropyl)phenyl]phenyl to isolate the first quintuple bond between two metals, namely, two Cr(I) cations (*41*). It appears that the parent terphenyls have not thus far been deprotonated using any base. Focusing on *para*-terphenyl, when treated with an equivalent of **1** in methylcyclohexane solution, NMR spectroscopic analysis showed dimetalation of the arene at 3,5 positions of a single phenyl ring, likely producing [Na_4_Mg_2_(TMP)_6_(3,5-*para*-terphenyl-di-ide)] **4** (Supplementary Materials). Because of its high solubility, **4** was isolated in low to moderate yield (26%). An in situ mixture was reacted with iodine/THF solution and successfully converted to 3,5-diiodo-*para*-terphenyl **5** in 59% yield (Fig. 3, ii). As well as characterization by NMR spectroscopy, good quality crystals of **5** were successfully grown from an ethanol solution and were characterized by x-ray crystallography (Supplementary Materials). Increasing the base/substrate ratio (to 2:1) allowed further metalation of the *para*-terphenyl ligand to occur presumably because of the greater distance between the two “outer” phenyl rings (when compared with biphenyl), as evidenced by NMR spectroscopy. Crystals were obtained, which after x-ray analysis showed fourfold metalation of *para*-terphenyl culminating in the isolation of {[Na_4_Mg_2_(TMP)_6_]_2_(3,3″,5,5″-*para*-terphenyl-tetra-ide)} **6** in 69% yield (Fig. 2B). The outer rings of the *para*-terphenyl have each been doubly metalated, and each affected “arene dianion” is hosted by separate inverse crown rings. The key metrics of the inverse crown rings in **6** are essentially identical to those in the biphenyl derivative **2.** Complex **6** represents the first example of a molecule, which contains two inverse crowns within its molecular structure. Previously, it has been shown that metallocenes can also be tetradeprotonated; however, these tetranions are accommodated within a single 16-membered inverse crown ring (*42*, *43*). When isolated **6** is reacted with iodine/THF, NMR spectroscopy reveals that it is transformed to 3,3″,5,5″-tetraiodo-*para*-terphenyl **7** in 51% yield (Fig. 3, ii). When an in situ mixture of **6** is reacted with excess iodine/THF to prepare **7**, a slightly lower yield (44%) was obtained.

The chemistry observed with *meta*-terphenyl (to produce **8** to **11**) essentially mirrors that of **4** to **7**, that is, di- and tetrametalated compounds {[Na_4_Mg_2_(TMP)_6_]_2_(3,5,-*meta*-terphenyl-di-ide)} **8** and {[Na_4_Mg_2_(TMP)_6_]_2_(3,3″,5,5″-*meta*-terphenyl-tetra-ide)} **10**, respectively, were obtained and characterized by NMR spectroscopy, as well as their respective di- and tetraiodo derivatives 3,5-diiodo-*meta*-terphenyl **9** (54%) and 3,3″,5,5″-tetraiodo-*meta*-terphenyl **11** (40%).

Turning to tpb, it has emerged in recent years as an important ligand, formally allowing stabilization of Ca(I) cations (*44*). C–H metalation of tpb is rare, and complexes derived from it generally emerge from the use of a pre–halo-substituted (normally at the central C_6_ ring) reagent. One example of where a genuine C–H metalation is observed is when 1-bromo-2,4,6-triphenylbenzene is reacted with 2 molar equivalents of *n*-butyllithium (*45*). The first equivalent of alkyllithium induces a metal-bromine exchange, and the second metalates an interannular *peri*-C–H bond, formally giving rise to a dianion of triphenylbenzene (*46*). Structurally, tpb seemed an intriguing reagent to metalate. If the reaction was in keeping with the aforementioned results, it could be envisaged that di-, tetra-, or perhaps hexadeprotonation would be possible. In reality, when the arene is reacted with 1 to 3 molar equivalents of **1**, the sole isolated product is the tetradeprotonated variant {[Na_4_Mg_2_(TMP)_6_]_2_{3,3″,5,5″-(1′,3′,5′-triphenylbenzene)-tetra-ide]} **12** (Fig. 2C). A twofold deprotonation of tpb was not detected, perhaps because of the more acidic nature of tpb (in comparison with *meta*-terphenyl) due to the presence of the third phenyl group withdrawing electron density from the other two phenyl rings. When **6** is compared with **12**, there is a marked difference in the positions and orientations of the inverse crown rings (Fig. 2B versus Fig. 2C). In **6**, they adopt a mutual convex orientation residing in a position toward the periphery of the metalated phenyl rings. Likely because of the extra steric demand of the additional phenyl group in tpb, the inverse crowns adopt a mutual concave orientation in **12**, residing in a position more toward the center of the metalated rings. These pronounced structural differences highlight the conformational flexibility of the template, allowing spatially distinct arenes to be accommodated. By reacting **12** with an excess of iodine/THF, it can be smoothly transformed to its tetraiodo relative 3,3″,5,5″-tetraiodo-1′,3′,5′-triphenylbenzene **13** in 61% yield.

Finally, we turned our attention to the prototypical antiaromatic compound biphenylene. Again, reports detailing the direct metalation of biphenylene are very scarce. When reacted with *n*- or *t*-butyllithium and quenched with electrophiles, the major species observed are the 1-substituted biphenylenes (*47*). When biphenylene is reacted with a methylcyclohexane solution of **1**, it was doubly metalated; however, metalation occurred simultaneously at the 1 and 4 positions to yield {[Na_4_Mg_2_(TMP)_6_]_2_(1,4-biphenylene-di-ide)} **14** (Fig. 2D). This metalation pattern is identical to that obtained with naphthalene, presumably because of the similar fused-ring nature of the substrates (thus, each arene ring is initially disubstituted) (*35*). Complex **14** can be converted to 1,4-diiodobiphenylene **15** in high yield (72%) by reaction with iodine/THF.

**Fig. 4:**
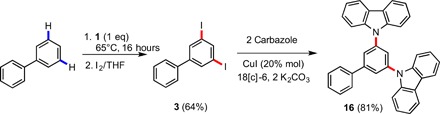
Utilization of 1,3-diiodobiphenyl for Ullmann coupling reaction with carbazole for the preparation of 16.

Organometallic species **2**, **4**, **6**, **8**, **10**, **12**, and **14** are soluble in [D_12_]cyclohexane, allowing multinuclear NMR spectroscopy to be performed. A key finding is that the solid-state structures appear to be maintained in [D_12_]cyclohexane solution (Supplementary Materials). Iodinated organic molecules **3**, **5**, **7**, **9**, **11**, **13**, and **15** are structurally simple; however, surprisingly, this study appears to represent their first synthetic routes, isolation, and full characterization. Access to these molecules has perhaps been hindered because most of polyaryl species are prepared via coupling reactions [for example, Suzuki-Miyaura cross-coupling (*34*)], so the C–I bonds in the potential synthons are themselves susceptible to coupling chemistry, thus it is difficult to prevent undesired overcoupling from occurring. The methodology presented avoids transition metal coupling chemistry, thus the iodine atoms remain intact on the arene rings, allowing further exploitation. Given the importance of iodoarenes as coupling partners in C–C and C–heteroatom bond formation chemistry (*48*, *49*) it is likely that these organic molecules will act as vital building blocks for the synthesis of a whole host of new functional materials. As proof of concept and illustrating the synthetic applicability of our approach, we have prepared a novel isomer of the aforementioned material CPB by converting biphenyl to 3,5-bis(*N*-carbazolyl)-1,1′-biphenyl **16** in high yield (81%; Fig. 4) via copper-catalyzed Ullmann-type coupling of carbazole with **3.**

## MATERIALS AND METHODS

### General methods and chemicals

All reactions and manipulations were performed under a protective atmosphere of dry pure argon gas using standard Schlenk techniques unless otherwise stated. *n*-Hexane was dried by heating it to reflux over sodium benzophenone ketyl and used freshly distilled under nitrogen atmosphere. Methylcyclohexane was distilled over sodium metal under nitrogen and then stored with activated 4 Å molecular sieves under argon before use. Anhydrous *N*,*N*′-dimethylformamide (DMF) was purchased from Sigma-Aldrich and used without further purification. [D_12_]cyclohexane and [D_6_]benzene were degassed and stored over activated 4 Å molecular sieves under argon before use. 2,2,6,6-Tetramethylpiperidine [TMP(H)] was purchased from Merck KGaA and stored over activated molecular sieves (4 Å). ^*n*^BuLi (1.6 M in hexanes) and ^*n*^Bu_2_Mg (1 M in heptane) solutions were purchased from Sigma-Aldrich and titrated before use. Copper iodide, carbazole, potassium carbonate, 18-crown-6, sodium thiosulfate, iodine, anhydrous magnesium sulfate, ammonium chloride, and sodium chloride were purchased from Sigma-Aldrich and used as received. ^*n*^BuNa (*50*), NaTMP (*51*), and TMPMg ^*n*^Bu (*29*, *52*) were prepared according to literature methods and used as solids from a glove box or in situ. Sodium *tert*-butoxide was purchased from Sigma-Aldrich and dried under vacuum at 150°C in a Schlenk tube for 30 min before use. Biphenyl, *meta*-terphenyl (1,3-diphenylbenzene), *para*-terphenyl (1,4-diphenylbenzene), and tpb were purchased from Sigma-Aldrich and used without further purification. Biphenylene was prepared according to the reported method and used as a crystalline solid (*37*). ^*n*^BuNa, NaTMP, TMPMg ^*n*^Bu, and the organometallic products **2**, **4**, **6**, **12**, and **14** were isolated and handled inside an argon-filled glove box, whereas the derivatives **8** and **10** were in situ prepared and used as methylcyclohexane solutions. Organic products **3**, **5**, **7**, **9**, **11**, **13**, **15**, and **16** were isolated without the need of protocols to exclude atmospheric oxygen or moisture. NMR spectra were recorded on a Bruker DPX 400 MHz spectrometer, operating at 400.1 and 100.6 MHz for ^1^H and ^13^C NMR, respectively. The NMR assignments were performed using ^13^C{^1^H}-DEPT135, ^1^H,^1^H-COSY, ^1^H,^13^C-HSQC, and ^1^H,^13^C-HMBC experiments. ^1^H and ^13^C{^1^H} chemical shifts were expressed in parts per million (δ = ppm) and referenced to residual solvent peaks. All coupling constants (*J*) are absolute values (hertz), and the description of signals are br for broad, s for singlet, d for doublet, dd for doublet of doublets, t for triplet, tt for triplet of triplets, q for quartet, and m for multiplet. Elemental analyses (triplicate) were carried out using a PerkinElmer 2400 elemental analyzer. Low-resolution mass spectra for products **3**, **5**, **7**, **9**, **11**, **13**, **15**, and **16** were recorded on either an Agilent 7890A instrument coupled to an Agilent 5975C mass spectrometer (gas chromatography–mass spectrometry, either chemical or electron ionization mode) with helium as a carrier gas at 1 ml/min and using an Restek Rxi-5Sil-MS column (30 m **×** 250 μm **×** 0.25 μm) or an Kratos-Shimadzu Axima CFR MALDI-TOF spectrometer (no matrix) in positive ion mode. Accurate mass spectra were obtained at the University of Swansea in the Engineering and Physical Science Research Council (EPSRC) National Mass Spectrometry Facility, and spectra were recorded using either a Waters GCT Premier benchtop orthogonal acceleration time-of-flight mass spectrometer (electron impact ionization mode) or a Waters Xevo G2-S using the atmospheric solids analysis probe in positive mode. Values are reported as mass/charge ratio (*m*/*z*).

### Preparation of metalating reagent **1**

Solutions of **1** (0.05 and 0.066 M in methylcyclohexane) were prepared in situ according to literature methods (*29*, *35*). Detailed procedures are given for the preparation of **2** to **17** in the Supplementary Materials.

### General synthesis of organometallic reagents **2**, **4**, **6**, **8**, **10**, **12**, and **14**

In an argon-filled Schlenk tube, freshly prepared ^*n*^BuNa (640 mg, 8 mmol) was suspended in methylcyclohexane (24 ml), and TMP(H) (2.04 ml, 12 mmol) was then added via syringe to give NaTMP as a pale yellow suspension, which was stirred for 30 min at ambient temperature. Commercial ^*n*^Bu_2_Mg (4 ml, 1 M solution in *n*-heptane, 4 mmol) was then added via syringe to give a 0.066 M pale yellow solution of **1** in methylcyclohexane. The metalating reagent **1** was stirred for 30 min at ambient temperature before use. Then, the appropriate polyarene was added, and the resulting reaction mixture was heated at the appropriate temperature. Where possible, crystals suitable for x-ray diffraction were grown (see full details in the Supplementary Materials). The NMR spectra of isolated amorphous solids/crystals in [D_12_]cyclohexane solution were obtained.

### General synthesis of organic compounds **3**, **5**, **7**, **9, 11, 13**, and **15**

The corresponding organic substrate (biphenyl, *para*-terphenyl, *meta*-terphenyl, tpb, and biphenylene; 1 mmol each) was treated with an in situ solution of **1** in methylcyclohexane prepared according to the synthetic organometallic protocols described in the Supplementary Materials for **2**, **4**, **6**, **8**, **10**, **12**, and **14**. The 0.05 M solutions of **1** in methylcyclohexane (20 ml) were used for the in situ preparation of **2**, **4**, **8**, and **14**, whereas the 0.066 M solutions of **1** in methylcyclohexane (30 ml) were used for the in situ preparation of **6**, **10**, and **12**. All reactions were stirred at different temperatures and for different times and in situ monitored by NMR spectroscopy in either [D_12_]cyclohexane or [D_6_]benzene until magnesiation of the corresponding aromatic substrate was achieved to give the corresponding organometallic derivatives (table S1). After the in situ metalation step was achieved, the reaction mixture was cooled to −78°C in a dry ice/acetone bath for 30 min and then added (either via cannula or disposable air-tight syringe) to an iodine solution in THF at the same temperature. For the preparation of **3**, **5**, **9**, and **15**, 10 ml of a 1 M solution of iodine in THF was used, whereas for **7**, **11**, and **13**, 20 ml of a 1 M solution of iodine in THF was used. The resulting reaction mixtures were stirred at −78°C for at least 3 hours and then allowed to slowly warm up to ambient temperature for a period of 24 hours. Saturated aqueous NH_4_Cl solution (~10 ml) was then added, followed by saturated aqueous Na_2_S_2_O_3_ solution (~10 ml) until bleaching occurred. The crude mixture was extracted with EtOAc (3 × 20 ml), and the combined organic layers were washed with brine (20 ml) and dried over anhydrous MgSO_4_. The solvent was removed in a rotary evaporator, and the crude product was purified by flash column chromatography (silica gel and *n*-hexane/ethyl acetate) to yield the pure title compound. Alternatively, **7** can be prepared in 51% yield by reacting **6** (1 mmol) with an iodine/THF solution (20 mmol iodine/20 ml THF). The organic derivatives **3**, **5**, **7**, **13**, and **15** can be crystallized from either MeOH, EtOH, or EtOAc. Compounds **7**, **11**, and **13** exhibit low solubility in commonly used organic solvents, which hinders their isolation and purification in the scale used in their preparations.

### Synthesis of **16**

In an argon-filled Schlenk tube, 3,5-diiodo-1,1′-biphenyl (150 mg, 0.37 mmol) was mixed with carbazole (140 mg, 0.83 mmol), potassium carbonate (114 mg, 0.83 mmol), copper(I) iodide (32 mg, 0.17 mmol), and 18-crown-6 (30 mg, 0.1 mmol). Then, anhydrous DMF was added (5 ml), and the reaction mixture was refluxed for 24 hours under argon atmosphere. After cooling down to ambient temperature, the reaction mixture was filtered through a short pad of Celite, and the solvent was removed under vacuum. The crude product was purified by flash column chromatography (silica gel and *n*-hexane/ethyl acetate) to form **16** as a pale yellow solid (0.145 g, 81%). Compound **16** can be crystallized from ethyl acetate by slow evaporation to give colorless block-like crystals suitable for an x-ray diffraction study (see the Supplementary Materials for NMR spectroscopic and mass spectral data).

## Supplementary Material

http://advances.sciencemag.org/cgi/content/full/3/6/e1700832/DC1

## SUPPLEMENTARY MATERIALS

Supplementary material for this article is available at http://advances.sciencemag.org/cgi/content/full/3/6/e1700832/DC1 Detailed experimental procedures Materials and methods Synthetic procedures X-ray crystallography Supplementary text NMR spectra fig. S1. Sections of the ^1^H NMR (400.1 MHz; [D_12_]cyclohexane, 300 K) spectra showing the nonaromatic resonances for biphenylene (top; black), reaction mixture containing two conformers of **14** (middle; blue), and isolated [Na_4_Mg_2_(TMP)_6_(1,4-biphenylene-di-ide)] **14** (bottom; major conformer). fig. S2. ^1^H,^1^H-COSY NMR (400.1 MHz; [D_12_]cyclohexane, 300 K) spectrum showing the nonaromatic resonances for the two conformers of **14**. fig. S3. Sections of experimental and simulated ^1^H NMR spectra of **14**. fig. S4. Sections of experimental and simulated ^1^H NMR spectra of **15**. fig. S5. Molecular structure of **2** showing the contents of the asymmetric unit cell. fig. S6. Molecular structure of **3** and its extended packing. fig. S7. Molecular structure of **5** and its extended packing. fig. S8. Molecular structure of **6** showing atomic connectivity. fig. S9. Molecular structure of **12** showing the contents of the asymmetric unit cell. fig. S10. Molecular structure of **13** and its extended packing. fig. S11. Molecular structure of **14** showing the contents of the asymmetric unit cell. fig. S12. Molecular structure of **15** and its extended packing. fig. S13. Molecular structure of **16** showing the contents of the asymmetric unit cell. fig. S14. ^1^H NMR study (400.1 MHz; [D_12_]cyclohexane, 300 K) of biphenyl (top; red) and a control reaction of biphenyl and NaTMP in a 1:2 M ratio in methylcyclohexane after 24 hours at 65°C (bottom; blue). fig. S15. ^1^H NMR study (400.1 MHz; [D_12_]cyclohexane, 300 K) of biphenyl (top; red) and a control reaction of biphenyl and ^*n*^BuMgTMP in a 1:2 M ratio in methylcyclohexane after 16 hours at 65°C (bottom; blue). fig. S16. Representative example of 3,5-dimetalation of biphenyl to give **2**. fig. S17. ^1^H NMR (400.1 MHz; [D_12_]cyclohexane, 300 K) spectrum of **2**. fig. S18. ^13^C{^1^H} NMR (100.6 MHz; [D_12_]cyclohexane, 300 K) spectrum of **2**. fig. S19. Sections of the ^1^H,^1^H-COSY NMR (400.1 MHz; [D_12_]cyclohexane, 300 K) spectrum of **2**. fig. S20. Sections of the phase-sensitive ^1^H,^13^C-HSQC NMR (400.1 MHz; [D_12_]cyclohexane, 300 K) spectrum of **2**. fig. S21. Sections of the ^1^H,^13^C-HMBC NMR (400.1 MHz; [D_12_]cyclohexane, 300 K) spectrum of **2**. fig. S22. Sections of the ^1^H NMR (400.1 MHz; [D_12_]cyclohexane, 300 K) spectra of biphenyl (top; red) and **2** (bottom; blue) showing the aromatic resonances. fig. S23. Sections of the ^13^C{^1^H} NMR (100.6 MHz; [D_12_]cyclohexane, 300 K) spectra of biphenyl (top; red) and **2** (bottom; blue) showing the aromatic resonances. fig. S24. ^1^H NMR (400.1 MHz; CDCl_3_, 300 K) spectrum of **3**. fig. S25. ^13^C{^1^H} NMR (100.6 MHz; CDCl_3_, 300 K) spectrum of **3**. fig. S26. ^1^H,^1^H-COSY NMR (400.1 MHz; CDCl_3_, 300 K) spectrum of **3**. fig. S27. ^1^H,^13^C-HSQC NMR (400.1 MHz; CDCl_3_, 300 K) spectrum of **3**. fig. S28. ^1^H,^13^C-HMBC NMR (400.1 MHz; CDCl_3_, 300 K) spectrum of **3**. fig. S29. ^1^H NMR (400.1 MHz; [D_12_]cyclohexane, 300 K) spectrum of an in situ sample of **4**. fig. S30. ^1^H NMR (400.1 MHz; CDCl_3_, 300 K) spectrum of **5**. fig. S31. ^13^C{^1^H} NMR (100.6 MHz; CDCl_3_, 300 K) spectrum of **5**. fig. S32. ^1^H,^1^H-COSY NMR (400.1 MHz; CDCl_3_, 300 K) spectrum of **5**. fig. S33. ^1^H,^13^C-HSQC NMR (400.1 MHz; CDCl_3_, 300 K) spectrum of **5**. fig. S34. Sections of the ^1^H,^13^C-HMBC NMR (400.1 MHz; CDCl_3_, 300 K) spectrum of **5**. fig. S35. ^1^H NMR (400.1 MHz; [D_12_]cyclohexane/C_7_H_14_, 300 K) spectrum of [Na_8_Mg_4_(TMP)_12_(3,3′,5,5′-*para*-terphenyl-tetra-ide)] **6**. fig. S36. Section of the ^1^H,^1^H-COSY NMR (400.1 MHz; [D_12_]cyclohexane/C_7_H_14_, 300 K) spectrum of [Na_8_Mg_4_(TMP)_12_(3,3′,5,5′-*para*-terphenyl-tetra-ide)] **6** showing the cross peaks for the aromatic resonances. fig. S37. Sections of the ^1^H NMR (400.1 MHz; [D_12_]cyclohexane, 300 K) spectra of *para*-terphenyl (top; green), **4** (middle; red), and [Na_8_Mg_4_(TMP)_12_(3,3′,5,5′-*para*-terphenyl-tetra-ide)] **6** (bottom; blue). fig. S38. ^1^H NMR (400.1 MHz; CDCl_3_, 300 K) spectrum of **7**. fig. S39. ^13^C NMR (100.6 MHz; CDCl_3_, 300 K) spectrum of **7**. fig. S40. ^1^H NMR (400.1 MHz; [D_12_]cyclohexane, 300 K) spectrum of [Na_4_Mg_2_(TMP)_6_(3,5-*meta*-terphenyl-di-ide)] **8**. fig. S41. ^13^C{^1^H} NMR (100.6 MHz; [D_12_]cyclohexane, 300 K) spectrum of [Na_4_Mg_2_(TMP)_6_(3,5-*meta*-terphenyl-di-ide)] **8**. fig. S42. ^1^H NMR (400.1 MHz; 300 K, CDCl_3_) spectrum of **9**. fig. S43. ^13^C{^1^H} NMR (100.6 MHz; 300 K, CDCl_3_) spectrum of **9**. fig. S44. ^1^H,^1^H-COSY NMR (400.1 MHz; 300 K, CDCl_3_) spectrum of 3,5-diiodo-*meta*-terphenyl **9**. fig. S45. ^1^H,^13^C-HSQC NMR (400.1 MHz; 300 K, CDCl_3_) spectrum of **9**. fig. S46. ^1^H,^13^C-HMBC NMR (400.1 MHz; 300 K, CDCl_3_) spectrum of **9**. fig. S47. ^1^H NMR (400.1 MHz; [D_12_]cyclohexane, 300 K) spectrum of [Na_8_Mg_4_TMP_12_(3,3′,5,3′-*meta*-terphenyl-tetra-ide)] **10**. fig. S48. Sections of the ^1^H NMR (400.1 MHz; [D_12_]cyclohexane, 300 K) spectra of *meta*-terphenyl (top; green), [Na_4_Mg_2_(TMP)_6_(3,5-*meta*-terphenyl-di-ide)] **8** (middle; red), and [Na_8_Mg_4_TMP_12_(3,3′,5,3′-*meta*-terphenyl-tetra-ide)] **10** (bottom; blue) showing the aromatic resonances. fig. S49. ^1^H NMR (400.1 MHz; CDCl_3_, 300 K) spectrum of **11**. fig. S50. ^13^C{^1^H} NMR (100.6 MHz; CDCl_3_, 300 K) spectrum of **11**. fig. S51. ^1^H,^1^H-COSY NMR (400.1 MHz; CDCl_3_, 300 K) spectrum of **9**. fig. S52. ^1^H,^13^C-HSQC NMR (400.1 MHz; CDCl_3_, 300 K) spectrum of **9**. fig. S53. ^1^H,^13^C-HMBC NMR (400.1 MHz; CDCl_3_, 300 K) spectrum of **9**. fig. S54. ^1^H NMR (400.1 MHz; [D_6_]benzene, 300 K) spectrum of {Na_8_Mg_4_[TMP]_12_[3,3″,5,5″-(1′,3′,5′-triphenylbenzene-tetra-ide)]} **12**. fig. S55. Section of the ^1^H,^1^H-COSY NMR (400.1 MHz; [D_6_]benzene, 300 K) spectrum of **12** showing the cross peaks for the aromatic resonances. fig. S56. ^1^H NMR (400.1 MHz; [D_6_]benzene, 300 K) spectra of tpb (top; red) and **12** (bottom; blue). fig. S57. ^1^H NMR (400.1 MHz; CDCl_3_, 300 K) spectrum of 3,3″,5,5″-tetraiodo-5′-phenyl-benzene **13**. fig. S58. ^13^C{^1^H} NMR (100.6 MHz; CDCl_3_, 300 K) spectrum of **13**. fig. S59. ^1^H,^1^H-COSY NMR (400.1 MHz; CDCl_3_, 300 K) spectrum of **13**. fig. S60. ^1^H,^13^C-HSQC NMR (400.1 MHz; CDCl_3_, 300 K) spectrum of **13**. fig. S61. ^1^H,^13^C-HMBC NMR (400.1 MHz; CDCl_3_, 300 K) spectrum sections of **13**. fig. S62. ^1^H NMR (400.1 MHz; [D_12_]cyclohexane, 300 K) spectrum of **14**. fig. S63. ^13^C{^1^H} NMR (100.6 MHz; [D_12_]cyclohexane, 300 K) spectrum of **14**. fig. S64. Sections of the ^1^H,^1^H-COSY NMR (400.1 MHz; [D_12_]cyclohexane, 300 K) spectrum of **14**. fig. S65. Sections of the phase-sensitive ^1^H,^13^C-HSQC NMR (400.1 MHz; [D_12_]cyclohexane, 300 K) spectrum of **14.** fig. S66. Sections of the ^1^H,^13^C-HMBC NMR (400.1 MHz; [D_12_]cyclohexane, 300 K) spectrum of **14**. fig. S67. ^13^C{^1^H} NMR (100.6 MHz; [D_12_]cyclohexane, 300 K) section of the spectra of biphenylene (top; red) and isolated **14** (bottom; blue, major conformer). fig. S68. ^1^H NMR (400.1 MHz; CDCl_3_, 300 K) spectrum of **15**. fig. S69. ^13^C{^1^H} NMR (100.6 MHz; CDCl_3_, 300 K) spectrum of **15**. fig. S70. ^1^H,^1^H-COSY NMR (400.1 MHz; CDCl_3_, 300 K) spectrum of **15**. fig. S71. ^1^H,^13^C-HSQC NMR (400.1 MHz; CDCl_3_, 300 K) spectrum of **15**. fig. S72. ^1^H,^13^C-HMBC NMR (400.1 MHz; CDCl_3_, 300 K) spectrum of **15**. fig. S73. ^1^H NMR (400.1 MHz; CDCl_3_, 300 K) spectra of biphenylene (top; red) and **15** (bottom; blue). fig. S74. ^13^C{^1^H} NMR (100.6 MHz; CDCl_3_, 300 K) spectra of biphenylene (top; red) and **15** (bottom; blue). fig. S75. ^1^H NMR (400.1 MHz; CDCl_3_, 300 K) spectrum of **16**. fig. S76. ^13^C{^1^H} NMR (100.6 MHz; CDCl_3_, 300 K) spectrum of **16**. fig. S77. ^1^H,^1^H-COSY NMR (400.1 MHz; CDCl_3_, 300 K) spectrum of **16**. fig. S78. ^1^H,^13^C-HSQC NMR (400.1 MHz; CDCl_3_, 300 K) spectrum of **16**. fig. S79. ^1^H,^13^C-HMBC NMR (400.1 MHz; CDCl_3_, 300 K) spectrum of **16**. table S1. Metalation conditions and scope. References (*53*–*62*)
