# Supplementary material for: Templated deprotonative metalation of polyaryl systems: Facile access to simple, previously inaccessible multi-iodoarenes
Source: Sci Adv. 2017 Jun 30;3(6):e1700832. doi: 10.1126/sciadv.1700832 (PMC5493416; doi:10.1126/sciadv.1700832)
Supplement: http://advances.sciencemag.org/cgi/content/full/3/6/e1700832/DC1 [file supp_3_6_e1700832__index.html]

Science Advances | Science Advances

## Supplementary Materials

**This PDF file includes:**

- Detailed experimental procedures
- Materials and methods
- Synthetic procedures
- X-ray crystallography
- Supplementary text
- NMR spectra
- fig. S1. Sections of the 1H NMR (400.1 MHz; D12cyclohexane, 300 K) spectra showing the nonaromatic resonances for biphenylene (top; black), reaction mixture containing two conformers of **14** (middle; blue), and isolated Na4Mg2(TMP)6(1,4-biphenylene-di-ide) **14** (bottom; major conformer).
- fig. S2. 1H,1H-COSY NMR (400.1 MHz; D12cyclohexane, 300 K) spectrum showing the nonaromatic resonances for the two conformers of **14**.
- fig. S3. Sections of experimental and simulated 1H NMR spectra of **14**.
- fig. S4. Sections of experimental and simulated 1H NMR spectra of **15**.
- fig. S5. Molecular structure of **2** showing the contents of the asymmetric unit cell.
- fig. S6. Molecular structure of **3** and its extended packing.
- fig. S7. Molecular structure of **5** and its extended packing.
- fig. S8. Molecular structure of **6** showing atomic connectivity.
- fig. S9. Molecular structure of **12** showing the contents of the asymmetric unit cell.
- fig. S10. Molecular structure of **13** and its extended packing.
- fig. S11. Molecular structure of **14** showing the contents of the asymmetric unit cell.
- fig. S12. Molecular structure of **15** and its extended packing.
- fig. S13. Molecular structure of **16** showing the contents of the asymmetric unit cell.
- fig. S14. 1H NMR study (400.1 MHz; D12cyclohexane, 300 K) of biphenyl (top; red) and a control reaction of biphenyl and NaTMP in a 1:2 M ratio in methylcyclohexane after 24 hours at 65°C (bottom; blue).
- fig. S15. 1H NMR study (400.1 MHz; D12cyclohexane, 300 K) of biphenyl (top; red) and a control reaction of biphenyl and nBuMgTMP in a 1:2 M ratio in methylcyclohexane after 16 hours at 65°C (bottom; blue).
- fig. S16. Representative example of 3,5-dimetalation of biphenyl to give **2**.
- fig. S17. 1H NMR (400.1 MHz; D12cyclohexane, 300 K) spectrum of **2**.
- fig. S18. 13C{1H} NMR (100.6 MHz; D12cyclohexane, 300 K) spectrum of **2**.
- fig. S19. Sections of the 1H,1H-COSY NMR (400.1 MHz; D12cyclohexane, 300 K) spectrum of **2**.
- fig. S20. Sections of the phase-sensitive 1H,13C-HSQC NMR (400.1 MHz; D12cyclohexane, 300 K) spectrum of **2**.
- fig. S21. Sections of the 1H,13C-HMBC NMR (400.1 MHz; D12cyclohexane, 300 K) spectrum of **2**.
- fig. S22. Sections of the 1H NMR (400.1 MHz; D12cyclohexane, 300 K) spectra of biphenyl (top; red) and 2 (bottom; blue) showing the aromatic resonances.
- fig. S23. Sections of the 13C{1H} NMR (100.6 MHz; D12cyclohexane, 300 K) spectra of biphenyl (top; red) and **2** (bottom; blue) showing the aromatic resonances.
- fig. S24. 1H NMR (400.1 MHz; CDCl3, 300 K) spectrum of **3**.
- fig. S25. 13C{1H} NMR (100.6 MHz; CDCl3, 300 K) spectrum of **3**.
- fig. S26. 1H,1H-COSY NMR (400.1 MHz; CDCl3, 300 K) spectrum of **3**.
- fig. S27. 1H,13C-HSQC NMR (400.1 MHz; CDCl3, 300 K) spectrum of **3**.
- fig. S28. 1H,13C-HMBC NMR (400.1 MHz; CDCl3, 300 K) spectrum of **3**.
- fig. S29. 1H NMR (400.1 MHz; D12cyclohexane, 300 K) spectrum of an in situ sample of 4.
- fig. S30. 1H NMR (400.1 MHz; CDCl3, 300 K) spectrum of **5**.
- fig. S31. 13C{1H} NMR (100.6 MHz; CDCl3, 300 K) spectrum of **5**.
- fig. S32. 1H,1H-COSY NMR (400.1 MHz; CDCl3, 300 K) spectrum of **5**.
- fig. S33. 1H,13C-HSQC NMR (400.1 MHz; CDCl3, 300 K) spectrum of **5**.
- fig. S34. Sections of the 1H,13C-HMBC NMR (400.1 MHz; CDCl3, 300 K) spectrum of **5**.
- fig. S35. 1H NMR (400.1 MHz; D12cyclohexane/C7H14, 300 K) spectrum of Na8Mg4(TMP)12(3,3′,5,5′-*para*-terphenyl-tetra-ide) **6**.
- fig. S36. Section of the 1H,1H-COSY NMR (400.1 MHz; D12cyclohexane/C7H14, 300 K) spectrum of Na8Mg4(TMP)12(3,3′,5,5′-*para*-terphenyl-tetra-ide) **6** showing the cross peaks for the aromatic resonances.
- fig. S37. Sections of the 1H NMR (400.1 MHz; D12cyclohexane, 300 K) spectra of *para*-terphenyl (top; green), **4** (middle; red), and Na8Mg4(TMP)12(3,3′,5,5′-*para*-terphenyl-tetra-ide) **6** (bottom; blue).
- fig. S38. 1H NMR (400.1 MHz; CDCl3, 300 K) spectrum of **7**.
- fig. S39. 13C NMR (100.6 MHz; CDCl3, 300 K) spectrum of **7**.
- fig. S40. 1H NMR (400.1 MHz; D12cyclohexane, 300 K) spectrum of Na4Mg2(TMP)6(3,5-*meta*-terphenyl-di-ide) **8**.
- fig. S41. 13C{1H} NMR (100.6 MHz; D12cyclohexane, 300 K) spectrum of Na4Mg2(TMP)6(3,5-*meta*-terphenyl-di-ide) **8**.
- fig. S42. 1H NMR (400.1 MHz; 300 K, CDCl3) spectrum of **9**.
- fig. S43. 13C{1H} NMR (100.6 MHz; 300 K, CDCl3) spectrum of **9**.
- fig. S44. 1H,1H-COSY NMR (400.1 MHz; 300 K, CDCl3) spectrum of 3,5-diiodo-*meta*-terphenyl **9**.
- fig. S45. 1H,13C-HSQC NMR (400.1 MHz; 300 K, CDCl3) spectrum of **9**.
- fig. S46. 1H,13C-HMBC NMR (400.1 MHz; 300 K, CDCl3) spectrum of **9**.
- fig. S47. 1H NMR (400.1 MHz; D12cyclohexane, 300 K) spectrum of Na8Mg4TMP12(3,3′,5,3′-*meta*-terphenyl-tetra-ide) 10.
- fig. S48. Sections of the 1H NMR (400.1 MHz; D12cyclohexane, 300 K) spectra of *meta*-terphenyl (top; green), Na4Mg2(TMP)6(3,5-*meta*-terphenyl-di-ide) **8** (middle; red), and Na8Mg4TMP12(3,3′,5,3′-*meta*-terphenyl-tetra-ide) **10** (bottom; blue) showing the aromatic resonances.
- fig. S49. 1H NMR (400.1 MHz; CDCl3, 300 K) spectrum of **11**.
- fig. S50. 13C{1H} NMR (100.6 MHz; CDCl3, 300 K) spectrum of **11**.
- fig. S51. 1H,1H-COSY NMR (400.1 MHz; CDCl3, 300 K) spectrum of **9**.
- fig. S52. 1H,13C-HSQC NMR (400.1 MHz; CDCl3, 300 K) spectrum of **9**.
- fig. S53. 1H,13C-HMBC NMR (400.1 MHz; CDCl3, 300 K) spectrum of **9**.
- fig. S54. 1H NMR (400.1 MHz; D6benzene, 300 K) spectrum of {Na8Mg4TMP123,3″,5,5″-(1′,3′,5′-triphenylbenzene-tetra-ide)} **12**.
- fig. S55. Section of the 1H,1H-COSY NMR (400.1 MHz; D6benzene, 300 K) spectrum of **12** showing the cross peaks for the aromatic resonances.
- fig. S56. 1H NMR (400.1 MHz; D6benzene, 300 K) spectra of tpb (top; red) and **12** (bottom; blue).
- fig. S57. 1H NMR (400.1 MHz; CDCl3, 300 K) spectrum of **3**,3″,5,5″-tetraiodo-5′-phenyl-benzene **13**.
- fig. S58. 13C{1H} NMR (100.6 MHz; CDCl3, 300 K) spectrum of **13**.
- fig. S59. 1H,1H-COSY NMR (400.1 MHz; CDCl3, 300 K) spectrum of **13**.
- fig. S60. 1H,13C-HSQC NMR (400.1 MHz; CDCl3, 300 K) spectrum of **13**.
- fig. S61. 1H,13C-HMBC NMR (400.1 MHz; CDCl3, 300 K) spectrum sections of **13**.
- fig. S62. 1H NMR (400.1 MHz; D12cyclohexane, 300 K) spectrum of **14**.
- fig. S63. 13C{1H} NMR (100.6 MHz; D12cyclohexane, 300 K) spectrum of **14**.
- fig. S64. Sections of the 1H,1H-COSY NMR (400.1 MHz; D12cyclohexane, 300 K) spectrum of **14**.
- fig. S65. Sections of the phase-sensitive 1H,13C-HSQC NMR (400.1 MHz; D12cyclohexane, 300 K) spectrum of **14**.
- fig. S66. Sections of the 1H,13C-HMBC NMR (400.1 MHz; D12cyclohexane, 300 K) spectrum of **14**.
- fig. S67. 13C{1H} NMR (100.6 MHz; D12cyclohexane, 300 K) section of the spectra of biphenylene (top; red) and isolated 14 (bottom; blue, major conformer).
- fig. S68. 1H NMR (400.1 MHz; CDCl3, 300 K) spectrum of **15**.
- fig. S69. 13C{1H} NMR (100.6 MHz; CDCl3, 300 K) spectrum of **15**.
- fig. S70. 1H,1H-COSY NMR (400.1 MHz; CDCl3, 300 K) spectrum of **15**.
- fig. S71. 1H,13C-HSQC NMR (400.1 MHz; CDCl3, 300 K) spectrum of **15**.
- fig. S72. 1H,13C-HMBC NMR (400.1 MHz; CDCl3, 300 K) spectrum of **15**.
- fig. S73. 1H NMR (400.1 MHz; CDCl3, 300 K) spectra of biphenylene (top; red) and 15 (bottom; blue).
- fig. S74. 13C{1H} NMR (100.6 MHz; CDCl3, 300 K) spectra of biphenylene (top; red) and 15 (bottom; blue).
- fig. S75. 1H NMR (400.1 MHz; CDCl3, 300 K) spectrum of **16**.
- fig. S76. 13C{1H} NMR (100.6 MHz; CDCl3, 300 K) spectrum of **16**.
- fig. S77. 1H,1H-COSY NMR (400.1 MHz; CDCl3, 300 K) spectrum of **16**.
- fig. S78. 1H,13C-HSQC NMR (400.1 MHz; CDCl3, 300 K) spectrum of **16**.
- fig. S79. 1H,13C-HMBC NMR (400.1 MHz; CDCl3, 300 K) spectrum of **16**.
- table S1. Metalation conditions and scope.
- References (*53–55, 57–62*)

Download PDF

**Files in this Data Supplement:**

- Adobe PDF - 1700832\_SM.pdf
