# Supplementary material for: Templated deprotonative metalation of polyaryl systems: Facile access to simple, previously inaccessible multi-iodoarenes
Source: Sci Adv. 2017 Jun 30;3(6):e1700832. doi: 10.1126/sciadv.1700832 (PMC5493416; doi:10.1126/sciadv.1700832)
Supplement: http://advances.sciencemag.org/cgi/content/full/3/6/e1700832/DC1 [file 1700832_SM.pdf]

Science Advances

AAAS

advances.sciencemag.org/cgi/content/full/3/6/e1700832/DC1

Supplementary Materials for

Templated deprotonative metalation of polyaryl systems: Facile access to simple, previously inaccessible multi-iodoarenes

Antonio J. Martínez-Martínez, Stephen Justice, Ben J. Fleming, Alan R. Kennedy, Iain D. H. Oswald, Charles T. O'Hara

Published 30 June 2017, *Sci. Adv.* **3**, e1700832 (2017)
DOI: 10.1126/sciadv.1700832

This PDF file includes:

- Detailed experimental procedures
- Materials and methods
- Synthetic procedures
- X-ray crystallography
- Supplementary text
- NMR spectra
- fig. S1. Sections of the ^{1}H NMR (400.1 MHz; [D_{12}]cyclohexane, 300 K) spectra showing the nonaromatic resonances for biphenylene (top; black), reaction mixture containing two conformers of **14** (middle; blue), and isolated [$Na_4Mg_2(TMP)_6$(1,4-biphenylene-di-ide)] **14** (bottom; major conformer).
- fig. S2. ^{1}H,^{1}H-COSY NMR (400.1 MHz; [D_{12}]cyclohexane, 300 K) spectrum showing the nonaromatic resonances for the two conformers of **14**.
- fig. S3. Sections of experimental and simulated ^{1}H NMR spectra of **14**.
- fig. S4. Sections of experimental and simulated ^{1}H NMR spectra of **15**.
- fig. S5. Molecular structure of **2** showing the contents of the asymmetric unit cell.
- fig. S6. Molecular structure of **3** and its extended packing.
- fig. S7. Molecular structure of **5** and its extended packing.
- fig. S8. Molecular structure of **6** showing atomic connectivity.
- fig. S9. Molecular structure of **12** showing the contents of the asymmetric unit cell.
- fig. S10. Molecular structure of **13** and its extended packing.
- fig. S11. Molecular structure of **14** showing the contents of the asymmetric unit cell.
- fig. S12. Molecular structure of **15** and its extended packing.

- fig. S13. Molecular structure of **16** showing the contents of the asymmetric unit cell.
- fig. S14. ^{1}H NMR study (400.1 MHz; [D_{12}]cyclohexane, 300 K) of biphenyl (top; red) and a control reaction of biphenyl and NaTMP in a 1:2 M ratio in methylcyclohexane after 24 hours at 65°C (bottom; blue).
- fig. S15. ^{1}H NMR study (400.1 MHz; [D_{12}]cyclohexane, 300 K) of biphenyl (top; red) and a control reaction of biphenyl and ^{n}BuMgTMP in a 1:2 M ratio in methylcyclohexane after 16 hours at 65°C (bottom; blue).
- fig. S16. Representative example of 3,5-dimetalation of biphenyl to give **2**.
- fig. S17. ^{1}H NMR (400.1 MHz; [D_{12}]cyclohexane, 300 K) spectrum of **2**.
- fig. S18. $^{13}C\{^{1}H\}$ NMR (100.6 MHz; [D_{12}]cyclohexane, 300 K) spectrum of **2**.
- fig. S19. Sections of the $^{1}H,^{1}H$-COSY NMR (400.1 MHz; [D_{12}]cyclohexane, 300 K) spectrum of **2**.
- fig. S20. Sections of the phase-sensitive $^{1}H,^{13}C$-HSQC NMR (400.1 MHz; [D_{12}]cyclohexane, 300 K) spectrum of **2**.
- fig. S21. Sections of the $^{1}H,^{13}C$-HMBC NMR (400.1 MHz; [D_{12}]cyclohexane, 300 K) spectrum of **2**.
- fig. S22. Sections of the ^{1}H NMR (400.1 MHz; [D_{12}]cyclohexane, 300 K) spectra of biphenyl (top; red) and **2** (bottom; blue) showing the aromatic resonances.
- fig. S23. Sections of the $^{13}C\{^{1}H\}$ NMR (100.6 MHz; [D_{12}]cyclohexane, 300 K) spectra of biphenyl (top; red) and **2** (bottom; blue) showing the aromatic resonances.
- fig. S24. ^{1}H NMR (400.1 MHz; $CDCl_3$, 300 K) spectrum of **3**.
- fig. S25. $^{13}C\{^{1}H\}$ NMR (100.6 MHz; $CDCl_3$, 300 K) spectrum of **3**.
- fig. S26. $^{1}H,^{1}H$-COSY NMR (400.1 MHz; $CDCl_3$, 300 K) spectrum of **3**.
- fig. S27. $^{1}H,^{13}C$-HSQC NMR (400.1 MHz; $CDCl_3$, 300 K) spectrum of **3**.
- fig. S28. $^{1}H,^{13}C$-HMBC NMR (400.1 MHz; $CDCl_3$, 300 K) spectrum of **3**.
- fig. S29. ^{1}H NMR (400.1 MHz; [D_{12}]cyclohexane, 300 K) spectrum of an in situ sample of **4**.
- fig. S30. ^{1}H NMR (400.1 MHz; $CDCl_3$, 300 K) spectrum of **5**.
- fig. S31. $^{13}C\{^{1}H\}$ NMR (100.6 MHz; $CDCl_3$, 300 K) spectrum of **5**.
- fig. S32. $^{1}H,^{1}H$-COSY NMR (400.1 MHz; $CDCl_3$, 300 K) spectrum of **5**.
- fig. S33. $^{1}H,^{13}C$-HSQC NMR (400.1 MHz; $CDCl_3$, 300 K) spectrum of **5**.
- fig. S34. Sections of the $^{1}H,^{13}C$-HMBC NMR (400.1 MHz; $CDCl_3$, 300 K) spectrum of **5**.
- fig. S35. ^{1}H NMR (400.1 MHz; [D_{12}]cyclohexane/C_7H_{14}, 300 K) spectrum of [$Na_8Mg_4(TMP)_{12}$(3,3′,5,5′-*para*-terphenyl-tetra-ide)] **6**.
- fig. S36. Section of the $^{1}H,^{1}H$-COSY NMR (400.1 MHz; [D_{12}]cyclohexane/C_7H_{14}, 300 K) spectrum of [$Na_8Mg_4(TMP)_{12}$(3,3′,5,5′-*para*-terphenyl-tetra-ide)] **6** showing the cross peaks for the aromatic resonances.
- fig. S37. Sections of the ^{1}H NMR (400.1 MHz; [D_{12}]cyclohexane, 300 K) spectra of *para*-terphenyl (top; green), **4** (middle; red), and [$Na_8Mg_4(TMP)_{12}$(3,3′,5,5′-*para*-terphenyl-tetra-ide)] **6** (bottom; blue).
- fig. S38. ^{1}H NMR (400.1 MHz; $CDCl_3$, 300 K) spectrum of **7**.
- fig. S39. ^{13}C NMR (100.6 MHz; $CDCl_3$, 300 K) spectrum of **7**.

- fig. S40. ^{1}H NMR (400.1 MHz; [D_{12}]cyclohexane, 300 K) spectrum of [$Na_4Mg_2(TMP)_6$(3,5-*meta*-terphenyl-di-ide)] **8**.
- fig. S41. ^{13}C{^{1}H} NMR (100.6 MHz; [D_{12}]cyclohexane, 300 K) spectrum of [$Na_4Mg_2(TMP)_6$(3,5-*meta*-terphenyl-di-ide)] **8**.
- fig. S42. ^{1}H NMR (400.1 MHz; 300 K, $CDCl_3$) spectrum of **9**.
- fig. S43. ^{13}C{^{1}H} NMR (100.6 MHz; 300 K, $CDCl_3$) spectrum of **9**.
- fig. S44. ^{1}H,^{1}H-COSY NMR (400.1 MHz; 300 K, $CDCl_3$) spectrum of 3,5-diiodo-*meta*-terphenyl **9**.
- fig. S45. ^{1}H,^{13}C-HSQC NMR (400.1 MHz; 300 K, $CDCl_3$) spectrum of **9**.
- fig. S46. ^{1}H,^{13}C-HMBC NMR (400.1 MHz; 300 K, $CDCl_3$) spectrum of **9**.
- fig. S47. ^{1}H NMR (400.1 MHz; [D_{12}]cyclohexane, 300 K) spectrum of [$Na_8Mg_4TMP_{12}$(3,3′,5,3′-*meta*-terphenyl-tetra-ide)] **10**.
- fig. S48. Sections of the ^{1}H NMR (400.1 MHz; [D_{12}]cyclohexane, 300 K) spectra of *meta*-terphenyl (top; green), [$Na_4Mg_2(TMP)_6$(3,5-*meta*-terphenyl-di-ide)] **8** (middle; red), and [$Na_8Mg_4TMP_{12}$(3,3′,5,3′-*meta*-terphenyl-tetra-ide)] **10** (bottom; blue) showing the aromatic resonances.
- fig. S49. ^{1}H NMR (400.1 MHz; $CDCl_3$, 300 K) spectrum of **11**.
- fig. S50. ^{13}C{^{1}H} NMR (100.6 MHz; $CDCl_3$, 300 K) spectrum of **11**.
- fig. S51. ^{1}H,^{1}H-COSY NMR (400.1 MHz; $CDCl_3$, 300 K) spectrum of **9**.
- fig. S52. ^{1}H,^{13}C-HSQC NMR (400.1 MHz; $CDCl_3$, 300 K) spectrum of **9**.
- fig. S53. ^{1}H,^{13}C-HMBC NMR (400.1 MHz; $CDCl_3$, 300 K) spectrum of **9**.
- fig. S54. ^{1}H NMR (400.1 MHz; [D_6]benzene, 300 K) spectrum of {$Na_8Mg_4[TMP]_{12}$[3,3″,5,5″-(1′,3′,5′-triphenylbenzene-tetra-ide)]} **12**.
- fig. S55. Section of the ^{1}H,^{1}H-COSY NMR (400.1 MHz; [D_6]benzene, 300 K) spectrum of **12** showing the cross peaks for the aromatic resonances.
- fig. S56. ^{1}H NMR (400.1 MHz; [D_6]benzene, 300 K) spectra of tpb (top; red) and **12** (bottom; blue).
- fig. S57. ^{1}H NMR (400.1 MHz; $CDCl_3$, 300 K) spectrum of 3,3″,5,5″-tetraiodo-5′-phenyl-benzene **13**.
- fig. S58. ^{13}C{^{1}H} NMR (100.6 MHz; $CDCl_3$, 300 K) spectrum of **13**.
- fig. S59. ^{1}H,^{1}H-COSY NMR (400.1 MHz; $CDCl_3$, 300 K) spectrum of **13**.
- fig. S60. ^{1}H,^{13}C-HSQC NMR (400.1 MHz; $CDCl_3$, 300 K) spectrum of **13**.
- fig. S61. ^{1}H,^{13}C-HMBC NMR (400.1 MHz; $CDCl_3$, 300 K) spectrum sections of **13**.
- fig. S62. ^{1}H NMR (400.1 MHz; [D_{12}]cyclohexane, 300 K) spectrum of **14**.
- fig. S63. ^{13}C{^{1}H} NMR (100.6 MHz; [D_{12}]cyclohexane, 300 K) spectrum of **14**.
- fig. S64. Sections of the ^{1}H,^{1}H-COSY NMR (400.1 MHz; [D_{12}]cyclohexane, 300 K) spectrum of **14**.
- fig. S65. Sections of the phase-sensitive ^{1}H,^{13}C-HSQC NMR (400.1 MHz; [D_{12}]cyclohexane, 300 K) spectrum of **14**.
- fig. S66. Sections of the ^{1}H,^{13}C-HMBC NMR (400.1 MHz; [D_{12}]cyclohexane, 300 K) spectrum of **14**.
- fig. S67. ^{13}C{^{1}H} NMR (100.6 MHz; [D_{12}]cyclohexane, 300 K) section of the spectra of biphenylene (top; red) and isolated **14** (bottom; blue, major conformer).
- fig. S68. ^{1}H NMR (400.1 MHz; $CDCl_3$, 300 K) spectrum of **15**.

- fig. S69. $^{13}C\{^{1}H\}$ NMR (100.6 MHz; $CDCl_3$, 300 K) spectrum of **15**.
- fig. S70. $^{1}H,^{1}H$-COSY NMR (400.1 MHz; $CDCl_3$, 300 K) spectrum of **15**.
- fig. S71. $^{1}H,^{13}C$-HSQC NMR (400.1 MHz; $CDCl_3$, 300 K) spectrum of **15**.
- fig. S72. $^{1}H,^{13}C$-HMBC NMR (400.1 MHz; $CDCl_3$, 300 K) spectrum of **15**.
- fig. S73. ^{1}H NMR (400.1 MHz; $CDCl_3$, 300 K) spectra of biphenylene (top; red) and **15** (bottom; blue).
- fig. S74. $^{13}C\{^{1}H\}$ NMR (100.6 MHz; $CDCl_3$, 300 K) spectra of biphenylene (top; red) and **15** (bottom; blue).
- fig. S75. ^{1}H NMR (400.1 MHz; $CDCl_3$, 300 K) spectrum of **16**.
- fig. S76. $^{13}C\{^{1}H\}$ NMR (100.6 MHz; $CDCl_3$, 300 K) spectrum of **16**.
- fig. S77. $^{1}H,^{1}H$-COSY NMR (400.1 MHz; $CDCl_3$, 300 K) spectrum of **16**.
- fig. S78. $^{1}H,^{13}C$-HSQC NMR (400.1 MHz; $CDCl_3$, 300 K) spectrum of **16**.
- fig. S79. $^{1}H,^{13}C$-HMBC NMR (400.1 MHz; $CDCl_3$, 300 K) spectrum of **16**.
- table S1. Metalation conditions and scope.
- References (*53–55*, *57–62*)

Supplementary Materials and methods

General methods and chemicals

All reactions and manipulations were performed under a protective atmosphere of dry pure argon gas using standard Schlenk techniques unless otherwise stated. *n*-Hexane was dried by heating to reflux over sodium benzophenone ketyl and used as freshly distilled under nitrogen atmosphere. Methylcyclohexane was distilled over sodium metal under nitrogen and then stored with activated 4 Å molecular sieves under argon prior to use. Anhydrous *N,N*-dimethylformamide (DMF) was purchased from Aldrich and used without further purification. [D_{12}]cyclohexane and [D_6]benzene were degassed and stored over activated 4 Å molecular sieves under argon prior to use. 2,2,6,6-tetramethylpiperidine [TMP(H)] was purchased from Merck KGaA and stored over activated molecular sieves (4 Å). ^{n}BuLi (1.6M in hexanes) and $^nBu_2Mg$ (1M in heptane) solutions were purchased from Aldrich and titrated prior to use. Copper iodide, carbazole, potassium carbonate, 18-crown-6, anhydrous *N,N*-dimethylformamide, sodium thiosulfate, iodine, anhydrous magnesium sulfate, ammonium chloride and sodium chloride were purchased from Aldrich and used as received. ^{n}BuNa (*50*), NaTMP (*51*) and ^{n}BuMgTMP (*29*) were prepared according to literature methods and used as solids from a glove-box or in situ. Sodium *tert*-butoxide was purchased from Aldrich and dried under vacuum at 150ºC in an Schlenk tube for 30 min prior to use. Biphenyl, *meta*-terphenyl (1,3-diphenylbenzene), *para*-terphenyl (1,4-diphenylbenzene) and 1,3,5-triphenylbenzene were purchased from Aldrich and used without further purification. Biphenylene was prepared according to the reported method and used as a crystalline solid (*4*). ^{n}BuNa, NaTMP, ^{n}BuMgTMP and the organometallic products **2**, **4**, **6**, **12** and **14** were isolated and handled inside an argon-filled glove-box whereas the derivatives **8** and **10** were *in situ* prepared and used as methylcyclohexane solutions. Organic products **3**, **5**, **7**, **8**, **9**, **11**, **13**, **15**, and **16** were isolated without the need of protocols to exclude atmospheric oxygen or moisture. NMR spectra were recorded on a Bruker DPX 400 MHz spectrometer, operating at 400.1 and 100.6 MHz for ^{1}H and ^{13}C NMR, respectively. The NMR assignments were performed using ^{13}C{^{1}H}-DEPT135, ^{1}H,^{1}H-COSY, ^{1}H,^{13}C-HSQC and ^{1}H,^{13}C-HMBC experiments. ^{1}H and ^{13}C{^{1}H} chemical shifts are expressed in parts per million (δ = ppm) and referenced to residual solvent peaks. All coupling constants (*J*) are absolute values (Hz) and the description of signals are: br for broad, s for singlet, d for doublet, dd for doublet of doublets, t for triplet, tt for triplet of triplets, q for quartet and m for multiplet. Elemental analyses (triplicate) were carried out using a Perkin Elmer 2400 elemental analyzer. Low resolution mass spectra (LRMS) for products **3**, **5**, **7**, **9**, **11**, **13**, **15**, and **16** were recorded on either an Agilent 7890A instrument coupled to an Agilent 5975C mass spectrometer (GC-MS, either chemical or electron ionization mode) with helium as a carrier gas at 1ml/min and using an Restek Rxi-5Sil-MS column (30m x 250µm x 0.25µm) or Kratos-Shimadzu Axima-CFR MALDI-TOF spectrometer (no

matrix) in positive ion mode. Accurate mass spectra were obtained at the University of Swansea in the EPSRC National Mass Spectrometry Facility, and spectra were recorded using either a Waters GCT Premier benchtop orthogonal acceleration time-of-flight (oa-TOF) mass spectrometer (electron impact ionization mode) or Waters Xevo G2-S using the atmospheric solids analysis probe (ASAP) in positive mode. Values are reported as a ratio mass to charge (m/z).

Synthetic procedures

Preparation of metalating reagent [$Na_4Mg_2(TMP)_6(^nBu)_2$] (1)

Solutions of [$Na_4Mg_2(TMP)_6Bu_2$] **1** (0.05M and 0.066M in methylcyclohexane) were prepared *in situ* according to literature methods (35, *51*). Detailed procedures are given for the preparation of **2-17**.

Note: Solutions of the metalating reagent [$Na_4Mg_2(TMP)_6Bu_2$] **1** can be prepared using an user-friendly one-pot 'glove-box-free' methodology if required (*29*).

Organometallic derivatives

Detailed synthetic protocols are given for each organometallic derivative. Notice that the organometallic derivatives **8** and **10** are highly soluble in commonly used hydrocarbons (*e.g.* methylcyclohexane, *n*-hexane and pentane) hampering their crystallization and isolation. However, the NMR spectroscopic analyses in [D_{12}]cyclohexane for the *in situ* reaction mixtures that yield **8** and **10** in methylcyclohexane solution are in agreement with the proposed formulations. Therefore, the *in situ* preparation of **8** and **10**, and their NMR characterization are included in this section. Each [Mg] empirically represents [$Na_2Mg(TMP)_3$] fragments in the formula diagrams for the organometallic compounds **2**, **4**, **6**, **8**, **10**, **12** and **14**.

Compound 2: [$Na_4Mg_2(TMP)_6$(3,5-biphenyl-di-ide)]

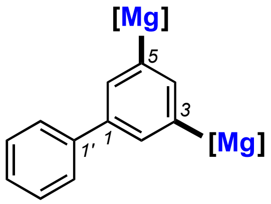

compound **2**

Preparation. In an argon-filled Schlenk tube, freshly prepared ^{n}BuNa (320 mg, 4 mmol) was suspended in methylcyclohexane (17 mL) and TMP(H) (1.02 mL, 6 mmol) was then added via syringe to produce NaTMP as a pale yellow suspension which was stirred for 30 min at ambient temperature. Commercial $^nBu_2Mg$ (2 mL, 1M solution in *n*-heptane, 2 mmol) was then added via syringe to give a 0.05M pale yellow solution of [$Na_4Mg_2(TMP)_6(^nBu)_2$] **1** in methylcyclohexane. The metalating reagent **1** was stirred for 30 min at ambient temperature prior to use. Then, biphenyl (154 mg, 1 mmol) was added and the resulting reaction mixture was heated to 65°C for 16 h to give a pale brown suspension. NMR spectroscopic analyses of aliquots of the *in situ* reaction mixture are in agreement with the presence of **2** as the major organometallic species in solution. The pale brown suspension was left at ambient temperature for 24 h and then it was filtered using standard Schlenk techniques. The collected solid was washed with *n*-hexane (3 x 5 mL) and dried under vacuum for 20 min to give **2** as a pale brown solid (870 mg, 0.735 mmol, 74%). Crystals of **2** suitable for an X-ray diffraction study were obtained by reacting **1** (20 mL, 0.05 M solution in methylcyclohexane) with biphenyl (154 mg, 1 mmol) under reflux for 10 min and allowing the reaction mixture to slowly cool down to ambient temperature in a Dewar containing hot

water (*c.a.* 80 °C) for a period of 48 h (823 mg, 0.695 mmol, 70%). The NMR spectra of the isolated pale brown solid and the crystalline material corresponding to that of **2** are coincident and appear to show that its X-ray structure is retained in [D_{12}]cyclohexane solution.

Physical state: Pale brown solid, colorless block-like crystals.

^{1}H NMR (400.1 MHz, [D_{12}]cyclohexane, 300 K): δ 0.45 (s, 12 H, Me-TMP), 0.88-1.30 (several m, 24 H, β-TMP), 1.02 (s, 12 H, Me-TMP), 1.38 (s overlapped with [D_{12}]cyclohexane solvent residual peak, 24 H, Me-TMP), 1.48 (s, 12 H, Me-TMP), 1.49 (s, 12 H, Me-TMP), 1.60 (m, 8 H, γ-TMP), 1.93 (m, 4 H, γ-TMP), 7.15 (tt, 1 H, ^3J(H,H) = 7.4 Hz, ^4J(H,H) = 1.2 Hz, H4'), 7.25 ("t", 2 H, ^3J(H,H) = 7.6 Hz, H3' + H5'), 7.39 (dd, 2 H, ^3J(H,H) = 7.4 Hz, ^4J(H,H) = 1.2 Hz, H2' + H6'), 7.92 (s, 2 H, H2 + H6), 8.05 (s, 1 H, H4); residual signals for methylcyclohexane C_7H_{14} solvent of crystallization, 0.88-1.30 (several m, 2.5 H, C_7H_{14}), 1.08-1.38 (m, 2 H, C_7H_{14}), 1.59-1.70 (m, 2.5 H, C_7H_{14}).

$^{13}C\{^1H\}$ NMR (100.6 MHz, [D_{12}]cyclohexane, 300 K): δ 19.6 (γ-TMP), 20.9 (γ-TMP), 23.2 (Me, C_7H_{14}), 27.2 (CH_2, C_7H_{14}), 27.3 (CH_2, C_7H_{14}), 32.7 (Me-TMP), 33.0 (Me-TMP), 33.7 (CH, C_7H_{14}), 36.3 (CH_2, C_7H_{14}), 38.0 (Me-TMP), 38.8 (Me-TMP), 39.4 (Me-TMP), 40.7 (Me-TMP), 41.9 (β-TMP), 42.4 (β-TMP) 42.7 (β-TMP), 42.8 (β-TMP), 51.9 (α-TMP), 52.7 (α-TMP) 52.8 (α-TMP), 53.0 (α-TMP), 127.2 (CH4'), 127.8 (CH2' + CH6'), 128.9 (CH3' + CH5'), 140.1 (CH2 + CH6), 141.7 (C1), 142.9 (C1'), 151.6 (CH4), 166.3 (C3-Mg + C5-Mg).

Elemental analysis Calcd (Found) for $C_{69.5}H_{123}Mg_2N_6Na_4$ [**2**·$(C_7H_{14})_{0.5}$]: C, 70.54 (70.17); H, 10.48 (10.70); N, 7.10% (6.86%).

Residual NMR spectroscopic signals for methylcyclohexane (C_7H_{14}), solvent of crystallization present in the crystal lattice of **2**, are also present in the [D_{12}]cyclohexane solutions of **2**. Elemental analyses in combination with NMR spectroscopy reveal that the methylcyclohexane solvent of crystallization is only partially removed under vacuum during the isolation of **2**. Isolated compound **2** exhibits low solubility in [D_{12}]cyclohexane; however, it was fully characterized by ^{1}H and ^{13}C NMR spectroscopy. Residual signals corresponding to biphenyl and TMP(H) are present within the [D_{12}]cyclohexane solutions of **2** due to unavoidable hydrolysis and low solubility of crystalline **2** in [D_{12}]cyclohexane.

Compound 4: [$Na_4Mg_2(TMP)_6$(3,5-*para*-terphenyl-di-ide)]

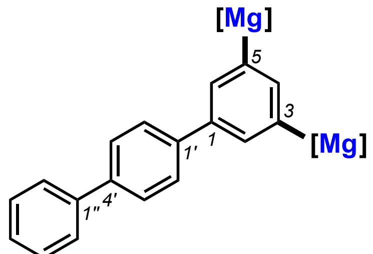

compound **4**

Preparation. In an argon-filled Schlenk tube, freshly prepared ^{n}BuNa (320 mg, 4 mmol) was suspended in methylcyclohexane (17 mL) and TMP(H) (1.02 mL, 6 mmol) was then added via syringe to produce NaTMP as a pale yellow suspension which was stirred for 30 min at room temperature. Commercial ^{n}Bu$_2$Mg (2 mL, 1M solution in *n*-heptane, 2 mmol) was then added via syringe to give a 0.05M

pale yellow solution of $[Na_4Mg_2(TMP)_6(^nBu)_2]$ **1** in methylcyclohexane. The metalating reagent **1** was stirred for 30 min at ambient temperature prior to use it. Then, *para*-terphenyl (230.3 mg, 1 mmol) was added and the resulting reaction mixture was heated to 45°C for 48 h to give a light pale brown suspension. The pale brown suspension was left at ambient temperature for 24 h and then filtered used standard Schlenk techniques. The collected solid was washed with *n*-hexane (3 x 5 mL) and dried under vacuum for 20 min to give **4** as a pale brown solid (320 mg, 0.264 mmol, 26%). Several attempts to crystallize **4** were unsuccessful, yielding microcrystalline materials (*c.a.* 15-34%) not suitable for a single crystal X-ray diffraction study.

Physical state: Pale brown solid.

^{1}H NMR (400.1 MHz, [D$_{12}$]cyclohexane, 300 K): *δ* 0.36 (s, 6 H, Me-TMP), 0.49 (s, 12 H, Me-TMP), 0.85-1.67 (several m, 82 H, Me-TMP + β-TMP + γ-TMP, overlapped with residual peaks for TMP(H) and C_7H_{14}), 1.96 (m, 8 H, γ-TMP), 7.22 (tt, 1 H, ^3J(H,H) = 7.0 Hz, ^4J(H,H) = 1.0 Hz, H4’), 7.32 (m, 2 H, H3’’ + H5’’), 7.47 (d, 2 H, ^3J(H,H) = 6.8 Hz, H2’ + H6’ or H3’ + H5’), 7.53 (dd, 2 H, ^3J(H,H) = 7.0 Hz, ^4J(H,H) = 0.8 Hz, H2’’ + H6’’), 7.56 (d, 2 H, ^3J(H,H) = 7.0 Hz, H2’ + H6’ or H3’ + H5’), 7.96 (s, 2 H, H2 + H6), 8.08 (s, 1 H, H4).

Elemental analysis Calcd (Found) for $C_{72}H_{120}Mg_2N_6Na_4$ [**4**]: C, 71.45 (71.62); H, 9.99 (9.71); N, 6.94% (6.66%).

Present within the [D$_{12}$]cyclohexane solution of **4** are also residual peaks for TMP(H) and *para*-terphenyl which is a result of unavoidable hydrolysis in [D$_{12}$]cyclohexane solution. Attempts to obtain a ^{13}C{^{1}H} NMR in [D$_{12}$]cyclohexane were unsuccessful due to the low solubility of isolated **4** in [D$_{12}$]cyclohexane.

Compound 6 $[Na_8Mg_4(TMP)_{12}$(3,3’,5,5’-*para*-terphenyl-tetra-ide)]

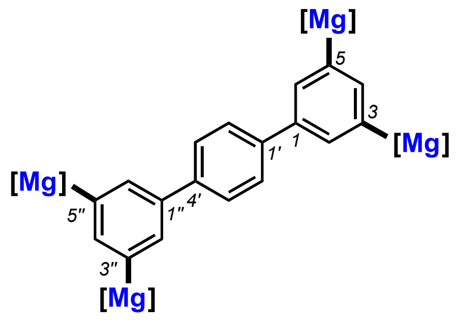

compound **6**

Preparation. In an argon-filled Schlenk tube, freshly prepared ^{n}BuNa (640 mg, 8 mmol) was suspended in methylcyclohexane (24 mL) and TMP(H) (2.04 mL, 12 mmol) was then added via syringe to give NaTMP as a pale yellow suspension which was stirred for 30 min at ambient temperature. Commercial $^nBu_2Mg$ (4 mL, 1M solution in *n*-heptane, 4 mmol) was then added via syringe to give a 0.067M pale yellow solution of $[Na_4Mg_2(TMP)_6(^nBu)_2]$ **1** in methylcyclohexane. The metalating reagent **1** was stirred for 30 min at ambient temperature prior to use it. Then, *para*-terphenyl (230.3 mg, 1 mmol) was added and the resulting reaction mixture was

heated to 65°C for 16 h to give a pale brown suspension. The pale brown suspension was filtered used standard Schlenk techniques. The collected solid was washed with *n*-hexane (3 x 5 mL) and dried under vacuum for 30 min to give **6** as a pale brown solid (1.23 g, 0.583 mmol, 59%). Crystals of **6** suitable for an X-ray diffraction study were obtained by mixing **1** (20 mL, 0.05 M solution in methylcyclohexane) with *para*-terphenyl (115.2 mg, 0.5 mmol), then heating the reaction mixture to reflux for 5 min until completely solubility of *para*-terphenyl and allowing the reaction mixture to slowly cool down to ambient temperature in a Dewar containing hot water (*c.a.* 80 °C) for a period of 48 h (710 mg, 0.334 mmol, 69%). The NMR spectra of isolated pale brown solid and crystals of **6** are coincident in [D_{12}]cyclohexane solution.

Physical state: Pale brown solid, colorless plate crystals.

^{1}H NMR (400.1 MHz, [D_{12}]cyclohexane/C_7H_{14}, 0.4/0.1 mL, 300 K): δ 0.46 (s, 24 H, Me-TMP), 0.98 (br s, 24 H, Me-TMP), 1.04 (s, 48 H, Me-TMP), 1.11-1.34 (several m, 48 H, β-TMP), 1.49 (br s, 48 H, Me-TMP), 1.65 (m, 16 H, γ-TMP), 1.94 (m, 8 H, γ-TMP), 7.41 (s, 4 H, H2' + H3' + H5' + H6'), 7.95 (s, 4 H, H2 + H6 + H2'' + H6''), 8.07 (s, 2 H, H4 + H4'). Methylcyclohexane (C_7H_{14}, *c.a.* 0.01 mL) was added to the to aid solubility of **6** in [D_{12}]cyclohexane.

Microelemental analysis Calcd (Found) for $C_{118}H_{216}Mg_4N_{12}Na_8$ [**6**]: C, 67.67 (67.49); H, 10.88 (10.25); N, 8.03% (7.81%).

Isolated compound **6** exhibits particularly low solubility in [D_{12}]cyclohexane, however, it was fully characterized by ^{1}H and ^{1}H,^{1}H-COSY NMR. Present within the [D_{12}]cyclohexane solution of **6** are also residual peaks for TMP(H) and *para*-terphenyl which is a result of unavoidable hydrolysis in [D_{12}]cyclohexane solution. Attempts to obtain a ^{13}C{^{1}H} NMR in [D_{12}]cyclohexane were unsuccessful due to its low solubility in [D_{12}]cyclohexane.

Compound 8 [$Na_4Mg_2(TMP)_6$(3,5-*meta*-terphenyl-di-ide)]

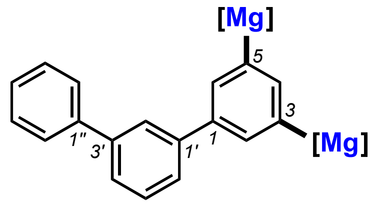

compound **8**

Preparation (*in situ*). In an argon-filled Schlenk tube, freshly prepared ^{n}BuNa (320 mg, 4 mmol) was suspended in methylcyclohexane (17 mL) and TMP(H) (1.02 mL, 6 mmol) was then added via syringe to give NaTMP as a pale yellow suspension which was stirred for 30 min at ambient temperature. Commercial $^nBu_2Mg$ (2 mL, 1M solution in *n*-heptane, 2 mmol) was then added via syringe to give a 0.05M pale yellow solution of [$Na_4Mg_2(TMP)_6(^nBu)_2$] **1** in methylcyclohexane. The metalating reagent **1** was stirred for 30 min at ambient temperature prior to use it. Then, *meta*-terphenyl (230.3 mg, 1 mmol) was added and the resulting reaction mixture was heated at 45°C for 48 h to give a dark orange solution. The solvent was removed under vacuum to give an amber color oily

material. NMR spectroscopic analyses of aliquots of the *in situ* reaction mixture and the isolated oily material are in agreement with the presence of **8** as a major organometallic species in solution (*c.a.* 64% based on *meta*-terphenyl) along with unreacted *meta*-terphenyl and/or generated by unavoidable hydrolysis of **8**. Several attempts to crystallize and isolate **8** were unsuccessful due to its high solubility in commonly used hydrocarbon solvents (methylcyclohexane, *n*-hexane, pentane); however, this *in situ* mixture of **8** can be used for electrophilic quenches without further purification.

Physical state: Dark amber oil.

^{1}H NMR (400.1 MHz, [D_{12}]cyclohexane, 300 K): *δ* 0.49 (s, 12 H, Me-TMP), 0.80-1.68 (several m, 42 H, Me-TMP + β-TMP + γ-TMP, overlapped with residual peaks for TMP(H) and C_7H_{14}), 1.03 (s, 12 H, Me-TMP), 1.04 (s, 12 H, Me-TMP), 1.46 (s, 12 H, Me-TMP), 1.48 (s, 12 H, Me-TMP), 1.91 (m, 6 H, γ-TMP), 7.21 (tt, 1 H, ^{3}J(H,H) = 7.0 Hz, ^{4}J(H,H) = 1.0 Hz, H4''), 7.28-7.44 (several m, 5 H), 7.68 (br t, 1 H, H2''), 7.96 (s, 2 H, H2 + H6), 8.08 (s, 1 H, H4).

^{13}C{^{1}H} NMR (100.6 MHz, [D_{12}]cyclohexane, 300 K): *δ* 14.5 (br s, γ-TMP), 19.7 (br, γ-TMP), 20.9 (br, γ-TMP), 32.7 (Me-TMP), 33.0 (Me-TMP), 38.9 (Me-TMP), 39.4 (Me-TMP), 40.7 (Me-TMP), 41.9 (β-TMP), 42.4 (β-TMP), 42.6 (β-TMP), 42.7 (β-TMP), 42.8 (β-TMP), 51.9 (α-TMP), 52.7 (α-TMP) 52.8 (α-TMP), 53.0 (α-TMP), 126.0 (CH), 127.4 (CH), 127.5 (CH), 129.1 (CH), 129.3 (CH), 140.2 (CH2 + CH6), 141.8 (C), 142.1 (C), 142.3 (C), 143.5 (C), 151.6 (CH4), 166.4 (C3-Mg + C5-Mg).

Present within the [D_{12}]cyclohexane solution of an *in situ* sample of **8** are also residual peaks for methylcyclohexane solvent (C_7H_{14}), and TMP(H) and *meta*-terphenyl due to unavoidable hydrolysis.

Compound 10 [$Na_8Mg_4(TMP)_{12}$(3,3',5,3'-*para*-terphenyl-tetra-ide)]

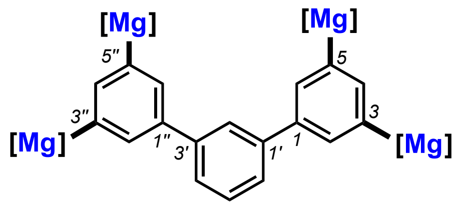

compound **10**

Preparation (*in situ*). In an argon-filled Schlenk tube, freshly prepared ^{n}BuNa (640 mg, 8 mmol) was suspended in methylcyclohexane (24 mL) and TMP(H) (2.04 mL, 12 mmol) was then added via syringe to produce NaTMP as a pale yellow suspension which was stirred for 30 min at room temperature. Commercial $^{n}Bu_2Mg$ (4 mL, 1M solution in *n*-heptane, 4 mmol) was then added via syringe to give a 0.067M pale yellow solution of [$Na_4Mg_2(TMP)_6(^{n}Bu)_2$] **1** in methylcyclohexane. The metalating reagent **1** was stirred for 30 min at ambient temperature prior to use it. Then, *meta*-terphenyl (230.3 mg, 1 mmol) was added and the resulting reaction mixture was heated at 65°C for 16 h to give a dark orange solution. The solvent was removed under vacuum to give a dark amber color oily material. NMR spectroscopic analyses of an aliquots of the *in situ* reaction mixture and the isolated oily material are in agreement with the

presence of **10** as major organometallic species in solution. Several attempts to crystallize and isolate **10** were unsuccessful due to its high solubility in methylcyclohexane; however, this *in situ* mixture of **10** can be used for electrophilic quenches without further purification.

Physical state: Dark amber oil.

^{1}H NMR (400.1 MHz, [D$_{12}$]cyclohexane, 300 K): *δ* 0.43 (br s, 12 H, Me-TMP), 0.80-1.68 (several m, 84 H, Me-TMP + β-TMP + γ-TMP, *overlapped with residual peaks for TMP(H) and C_7H_{14}*), 1.03 (br s, 24 H, Me-TMP), 1.04 (br s, 12 H, Me-TMP), 1.45 (br s, 24 H, Me-TMP), 1.46 (br s, 12 H, Me-TMP), 1.49 (br s, 24 H, Me-TMP), 1.51 (br s, 12 H, Me-TMP), 1.92 (m, 12 H, γ-TMP), 7.19-7.55 (several m, 4 H, H2' + H4' + H5' + H6', overlapped with residual peaks for *meta*-terphenyl), 7.97 (s, 2 H), 8.05 (s, 2 H), 8.12 (s, 2 H).

Present within the [D$_{12}$]cyclohexane solution of an *in situ* sample of **10** are also residual peaks for methylcyclohexane solvent (C_7H_{14}), and TMP(H) and *meta*-terphenyl due to unavoidable hydrolysis.

Compound 12 [Na$_8$Mg$_4$(TMP)$_{12}${3,3'',5,5''-(1',3',5'-triphenylbenzene-tetra-ide)}]

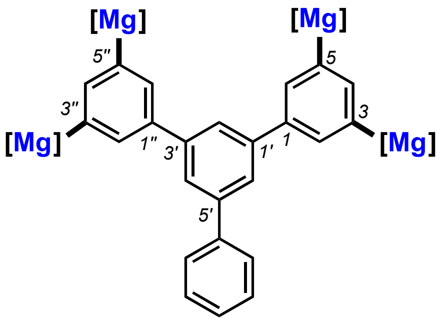

compound **12**

Preparation. In an argon-filled Schlenk tube, freshly prepared ^{n}BuNa (640 mg, 8 mmol) was suspended in methylcyclohexane (24 mL) and TMP(H) (2.04 mL, 12 mmol) was then added via syringe to produce NaTMP as a pale yellow suspension which was stirred for 30 min at ambient temperature. Commercial ^{n}Bu$_2$Mg (4 mL, 1M solution in *n*-heptane, 4 mmol) was then added via syringe to give a 0.067M pale yellow solution of [Na$_4$Mg$_2$(TMP)$_6$(^{n}Bu)$_2$] **1** in methylcyclohexane. The metalating reagent **1** was stirred for 30 min at ambient temperature prior to using it. Then, 1,3,5-triphenylbenzene (306 mg, 1 mmol) was added and the resulting reaction mixture was heated at 65°C for 24 h to give a pale brown suspension. The pale brown suspension was filtered used standard Schlenk techniques. The collected solid was washed with *n*-hexane (3 x 5 mL) and dried under vacuum for 30 min to give **12** as a brown solid (1.20 g, 0.507 mmol, 51%). Crystals of **12** suitable for an X-ray diffraction study were obtained by mixing **1** (40 mL, 0.05 M solution in methylcyclohexane) with 1,3,5-triphenylbenzene (230 mg, 0.75 mmol), then heating the reaction mixture to reflux and allowing the reaction mixture first to slowly cool down to ambient temperature in a Dewar containing hot water (*c.a.* 80 °C) and then allowing the reaction mixture to stand at room temperature for a week (0.93 g, 0.393 mmol, 52% based on 1,3,5-triphenylbenzene). The NMR spectra of the isolated pale brown solid and crystals of **12** are coincident in [D$_6$]benzene solution.

Physical state: Brown solid, colorless block crystals.

^{1}H NMR (400.1 MHz, [D$_6$]benzene, 300 K): δ 0.84 (v br s, 24 H, Me-TMP), 1.09-1.73 (several ovalapped br m, 178 H, Me-TMP + β-TMP + γ-TMP), 1.88 (m, 14 H, γ-TMP), 7.18 (m, 1 H), 7.25 (t, 1 H, ^3J(H,H) = 7.6 Hz, H4’’’), 7.39 (t, 2 H, ^3J(H,H) = 7.6 Hz, H3’’’ + H5’’’), 7.97 (d, 2 H, ^3J(H,H) = 7.2 Hz, H2’’’ + H6’’’), 8.08 (t, 1 H, ^3J(H,H) = 7.2 Hz), 8.16 (br s, 2 H), 8.34 (br s, 2 H), 8.39 (br s, 3 H); residual signals for methylcyclohexane solvent of crystallization are present, 0.88-1.30 (several m, C_7H_{14}), 1.08-1.38 (m, C_7H_{14}), 1.59-1.70 (m, C_7H_{14}).

Microelemental analysis Calcd (Found) for $C_{132}H_{230}Mg_4N_{12}Na_8$ [**12**·(C_7H_{14})]: C, 70.60 (70.39); H, 10.40 (10.54); N, 7.11% (7.43%).

Residual NMR signals for methylcyclohexane (C_7H_{14}), solvent of crystallization, are also present in the [D$_6$]benzene solutions of **12**. This solvent is only partially removed under vacuum during the isolation of **12**. Elemental analyses in combination with NMR spectroscopic studies reveal that one molecule of C_7H_{14} are present within isolated compound **12** which are in agreement with the presence of C_7H_{14} molecules of crystallization in the crystal lattice of **12**. Compound **12** could not be characterized by NMR spectroscopy in [D$_{12}$]cyclohexane due to its poor solubility; however, it was characterized by ^{1}H and ^{1}H,^{1}H-COSY NMR in [D$_6$]benzene. Residual signals corresponding to 1,3,5-triphenylbenzene and TMP(H) are present within the [D$_6$]benzene solutions of **12** due to unavoidable hydrolysis and low solubility of **12** in [D$_6$]benzene. Attempts to obtain a ^{13}C{^{1}H} NMR in [D$_6$]benzene were unsuccessful due to its low solubility.

Compound 14 [Na_4Mg_2(TMP)$_6$(1,4-biphenylene-di-ide)]

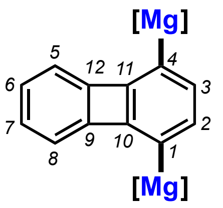

compound **14**

Preparation. In an argon-filled Schlenk tube, freshly prepared ^{n}BuNa (640 mg, 4 mmol) was suspended in methylcyclohexane (24 mL) and TMP(H) (2.04 mL, 12 mmol) was then added via syringe to give NaTMP as a pale yellow suspension which was stirred for 30 min at room temperature. Commercial ^{n}Bu$_2$Mg (4 mL, 1M solution in *n*-heptane, 4 mmol) was then added via syringe to give a 0.067M pale yellow solution of [Na_4Mg_2(TMP)$_6$(^{n}Bu)$_2$] **1** in methylcyclohexane. The metalating reagent **1** was stirred for 30 min at ambient temperature prior to using it. Then, biphenylene (304 mg, 2 mmol) was added and the resulting reaction mixture was stirred at ambient temperature for 6 h to give a light orange-brown suspension. The suspension was filtered using standard Schlenk techniques. The collected solid was washed with *n*-hexane (3 x 5 mL) and dried under vacuum for 20 min to give **14** as a pale yellow solid (1.62 g, 1.343 mmol, 67% based on biphenylene). Crystals of **14** suitable for an X-ray diffraction study were obtained by mixing **1** (10 mL, 0.1 M solution in methylcyclohexane) with biphenylene (152 mg, 1 mmol), then heating the reaction mixture to reflux and allowing the reaction mixture to slowly cool down to ambient

temperature in a Dewar containing hot water (*c.a.* 80 °C) for 48 h (730 mg, 0.605 mmol, 61% based on biphenylene). The NMR spectra of isolated pale yellow solid and crystals of **14** are coincident in [D_{12}]cyclohexane solution.

NMR spectroscopic analysis of an aliquot of the filtrate corresponding to the reaction mixture that gives **14** reveals the existence in solution of a second set of resonances, which mimicks the resonances of the isolated complex **14**, and that correspond to a second conformer of **14** (see figs. S1 and S2 for full details). A 1:0.8 ratio between the major (isolated, less soluble in methylcyclohexane) and minor (soluble in methylcyclohexane) conformers of **14** is found in the filtrate. Similar conformers have been previously seen and fully studied in related ferrocenyl inverse crown derivatives.[43]

Physical state: Pale yellow solid, pale yellow plate-like crystals.

^{1}H NMR (400.1 MHz, [D_{12}]cyclohexane, 300 K): major isolated conformer, *δ* 0.85 (s, 12 H, Me-TMP), 0.97 (s, 12 H, Me-TMP), 0.98-1.30 (several m, 24 H, β-TMP), 1.30 (s, 12 H, Me-TMP), 1.33 (s, 12 H, Me-TMP), 1.43 (s, 12 H, Me-TMP), 1.44 (s, 12 H, Me-TMP), 1.55-1.66 (m, 8 H, γ-TMP), 1.89 (m, 4 H, γ-TMP), 6.275 and 6.447 (AA' and BB' parts of an AA'BB' system, 2 H each, H5 + H6 + H7 + H8, ^3J(B,B') = 7.0 Hz, ^3J(A,A') = 6.9 Hz, ^4J(A,B') = 1.0 Hz, ^5J(A,A') = 0.5 Hz, determined with the help of a gNMR[10, 11] simulation, see fig. S3), 7.03 (s, 2 H, H2 + H3). gNMR V5.0.6 Adept Scientific plc, NMR simulation program written by P.H.M. Budzelaar, Copyright 2006 IvorySoft.

^{13}C{^{1}H} NMR (100.6 MHz, $CDCl_3$, 300 K): major isolated conformer, *δ* 19.7 (γ-TMP), 21.1 (γ-TMP), 33.3 (Me-TMP), 33.5 (Me-TMP), 38.0 (Me-TMP), 38.2 (Me-TMP), 40.0 (Me-TMP), 40.3 (Me-TMP), 42.2 (β-TMP), 42.3 (β-TMP) 42.66 (β-TMP), 42.74 (β-TMP), 52.3 (α-TMP), 52.56 (α-TMP) 52.67 (α-TMP), 52.8 (α-TMP), 118.1 (CH5 + CH8 or CH6 + CH7), 127.8 (CH5 + CH8 or CH6 + CH7), 139.9 (CH2 + CH3), 154.1 (C10 + C11), 156.8 (C9 + C12), 166.4 (C1-Mg + C4-Mg).

Analysis Calcd (Found) for $C_{71.25}H_{124.5}Mg_2N_6Na_4$ [**14**·$(C_7H_{14})_{0.75}$]: C, 70.97 (70.79); H, 10.41 (10.16); N, 6.97% (6.77%).

Compound **14** exhibits low solubility in [D_{12}]cyclohexane; however, it was fully characterized by ^{1}H and ^{13}C NMR. Residual signals corresponding to methylcyclohexane (C_7H_{14}), solvent of crystallization, and biphenylene and TMP(H) are present within the [D_{12}]cyclohexane solutions of **14** due to its unavoidable hydrolysis and low solubility. Elemental analyses in combination with NMR spectroscopy reveal that the methylcyclohexane solvent of crystallization is only partially removed under vacuum during the isolation of **2**.

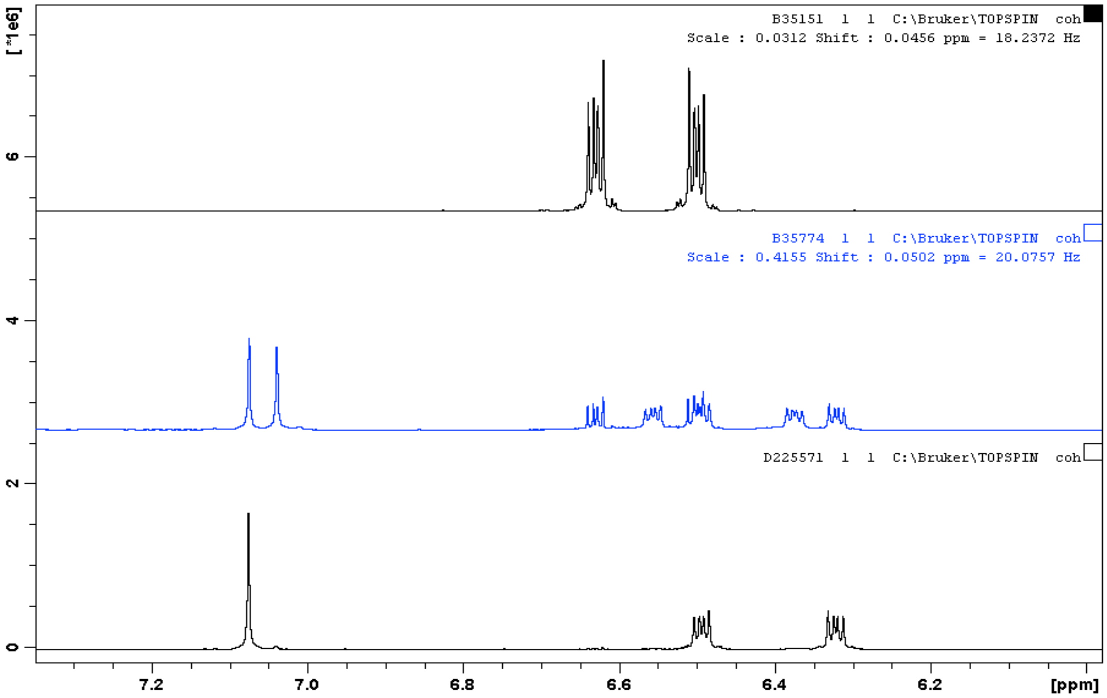

fig. S1. Sections of the ^{1}H NMR (400.1 MHz; [D_{12}]cyclohexane, 300 K) spectra showing the nonaromatic resonances for biphenylene (top; black), reaction mixture containing two conformers of 14 (middle; blue), and isolated [$Na_4Mg_2(TMP)_6$(1,4-biphenylene-di-ide)] 14 (bottom; major conformer). Residual signals for biphenylene are observed in the ^{1}H NMR spectrum of **14** in [D_{12}]cyclohexane solution due to unavoidable hydrolysis.

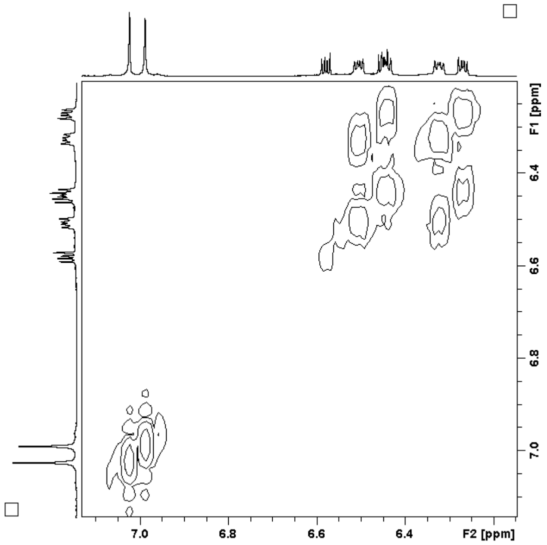

fig. S2. ^{1}H,^{1}H-COSY NMR (400.1 MHz; [D_{12}]cyclohexane, 300 K) spectrum showing the nonaromatic resonances for the two conformers of 14. Residual signals for biphenylene are observed in the ^{1}H,^{1}H-COSY NMR spectrum of the reaction mixture of **14** in [D_{12}]cyclohexane solution.

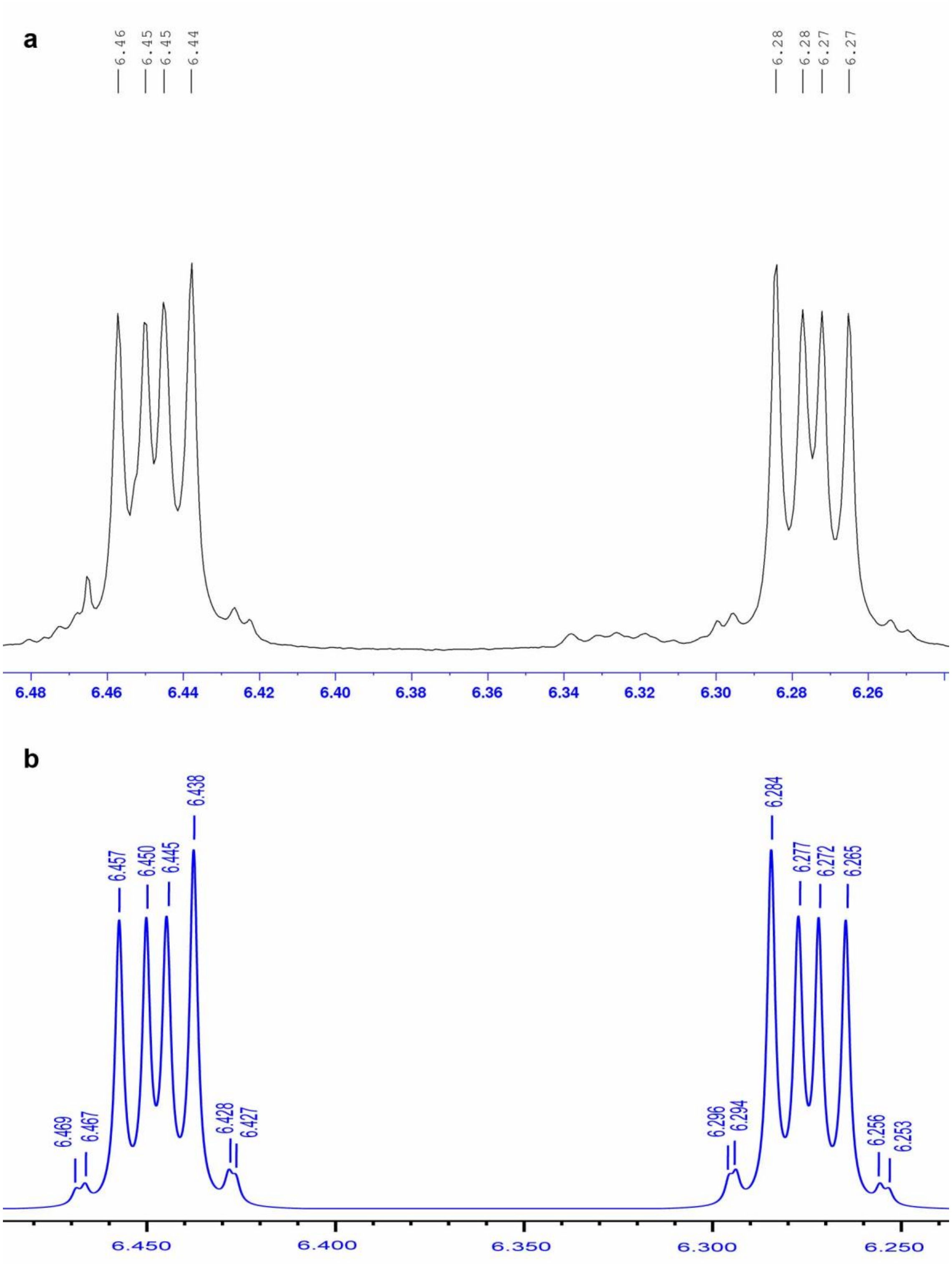

fig. S3. Sections of experimental and simulated ^{1}H NMR spectra of 14. (**a**), Experimental (top, black, 400.1 MHz, [D_{12}]cyclohexane, 300 K) and (**b**), gNMR (*56*) simulated (bottom, blue, 400.1 MHz) sections of the ^{1}H NMR spectrum of [$Na_4Mg_2(TMP)_6$(1,4-biphenylene-di-ide)] **14** showing the AA'BB' second-order spin system.

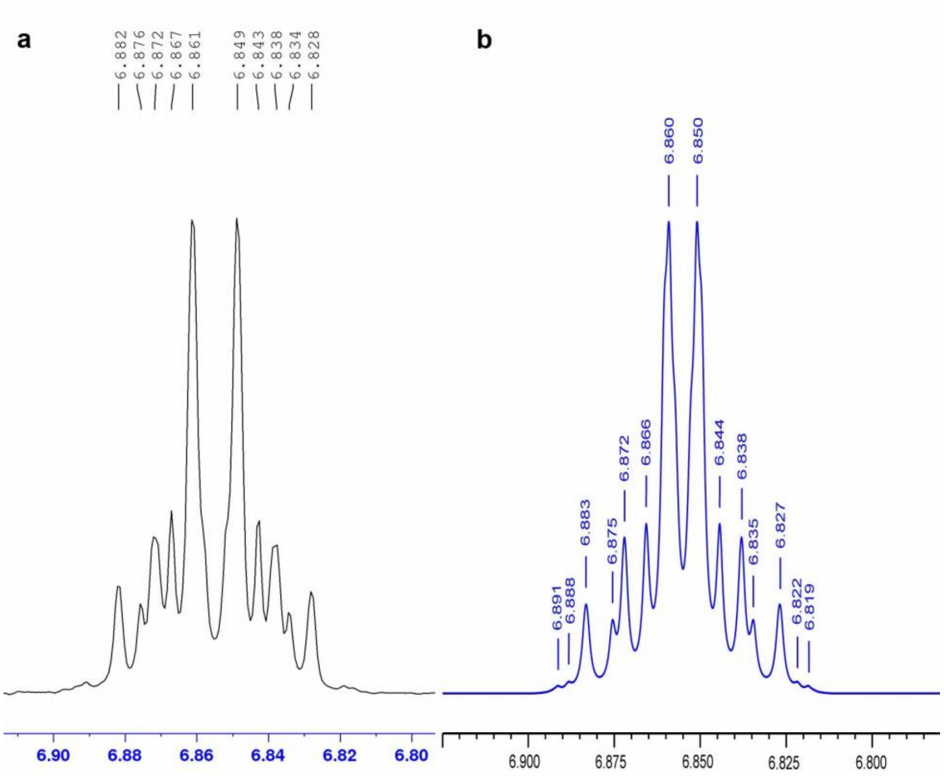

fig. S4. Sections of experimental and simulated ^{1}H NMR spectra of 15. (**a**), Experimental (left in black, 400.1 MHz, $CDCl_3$, 300 K) and (**b**), gNMR (*56*) simulated (right in blue 400.1 MHz) sections of the ^{1}H NMR spectrum of 1,4-diiodobiphenylene **15** showing the AA'BB' second-order spin system.

Electrophilic quenches

General synthetic procedures. The corresponding organic substrate (biphenyl, *para*-terphenyl, *meta*-terphenyl, 1,3,5-triphenylbenzene and biphenylene; 1 mmol each) was treated with an *in situ* solution of **1** in methylcyclohexane prepared prepared according to the synthetic organometallic protocols described above for **2**, **4**, **6**, **8**, **10**, **12**, and **14**. 0.05M solutions of **1** in methylcyclohexane (20 mL) were used for the *in situ* preparation of **2**, **4**, **8** and **14**, while 0.066M solutions of **1** in methylcyclohexane (30 mL) were employed for the *in situ* preparation of **6**, **10** and **12**. All reactions were stirred at/for different temperatures/times and *in situ* monitored by NMR spectroscopy in either [D_{12}]cyclohexane or [D_6]benzene until magnesiation of the corresponding aromatic substrate was achieved to give the corresponding organometallic derivatives (see table S1). After the *in situ* metalation step was achieved, the reaction mixture was cooled to –78 ºC in a dry ice/acetone bath for 30 mins and then added (either via cannula or disposable air-tight syringe) to an iodine solution in tetrahydrofuran (thf) at the same temperature. For the preparation of **3**, **5**, **9**, and **15**, 10 mL of a 1M solution of iodine in thf was used, whereas for **7**, **11**, and **13**, 20 mL of a 1M solution of iodine in thf was employed. The resulting reaction mixtures were stirred at –78 ºC for at least 3 h and then allowed

to slowly warm up to ambient temperature for a period of 24 h. Saturated aqueous NH_4Cl solution (*c.a.* 10 mL) was then added, followed by saturated aqueous $Na_2S_2O_3$ solution (*c.a.* 10 mL) until bleaching occurred. The crude mixture was extracted with EtOAc (3 x 20 mL) and the combined organic layers were washed with brine (20 mL) and dried over anhydrous $MgSO_4$. The solvent was removed in a rotary evaporator and the crude product was purified by flash column chromatography (silica gel, *n*-hexane/ethyl acetate) to yield the pure title compound. Alternatively, **7** can be prepared in 51% yield by reacting **6** (1 mmol) with an iodine/thf solution (20 mmol iodine/20 mL thf). The organic derivatives **3**, **5, 7**, **13** and **15** can be crystallized from either MeOH, EtOH and/or EtOAc. Compounds **7**, **11** and **13** exhibit low solubility in commonly used organic solvents which hinders their isolation and purification in the scale used in their preparations.

Notes. The chemistry involved in the tetra-metallations of *para*-terphenyl, *meta*-terphenyl and 1,3,5-triphenylbenzene to give 3,3',5,5'-tetraiodo-*para*-terphenyl **7**, 3,3',5,5'-tetraiodo-*meta*-terphenyl **11** and 3,3'',5,5''-tetraiodo-5'-phenyl-1,1':3',1''-terphenyl **13**, respectively, are worthy of further note. In the case of *para*- and *meta*-terphenyl, they were converted to the corresponding organometallic intermediates **6** and **10** in an 69% and 60% yield, respectively; and after quenching with iodine/thf, the organic products **7** and **11** were isolated in 44% and 40% yields, respectively. NMR spectroscopic studies revealed that in $CDCl_3$ solution, two different atropisomers (*57, 58*) are observed (showing identical 3,3'',5,5''-substitution patterns) which could not be physically separated. In the case of 3,3'',5,5''-tetraiodo-5'-phenyl-1,1':3',1''-terphenyl **13**, one of the two atropisomers is predominant and thus it can be purified and isolated by crystallization from ethyl acetate, and it was fully characterized by NMR spectroscopy and single crystal X-ray diffraction crystallography (see fig. S10). Terphenyl isomerism (atropisomerism) has been previously reported in related biaryl compounds (*59*–*62*).

table S1. Metalation conditions and scope.

Entry	Arene	Reaction Conditions[a]	Metalation (%)	Product (Yield, %)[d]
1	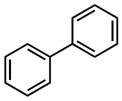 biphenyl	i) **1**, 65 ºC, 16 h<br>ii) I_2, –78 °C ⟶ r.t., 24 h	**2** (70-74%)[b]	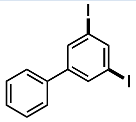 **3** (64%)
2	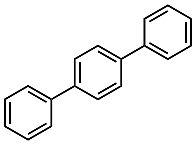 *para*-terphenyl	i) **1**, 45 ºC, 48 h<br>ii) I_2, –78 °C ⟶ r.t., 24 h	**4** (26%[b], ~70%[c])	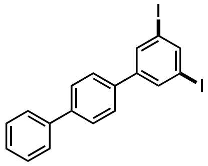 **5** (59%)
3	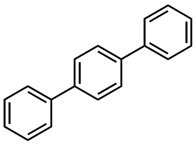 *para*-terphenyl	i) **1** (2 equiv), 65 ºC, 16 h<br>ii) I_2, –78 °C ⟶ r.t., 24 h	**6** (59-69%)[b]	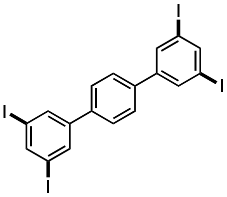 **7** (44%)
4	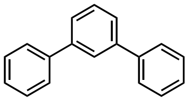 *meta*-terphenyl	i) **1**, 45 ºC, 48 h<br>ii) I_2, –78 °C ⟶ r.t., 24 h	**8** (~75%)[c]	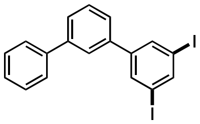 **9** (54%)
5	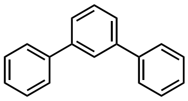 *meta*-terphenyl	i) **1** (2 equiv), 65 ºC, 16 h<br>ii) I_2, –78 °C ⟶ r.t., 24 h	**10** (~60%)[c]	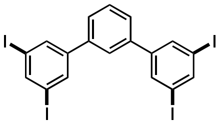 **11** (40%)
6	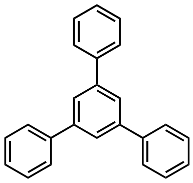 1,3,5-triphenylbenzene	i) **1** (2 equiv), 65 ºC, 24 h<br>ii) I_2, –78 °C ⟶ r.t., 24 h	**12** (52%)[b]	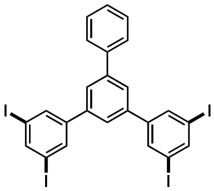 **13** (61%)
7	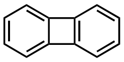 biphenylene	i) **1,** r.t, 6 h<br>ii) I_2, –78 °C ⟶ r.t., 24 h	**14** (52%)[b]	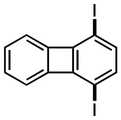 **15** (72%)
8	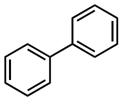 biphenyl	i) **1**, 65 ºC, 16 h<br>ii) I_2, –78 °C ⟶ r.t., 24 h<br>iii) carbazole, CuI (20% mol), K_2CO_3, 18-crown-6	**2** (67%)[b]	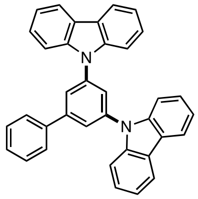 **16** (81%)[e]

[a] Temperature and time. [b] Isolated yields for the organometallic species. [c] Extent of metalation estimated by NMR spectroscopy based on *in situ* reactions of **1** and referenced to the residual signals for the corresponding substrate. [d] Isolated yields. [e] Isolation and purification of **3** is required to carry out the step iii).

Compound 3: 3,5-diiodo-1,1'-biphenyl

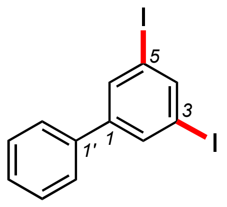

compound **3**

Purification and isolation: Flash chromatography (*n*-hexane/ethyl acetate) and crystallization either from hot EtOH or ethyl acetate by slow evaporation.

Yield: 260 mg, 0.64 mmol, 64%.

Physical state: Colorless needles.

^{1}H NMR (400.1 MHz, $CDCl_3$, 300 K): δ 7.39 (tt, 1 H, ^3J(H,H) = 7.0 Hz, ^3J(H,H) = 1.6 Hz, H4'), 7.44 (m, 2 H), 7.50 (m, 2 H), 7.88 (d, 2 H, ^3J(H,H) = 1.6 Hz, H2), 8.02 (t, 1 H, ^3J(H,H) = 1.6 Hz, H3).

^{13}C{^{1}H} NMR (100.6 MHz, $CDCl_3$, 300 K): δ 95.1 (*C*-I), 127.1 (CH2'), 128.4 (CH4'), 129.0 (CH3'), 135.5 (CH2), 138.1 (C1'), 143.6 (CH4), 145.1 (C1).

Mass Spectra: LRMS (GC-CI) *m/z* Calc. for $C_{12}H_9I_2$ $[M+H]^+$, 406.6; found, 406.8; Calc. for $C_{12}H_9I$ $[M-I+H]^+$, 279.9; found 279.7; Calc. for $C_{12}H_{10}$ $[M-2I+2H]^+$, 153.8; found 154.1; HRMS (GC-EI) *m/z* Calc. for $C_{12}H_8I_2$ $[M-1e^-]^+$, 405.8711; found, 405.8710; error, 0.2 ppm.

Compound 5: 3,5-diiodo-*para*-terphenyl

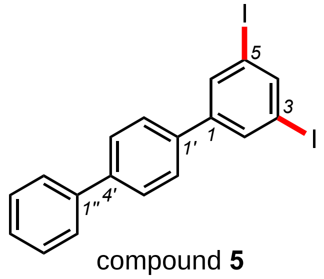

compound **5**

Purification and isolation: Flash chromatography (*n*-hexane/ethyl acetate) and crystallization from ethanol by slow evaporation.

Yield: 285 mg, 0.59 mmol, 59%.

Physical state: Colorless rods.

^{1}H NMR (400.1 MHz, $CDCl_3$, 300 K): δ 7.38 (“tt”, 1 H, ^3J(H,H) = 7.4 Hz, ^4J(H,H) = 1.2 Hz, H4''), 7.47 (m, 2 H, H3'' + H5''), 7.58 (m, 2 H, H2’ + H6’), 7.63 (m, 2 H, H2’’ + H6’’), 7.67 (m, 2 H, H3’ + H5’), 7.93 (d, 2 H, ^4J(H,H) = 1.5 Hz, H2 + H6), 8.03 (t, 1 H, ^4J(H,H) = 1.5 Hz, H4).

^{13}C{^{1}H} NMR (100.6 MHz, $CDCl_3$, 300 K): δ 95.3 (*C*-I), 127.2 (CH2'' + CH6''), 127.6 (CH2' + CH6'), 127.79 (CH4''), 127.83 (CH3' + CH5'), 129.0 (CH3'' + CH5''), 135.5 (CH2 + CH5), 140.4 (C1'), 141.4 (C1''), 142.1 (C4'), 143.8 (CH4), 144.7 (C1).

Mass Spectra: LRMS (GC-CI) *m/z* Calc. for $C_{18}H_{13}I_2$ $[M+H]^+$, 482.9; found, 482.8; Calc. for $C_{18}H_{13}I$ $[M-I+H]^+$, 356.0; found 355.9; Calc. for $C_{18}H_{14}$ $[M-2I+2H]^+$, 230.1; found 230.0; HRMS (GC-EI) *m/z* Calc. for $C_{18}H_{12}I_2$ $[M-1e^-]^+$, 481.9023; found, 481.9027; error, 0.8 ppm.

Compound 7: 3,3',5,5'-tetraiodo-*para*-terphenyl

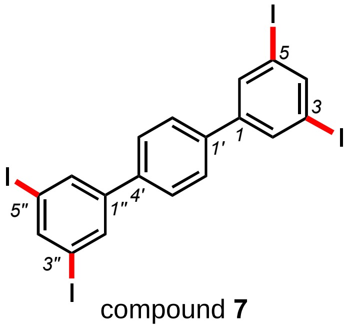

compound **7**

Purification and isolation: Flash chromatography (*n*-hexane/ethyl acetate) and crystallization from ethyl acetate by slow evaporation.

Yield: 320 mg, 0.44 mmol, 44%.

Physical state: Colorless solid.

^{1}H NMR (400.1 MHz, $CDCl_3$, 300 K): δ, atropisomers in a 1:0.5 ratio, 7.38 (m, 2 H), 7.55-7.66 (several m, 7 H), 7.91 (d, 2 H, ^{4}J(H,H) = 1.6 Hz, H2 + H6 + H2'' +H6'', major isomer), 7.92 (d, 1 H, ^{4}J(H,H) = 1.6 Hz, H2 + H6 + H2'' +H6'', minor isomer), 7.93 (d, 1 H,, H2 + H6 + H2'' +H6'' ^{4}J(H,H) = 1.6 Hz, minor isomer), 8.03 (m, overlapped t, 2 H, H4 + H4'').

^{13}C{^{1}H} NMR (100.6 MHz, $CDCl_3$, 300 K): δ 95.0 (*C*-I, minor isomer), 95.4 (*C*-I, major isomer), 126.5 (CH), 126.9 (CH), 127.2 (CH), 127.6 (CH), 127.7 (CH), 127.8 (CH), 129.1 (CH) 129.9 (CH), 130.6 (CH), 135.5 (CH), 135.6 (CH), 136.1 (C), 143.8 (C), 143.9 (C), 144.0 (C), 144.5 (C).

Mass Spectra: LRMS (GC-CI) *m/z* Calc. for $C_{18}H_{11}I_4$ $[M+H]^+$, 734.7; found, 734.5; Calc. for $C_{18}H_{11}I_3$ $[M\text{-}I+H]^+$, 607.8; found 607.7; Calc. for $C_{18}H_{12}I_2$ $[M\text{-}2I+2H]^+$, 481.9; found 481.8; (GC-EI) *m/z* Calc. for $C_{18}H_{10}I_4$ $[M\text{-}1e^-]^+$, 733.7; found, 733.9; Calc. for $C_{18}H_{10}I_3$ $[M\text{-}I\text{-}1e^-]^+$, 606.8; found 606.8; Calc. for $C_{18}H_{10}I_2$ $[M\text{-}2I\text{-}1e^-]^+$, 480.0; found 479.9; HRMS (GC-EI) *m/z* Calc. for $C_{18}H_{10}I_4$ $[M\text{-}1e^-]^+$, 733.6956; found, 733.6952, error, 0.5 ppm.

Compound 9: 3,5-diiodo-*meta*-terphenyl

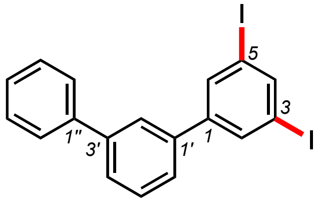

compound **9**

Purification and isolation: Flash chromatography (*n*-hexane/ethyl acetate).

Yield: 260 mg, 0.54 mmol, 54%.

Physical state: Pale yellow oil.

^{1}H NMR (400.1 MHz, $CDCl_3$, 300 K): δ 7.38 (t, 1 H, ^{3}J(H,H) = 7.2 Hz, ^{4}J(H,H) = 1.0 Hz, H4''), 7.46-7.50 (m, 4 H), 7.51 (t, 1 H, ^{3}J(H,H) = 7.2 Hz, H5'), 7.63 (m, 2 H), 7.68 (t, 1 H, ^{4}J(H,H) = 1.6 Hz, H2'), 7.93 (d, 2 H, ^{4}J(H,H) = 1.5 Hz, H2 + H6), 8.04 (t, 1 H, ^{4}J(H,H) = 1.5 Hz, H4).

^{13}C{^{1}H} NMR (100.6 MHz, $CDCl_3$, 300 K): δ 95.3 (*C*-I), 126.1 (2 x CH), 127.28 (CH2'), 127.34 (2 x CH), 127.8 (CH4), 129.0 (2 x CH), 129.5 (CH5'), 135.6 (CH2 + CH6), 138.7 (C), 140.7 (C), 142.2 (C), 143.8 (CH4), 144.1 (C).

Mass Spectra: LRMS (GC-EI) *m*/*z* Calc. for $C_{18}H_{12}I_2$ [M-e$^-$]$^+$, 481.9; found, 481.9; Calc. for $C_{18}H_{13}I$ [M-I+H-e$^-$]$^+$, 356.0; found 356.1; Calc. for $C_{18}H_{12}$ [M-2I-e$^-$]$^+$, 228.1; found 228.1; HRMS (GC-EI) *m*/*z* Calc. for $C_{18}H_{12}I_2$ [M-1e$^-$]$^+$, 481.9023; found, 481.9027; error, 0.8 ppm.

Compound 11: 3,3',5,5'-tetraiodo-*meta*-terphenyl

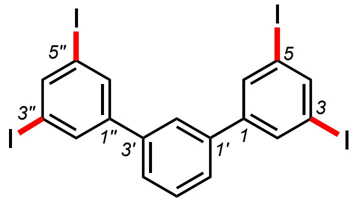

compound **11**

Purification and isolation: Flash chromatography (*n*-hexane/ethyl acetate).

Yield: 290 mg, 0.40 mmol, 40%.

Physical state: Pale yellow solid.

^{1}H NMR (400.1 MHz, CDCl$_3$, 300 K): *δ*, atropysomers in a 1:1 ratio, 7.31 (d, 2 H, ^{3}J(H,H) = 8.4 Hz), 7.51 (d, 3 H, ^{4}J(H,H) = 1.6 Hz), 7.57 (“t”, 2 H, ^{4}J(H,H) = 1.2 Hz), 7.79 (d, 4 H, ^{3}J(H,H) = 8.2 Hz), 7.85 (d, 2 H, ^{3}J(H,H) = 1.6 Hz, H2 + H6 + H2'' +H6''), 7.90 (d, 4 H, ^{3}J(H,H) = 1.6 Hz, H2 + H6 + H2'' +H6''), 8.06 (m, overlapped t, 3 H, H4 + H4'').

^{13}C{^{1}H} NMR (100.6 MHz, CDCl$_3$, 300 K): *δ* 95.4 (*C*-I), 125.9 (CH), 126.1 (CH), 127.2 (CH), 127.4 (CH), 127.8 (CH), 129.0 (CH), 129.1 (CH) 129.5 (CH), 135.6 (CH), 135.7 (CH), 138.2 (C), 143.9 (C), 144.2 (C), 144.6 (C).

Mass Spectra: LRMS (GC-CI) *m*/*z* Calc. for $C_{18}H_{11}I_4$ [M+H]$^+$, 734.7; found, 734.6; Calc. for $C_{18}H_{11}I_3$ [M-I+H]$^+$, 607.8; found 607.8; Calc. for $C_{18}H_{12}I_2$ [M-2I+2H]$^+$, 481.9; found 481.8; ; (GC-EI) *m*/*z* Calc. for $C_{18}H_{10}I_4$ [M+H-1e$^-$]$^+$, 733.7; found, 734.0; Calc. for $C_{18}H_{10}I_3$ [M-I-1e$^-$]$^+$, 606.8; found 607.0; Calc. for $C_{18}H_{10}I_2$ [M-2I-1e$^-$]$^+$, 480.0; found 479.9; HRMS (GC-EI) *m*/*z* Calc. for $C_{18}H_{10}I_4$ [M-1e$^-$]$^+$, 733.6956; found, 733.6951, error, 0.5 ppm.

Compound 13: 3,3'',5,5''-tetraiodo-5'-phenyl-1,1':3',1''-terphenyl

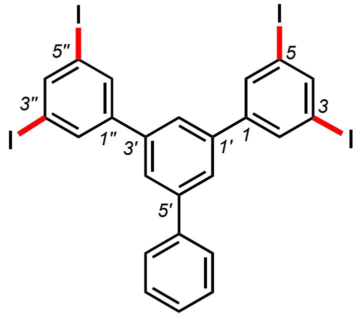

compound **13**

Purification and isolation: Flash chromatography (*n*-hexane/ethyl acetate) and crystallization for ethyl acetate by slow evaporation.

Yield: 490 mg, 0.61 mmol, 61%.

Physical state: Colorless needles.

^{1}H NMR (400.1 MHz, CDCl$_3$, 300 K): *δ* 7.42 (tt, 1 H, ^{3}J(H,H) = 7.2 Hz, ^{4}J(H,H) = 1.2 Hz, H4'''), 7.50 (m, 2 H, H3''' + H5'''), 7.54 (t, 1 H, ^{4}J(H,H) = 1.6 Hz, H2'), 7.65 (dd, 2 H, ^{3}J(H,H) = 7.4 Hz, ^{4}J(H,H) = 1.2 Hz, H2''' + H6'''), 7.69 (d, 2 H, ^{4}J(H,H) = 1.6 Hz, H4' + H6'), 7.95 (d, 4 H, ^{4}J(H,H) = 1.5 Hz, H2 + H6 + H2'' + H6''), 8.08 (t, 2 H, ^{4}J(H,H) = 1.5 Hz, H4 + H4').

$^{13}C\{^1H\}$ NMR (100.6 MHz, $CDCl_3$, 300 K): δ 95.4 (*C*-I, C3 + C5 + C3'' + C5''), 124.9 (CH2'), 126.2 (CH4' + CH6'), 127.5 (CH2'' + CH6''), 128.2 (CH4''), 129.1 (CH3''' + CH5'''), 135.8 (CH2 + CH6 + CH2'' + CH6''), 139.8 (C1' + C3'), 140.3 (C1'''), 143.2 (C5'), 144.4 (CH4 + CH4''), 144.7 (C1' + C1'').

Mass Spectra: LRMS (MALDI-EI) *m/z* Calc. for $C_{24}H_{14}I_4$ [M-1e$^-$]$^+$, 809.7; found, 809.8; Calc. for $C_{24}H_{13}I_3$ [M-H-I-1e$^-$]$^+$, 681.8; found 682.0; Calc. for $C_{24}H_{16}I_2$ [M-2I+2H]$^+$, 557.9; found 557.9; Calc. for $C_{24}H_{17}I$ [M-2I+3H]$^+$, 432.0; found 432.1; HRMS (GC-EI) *m/z* Calc. for $C_{24}H_{14}I_4$ [M-1e$^-$]$^+$, 809.7269; found, 809.7266, error, 0.4 ppm; $C_{24}H_{15}I_4$ [M+H-1e$^-$]$^+$, 810.7347; found, 810.7344, error, 0.4 ppm.

Compound 15: 1,4-diiodobiphenylene

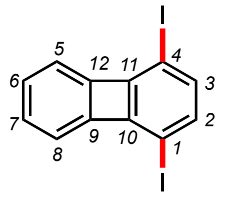

compound **15**

Purification and isolation: Flash chromatography (*n*-hexane/ethyl acetate) and crystallization from ethyl acetate by slow evaporation.

Yield: 290 mg, 0.72 mmol, 72%.

Physical state: Pale yellow needles.

^1H NMR (400.1 MHz, $CDCl_3$, 300 K): δ 6.65 (s, 2 H), 6.843 and 6.867 (AA' and BB' parts of an AA'BB' system, 2 H each, H5 + H6 + H7 + H8, ^3J(A,B) = 7.8 Hz, ^3J(B,B') = 7.2 Hz, ^4J(A,B') = 1.4 Hz, ^5J(A,A') = 0.8 Hz, determined with the help of a gNMR[10, 11] simulation, see fig. S4).

$^{13}C\{^1H\}$ NMR (100.6 MHz, $CDCl_3$, 300 K): δ 80.0 (*C*-I), 116.9 (CH7+CH6), 129. (CH5+CH8), 137.6 (CH2+CH3), 149.4 (C9+C12), 157.9 (C10+C11).

Mass Spectra: LRMS (GC-CI) *m/z* Calc. for $C_{12}H_7I_2$ [M+H]$^+$, 404.9; found, 404.7; Calc. for $C_{12}H_7I$ [M-I+H]$^+$, 278.0; found 277.9; Calc. for $C_{12}H_8$ [M-2I+2H]$^+$, 152.1; found 152.0; HRMS (GC-EI) *m/z* Calc. for $C_{12}H_{16}I_2$ [M-1e$^-$]$^+$, 403.8554; found, 403.8562, error, 1.9 ppm.

Copper-catalysed Ullmann-type coupling of 3,5-diiodo-1,1'-biphenyl (3) with carbazole.

Synthesis of 9,9'-(3,5-biphenyl)bis(carbazole) 16. In an argon-filled Schlenk tube, 3,5-diiodo-1,1'-biphenyl (150 mg, 0.37 mmol) was mixed with carbazole (140 mg, 0.83 mmol), potassium carbonate (114 mg, 0.83 mmol), copper(I) iodide (32 mg, 0.17 mmol) and 18-crown-6 (30 mg, 0.1 mmol). Then anhydrous *N,N*-dimethylformamide (DMF) was added (5 mL) and the reaction mixture was refluxed for 24 h under argon atmosphere. After cooling down to ambient temperature, the reaction mixture was filtered throught a short pad of Celite and the solvent was removed under vacuum. The crude product was purified by flash column chromatography (silica gel, *n*-hexane/ethyl acetate) to yield the pure title compound as a pale yellow solid. **16** can be crystallized from ethyl acetate by slow evaporation to give colorless block like crystals suitable for an X-ray diffraction study.

Compound 16: 9,9'-(3,5-biphenyl)bis(carbazole)

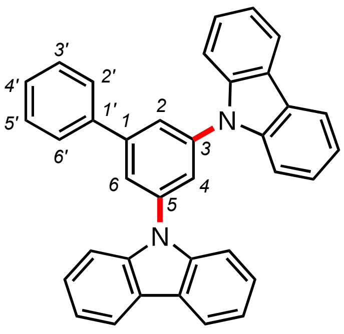

compound **16**

Purification and isolation: Flash chromatography (*n*-hexane/ethyl acetate) and crystallization from ethyl acetate by slow evaporation.

Yield: 145 mg, 0.30 mmol, 81%.

Physical state: Pale yellow solid, colorless block-like crystals.

^{1}H NMR (400.1 MHz, $CDCl_3$, 300 K): δ 7.34 ("dt", 4 H, ^3J(H,H) = 7.6 Hz, ^4J(H,H) = 1.0 Hz, carbazole), 7.42-7.53 (m, 7 H, carbazole + H3' + H4' + H5'), 7.62 (d, 4 H, ^3J(H,H) = 8.2 Hz, carbazole), 7.64 (m, 2 H, H4), 7.82 (t, 1 H, ^3J(H,H) = 1.8 Hz, H2' + H6'), 7.95 (d, 2 H, ^3J(H,H) = 1.8 Hz), 8.18 (d, 4 H, ^3J(H,H) = 7.7 Hz, carbazole).

^{13}C{^{1}H} NMR (100.6 MHz, $CDCl_3$, 300 K): δ 109.9 (CH, carbazole), 120.5 (CH, carbazole), 120.6 (CH, carbazole), 123.8 (C, carbazole), 123.9 (CH4), 124.5 (CH2 + CH6), 126.4 (CH, carbazole), 127.3 (CH2' + CH6'), 128.6 (CH4'), 129.3 (CH3' + CH5'), 139.4 (C1'), 139.9 (C-N), 140.8 (C-N, carbazole), 144.9 (C1).

Mass Spectra: LRMS (GC-EI) *m*/*z* Calc. for $C_{36}H_{24}N_2$ [M-1e$^-$]$^+$, 484.2; found, 484.2; Calc. for $C_{24}H_{15}N$ [M-$C_{12}H_9$N1e$^-$]$^+$, 317.1; found 317.3.

X-ray crystallography

Crystal structure determinations

Single-crystal X-ray diffraction intensities were collected at 123(2) K on Oxford Diffraction Xcalibur and Gemini diffractometers with Mo-*Kα* (λ = 0.71073 Å; for **2**, **3**, **14** and **15**) and Cu-*Kα* (λ = 1.54184 Å; for **12** and **16**) radiation, respectively; and Bruker Apex II diffractometer with an Incotec IµS Mo-*Kα* microsource (λ = 0.71073 Å; for **5**, **6** and **13**). The structures were solved by classical direct methods using SHELXS (version 2013/1) and refined to convergence on F^2 and against all independent reflections by full-matrix least-squares and SHELXL (version 2014/7) programs (*53*), in combination with the molecular graphical user interface SHELXLE (*54*). All non-hydrogen atoms were refined anisotropically and hydrogen atoms were geometrically placed and allowed to ride on their parent atoms. Selected crystallographic data are summarized in the text and full details are given in the supplementary deposited cif files (CCDC 1516422-30). These data can be obtained free of charge from the Cambridge Crystallographic Data Centre via http://www.ccdc.cam.ac.uk/data_request/cif.

X-ray crystal structure of 2 (CCDC 1516422)

Crystal data for **2**: $C_{73}H_{130}Mg_2N_6Na_4$, M = 1232.40 g/mol, monoclinic, $C2/c$, a = 52.8064(19), b = 15.3894(3), c = 20.1528(16) Å, β = 107.089(6)°, V = 15654.3(15) Å^3, Z = 8, λ(Mo-*Kα*) = 0.71073 Å, T = 123 K, colorless block, Oxford Diffraction Xcalibur diffractometer; 129054 reflections collected, 17044 independent measured reflections (R_{int} = 0.0551), F^2 refinement, R_1(obs) = 0.0569, wR_2(all data) = 0.1574, 12393 independent observed reflections [$|F_o| > 4\sigma(|F_o|)$, 2θmax = 54°], 840 parameters, GOF = 1.045.

Additional details for **2**: One TMP ligand and one methylcyclohexane molecule of crystallization were modeled as disordered over two sites, and the geometries and displacement parameters of the disordered groups were restrained to approximate typical values. The SQUEEZE routine of PLATON-2016 (*55*) was used to remove the effects of residual electron density corresponding to disordered methylcyclohexane solvent molecules, which could not be satisfactorily modeled. Approximately 257 electron equivalents were removed from 1435 Å^3 of "void" space per unit cell. This corresponds to approximately five methylcyclohexane solvent molecules per unit cell.

checkCIF/PLATON report

Alert level B

PLAT910_ALERT_3_B Missing # of FCF Reflection(s) Below Theta(Min) 25 Note

Author Response: These statistics are acceptable given the size of the molecule and of the unit cell.

X-ray crystal structure of 3 (CCDC 1516423)

Crystal data for **3**: $C_{12}H_8I_2$, M = 405.98 g/mol, monoclinic, $C2/c$, a = 18.2121(18), b = 4.6937(4), c = 27.747(2) Å, β = 102.126(7)°, V = 2318.9(4) Å^3, Z = 8, λ(Mo-$K\alpha$) = 0.71073 Å, T = 123 K, colorless plate, Oxford Diffraction Xcalibur diffractometer; 13840 reflections collected, 2728 independent measured reflections (R_{int} = 0.0477), F^2 refinement, R_1(obs) = 0.0329, wR_2(all data) = 0.0708, 2279 independent observed reflections [$|F_o| > 4\sigma(|F_o|)$, 2θmax = 56°], 166 parameters, GOF = 1.072.

Additional details for **3**: The phenyl C_6H_5 group was modeled as disordered over two sites with 50% occupancy each, and the geometries and displacement parameter of the disordered groups were allowed to freely refine.

X-ray crystal structure of 5 (CCDC 1516424)

Crystal data for **5**: $C_{18}H_{12}I_2$, M = 482.08 g/mol, monoclinic, $C2/c$, a = 18.1628(8), b = 4.7096(2), c = 36.2054(16) Å, β = 98.916(2)°, V = 3059.6(2) Å^3, Z = 8, λ(Mo-$K\alpha$)= 0.71073 Å, T = 123 K, colourless rod, Bruker Apex II diffractometer with an Incotec IµS microsource, 49183 reflections collected, 3132 independent measured reflections (R_{int} = 0.0223), F^2 refinement, R_1(obs) = 0.0289, wR_2(all data) = 0.0609, 3097 independent observed reflections [$|F_o| > 4\sigma(|F_o|)$, 2θmax = 52.8°], 253 parameters, GOF = 1.225.

Additional details for **5**: The phenyl C_6H_5 and phenylene C_6H_4 groups were modeled as disordered over two sites with 50% occupancy each, and the geometries and displacement parameter of the disordered groups were allowed to freely refine.

X-ray crystal structure of 6 (CCDC 1516425)

Crystal data for **6**: $C_{126}H_{226}Mg_4N_{12}Na_8$, M = 2190.33 g/mol, monoclinic, $C2/m$, a = 15.8990(14), b = 37.338(3), c = 15.4630(13) Å, β = 120.261(3)°, V = 7928.6(12) Å^3, Z = 2, λ(Mo-$K\alpha$) = 0.71073 Å, T = 123 K, colorless plate, Bruker Apex II diffractometer with an Incotec IµS microsource; 42824 reflections collected, 6315 independent measured reflections (R_{int} = 0.1356), F^2 refinement, R_1(obs) = 0.0938, wR_2(all data) = 0.2115, 3669 independent observed reflections [$|F_o| > 4\sigma(|F_o|)$, 2θmax = 48°], 390 parameters, GOF = 1.050.

Additional details for **6**: One TMP ligand and the phenylene C_6H_4 group were modeled as disordered over two sites, and the geometries and displacement parameters of the disordered groups were restrained to approximate typical values. The SQUEEZE routine of PLATON-2016 (*55*) was used to remove the effects of residual electron density corresponding to disordered methylcyclohexane solvent molecules, which could not be satisfactorily modeled. Approximately 383 electron equivalents

were removed from 1875 Å^3 of "void" space per unit cell. This corresponds to approximately seven methylcyclohexane solvent molecules per unit cell.

checkCIF/PLATON report

Alert level B

THETM01_ALERT_3_B The value of sine(theta_max)/wavelength is less than 0.575

Calculated sin(theta_max)/wavelength = 0.5722

Author Response: Structure 6 is a low angle dataset from a very weakly diffracting crystal. Despite much effort, no better dataset could be obtained. As is made clear in the main body of the paper, the low resolution structure is used only for purposes of atomic connectivity.

PLAT910_ALERT_3_B Missing # of FCF Reflection(s) Below Theta(Min) 20 Note

Author Response: These statistics are acceptable given the size of the molecule and of the unit cell.

X-ray crystal structure of 12 (CCDC 1516426)

Crystal data for **12**: $C_{132}H_{230}Mg_4N_{12}Na_8$, M = 2266.43 g/mol, orthorhombic, $P2_12_12$, a = 36.6181(7), b = 26.4856(5), c = 15.3480(3) Å, $\alpha = \beta = \gamma$ = 90°, V = 14885.3(5) Å^3, Z = 4, λ(Cu-*Kα*) = 1.54184 Å, T = 123 K, colorless plate, Oxford Diffraction Gemini diffractometer; 84110 reflections collected, 27729 independent measured reflections (R_{int} = 0.0372), F^2 refinement, R_1(obs) = 0.0580, wR_2(all data) = 0.1657, 18872 independent observed reflections [$|F_o| > 4\sigma(|F_o|)$, 2θmax = 140°], 1582 parameters, GOF = 1.024, absolute structure parameter = -0.015(12).

Additional details for **12**: Two TMP ligands and the phenyl C_6H_5 group were modeled as disordered over two sites, and the geometries and displacement parameters of the disordered groups were restrained to approximate typical values. The SQUEEZE routine of PLATON-2016 (*55*) was used to remove the effects of residual electron density corresponding to disordered and partially present methylcyclohexane solvent molecules. Approximately 372 electron equivalents were removed from 1982 Å^3 of "void" space per unit cell. This corresponds to approximately six to seven methylcyclohexane solvent molecules per unit cell.

checkCIF/PLATON report

Alert level B

PLAT230_ALERT_2_B Hirshfeld Test Diff for C48 -- C49 ..7.2 s.u.

Author Response: Parts of this structure have been modelled as disordered. The displacement parameters of C48-C50 suggest that there may be a small amount of disorder present for the ligand containing these atoms and their pendant Me groups (e.g. C52, C54) too. However, this appears to be

a relatively small effect compared to the disorder elsewhere in the structure and so it was decided not to model disorder here**.**

PLAT230_ALERT_2_B Hirshfeld Test Diff for C49 -- C50 .. 7.7 s.u.

Author Response: Parts of this structure have been modelled as disordered. The displacement parameters of C48-C50 suggest that there may be a small amount of disorder present for the ligand containing these atoms and their pendant Me groups (e.g. C52, C54) too. However, this appears to be a relatively small effect compared to the disorder elsewhere in the structure and so it was decided not to model disorder here.

PLAT412_ALERT_2_B Short Intra XH3 .. XHn H52B .. H54B .. 1.77 Ang.

Author Response: Parts of this structure have been modelled as disordered. The displacement parameters of C48-C50 suggest that there may be a small amount of disorder present for the ligand containing these atoms and their pendant Me groups (e.g. C52, C54) too. However, this appears to be a relatively small effect compared to the disorder elsewhere in the structure and so it was decided not to model disorder here.

PLAT910_ALERT_3_B Missing # of FCF Reflection(s) Below Theta(Min) 44 Note

Author Response: These statistics are acceptable given the size of the molecule and of the unit cell.

X-ray crystal structure of 13 (CCDC 1516427)

Crystal data for **13**: $C_{24}H_{14}I_4$, M = 809.95 g/mol, monoclinic, $C2/c$, a = 39.991(2), b = 4.7176(3), c = 23.9638(14) Å, β = 99.865(3)°, V = 4454.2(5) Å^3, Z = 8, λ(Mo-$K\alpha$) = 0.71073 Å, T = 123 K, orange lathe, Bruker Apex II diffractometer with an Incotec IμS microsource, 35515 reflections collected, 4535 independent measured reflections (R_{int} = 0.0234), F^2 refinement, R_1(obs) = 0.0164, wR_2(all data) = 0.0397, 4189 independent observed reflections [$|F_o| > 4\sigma(|F_o|)$, 2θmax = 52.8°], 289 parameters, GOF = 1.036.

Additional details for **13**: The phenyl C_6H_5 group was modeled as disordered over two sites with 50% occupancy each, and the geometries and displacement parameter of the disordered groups were allowed to freely refine.

X-ray crystal structure of 14 (CCDC 1516428)

Crystal data for **14**: $C_{66}H_{114}Mg_2N_6Na_4$, M = 1132.21 g/mol, monoclinic, $P2_1/n$, a = 15.2188(4), b = 16.0801(5), c = 27.6416(7) Å, β = 91.434(2)°, V = 6762.3(3) Å^3, Z = 4, λ(Mo-$K\alpha$) = 0.71073 Å, T = 123 K, pale yellow plate, Oxford Diffraction Xcalibur diffractometer; 64977 reflections collected, 14654 independent measured reflections (R_{int} = 0.0314), F^2 refinement, R_1(obs) = 0.0438, wR_2(all data) =

0.1113, 11655 independent observed reflections [|F_o| > 4σ(|F_o|), 2θmax = 54°], 784 parameters, GOF = 1.041.

Additional details for **14**: Two TMP ligands were modeled as disordered over two sites, and the geometries and displacement parameters of the disordered groups were restrained to approximate typical values.

checkCIF/PLATON report

Alert level B

PLAT910_ALERT_3_B Missing # of FCF Reflection(s) Below Theta(Min) 25 Note

Author Response: These statistics are acceptable given the size of the molecule and of the unit cell.

X-ray crystal structure of 15 (CCDC 1516429)

Crystal data for **15**: $C_{12}H_6I_2$, M = 403.97 g/mol, monoclinic, $P2_1/c$, a = 4.21910(10), b = 15.0338(5), c = 16.9609(6) Å, β = 92.989(3)°, V = 1074.35(6) Å^3, Z = 4, λ(Mo-$K\alpha$) = 0.71073 Å, T = 123 K, colorless needle, Oxford Diffraction Xcalibur diffractometer; 14784 reflections collected, 2346 independent measured reflections (R_{int} = 0.0377), F^2 refinement, R_1(obs) = 0.0193, wR_2(all data) = 0.0439, 2102 independent observed reflections [|F_o| > 4σ(|F_o|), 2θmax = 54°], 127 parameters, GOF = 1.051.

X-ray crystal structure of 16 (CCDC 1516430)

Crystal data for **16**: $C_{36}H_{24}N_2$, M = 484.57 g/mol, monoclinic, $P2_1/c$, a = 11.7564(2), b = 12.1172(2), c = 17.8498(2) Å, β = 100.1680(10)°, V = 2502.85(7) Å^3, Z = 4, λ(Cu-$K\alpha$) = 1.54184 Å, T = 123 K, colourless plate, Oxford Diffraction Gemini diffractometer; 26306 reflections collected, 4749 independent measured reflections (R_{int} = 0.0268), F^2 refinement, R_1(obs) = 0.0428, wR_2(all data) = 0.1143, 4328 independent observed reflections [|F_o| > 4σ(|F_o|), 2θmax = 139.9°], 343 parameters, GOF = 1.031.

Supplementary X-ray figures and metrics

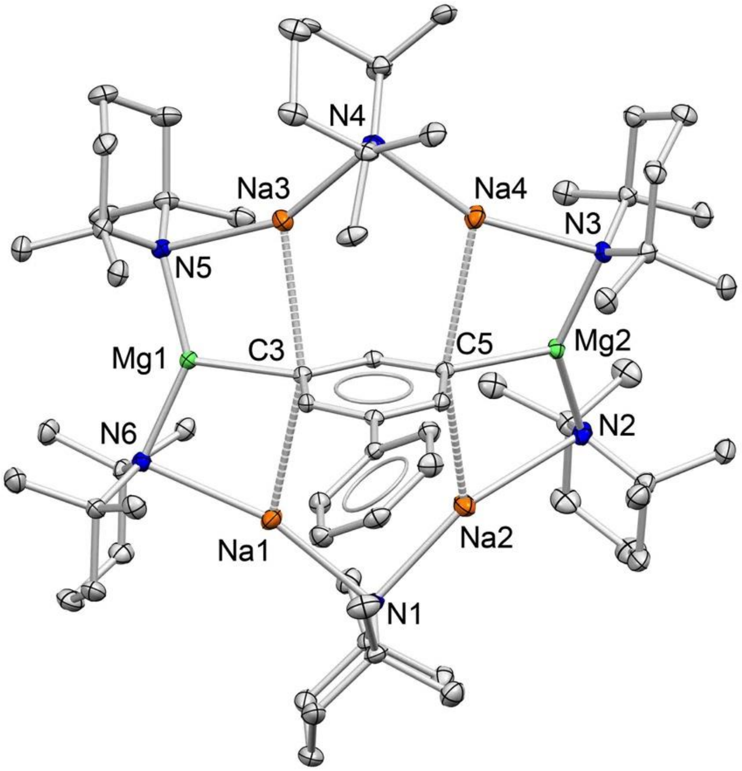

fig. S5. Molecular structure of 2 showing the contents of the asymmetric unit cell. Displacement ellipsoids are displayed at 35% probability. Hydrogen atoms, one disordered methylcyclohexane solvent molecule of crystallization and one disordered component of one TMP ligand have been omitted for clarity. The dashed lines illustrate Na⋯C(aryl) contacts. Selected bond lengths (Å) and angles (º): Mg(1)-C(3) 2.194(2), Mg(2)-C(5) 2.200(2), Mg(1)-N(5) 2.0367(17), Mg(1)-N(6) 2.0457(17), Mg(2)-N(2) 2.0344(17), Mg(2)-N(3) 2.0424(17), Na(1)-C(3) 2.851(2), Na(3)-C(3) 2.805(2), Na(2)-C(5) 2.813(2), Na(4)-C(5) 2.865(2), Na(1)-N(1) 2.3699(18), Na(2)-N(2) 2.5633(19), Na(3)-N(4) 2.3677(18), Na(4)-N(4) 2.3600(18), Na(1)-N(6) 2.5276(18), Na(2)-N(1) 2.3798(19), Na(3)-N(5) 2.5269(18), Na(4)-N(3) 2.5217(17), N(5)-Mg(1)-N(6) 140.14(7), N(5)-Mg(1)-C(3) 108.97(7), N(6)-Mg(1)-C(3) 110.79(7), N(1)-Na(1)-N(6) 158.99(7), N(1)-Na(1)-C(3) 119.12(6), N(6)-Na(1)-C(3) 80.69(6), Na(1)-N(1)-Na(2) 93.29(6), N(2)-Mg(2)-N(3) 138.74(7), N(2)-Mg(2)-C(5) 109.34(7), N(3)-Mg(2)-C(5) 111.91(7), N(1)-Na(2)-N(2) 154.59(7), N(1)-Na(2)-C(5) 124.36(6), N(2)-Na(2)-C(5) 79.84(6), Mg(2)-N(2)-Na(2) 89.87(6), N(4)-Na(3)-N(5) 156.09(7), N(4)-Na(3)-C(3) 123.53(6), N(5)-Na(3)-C(3) 80.30(6), Mg(2)-N(3)-Na(4) 87.73(6), Mg(1)-C(3)-Na(3) 80.14(6), Mg(1)-C(3)-Na(1) 77.63(6), Na(3)-C(3)-Na(1) 157.07(8), N(4)-Na(4)-N(3) 160.67(7), N(4)-Na(4)-C(5) 117.73(6), N(3)-Na(4)-C(5) 81.21(6), Na(4)-N(4)-Na(3) 92.99(6), Mg(1)-N(5)-Na(3) 90.24(6), Mg(2)-C(5)-Na(2) 80.39(6), Mg(2)-C(5)-Na(4) 76.57(6), Na(2)-C(5)-Na(4) 156.16(8), Mg(1)-N(6)-Na(1) 88.26(6). Two distinct types of sodium-nitrogen bonds exist in 2, designated as $Na\text{-}N_{Na}$ and $Na\text{-}N_{Mg}$ bonds (mean distance 2.37 and 2.53 Å, respectively). The remainder of the polymetallic ring consists of shorter Mg-N bond (mean distance 2.04 Å), and the guest biphenyl di-anion is supported by a combination of two Mg-C bonds (mean distance 2.20 Å) and longer Na⋯C(aryl) π-contacts (mean distance 2.83 Å).

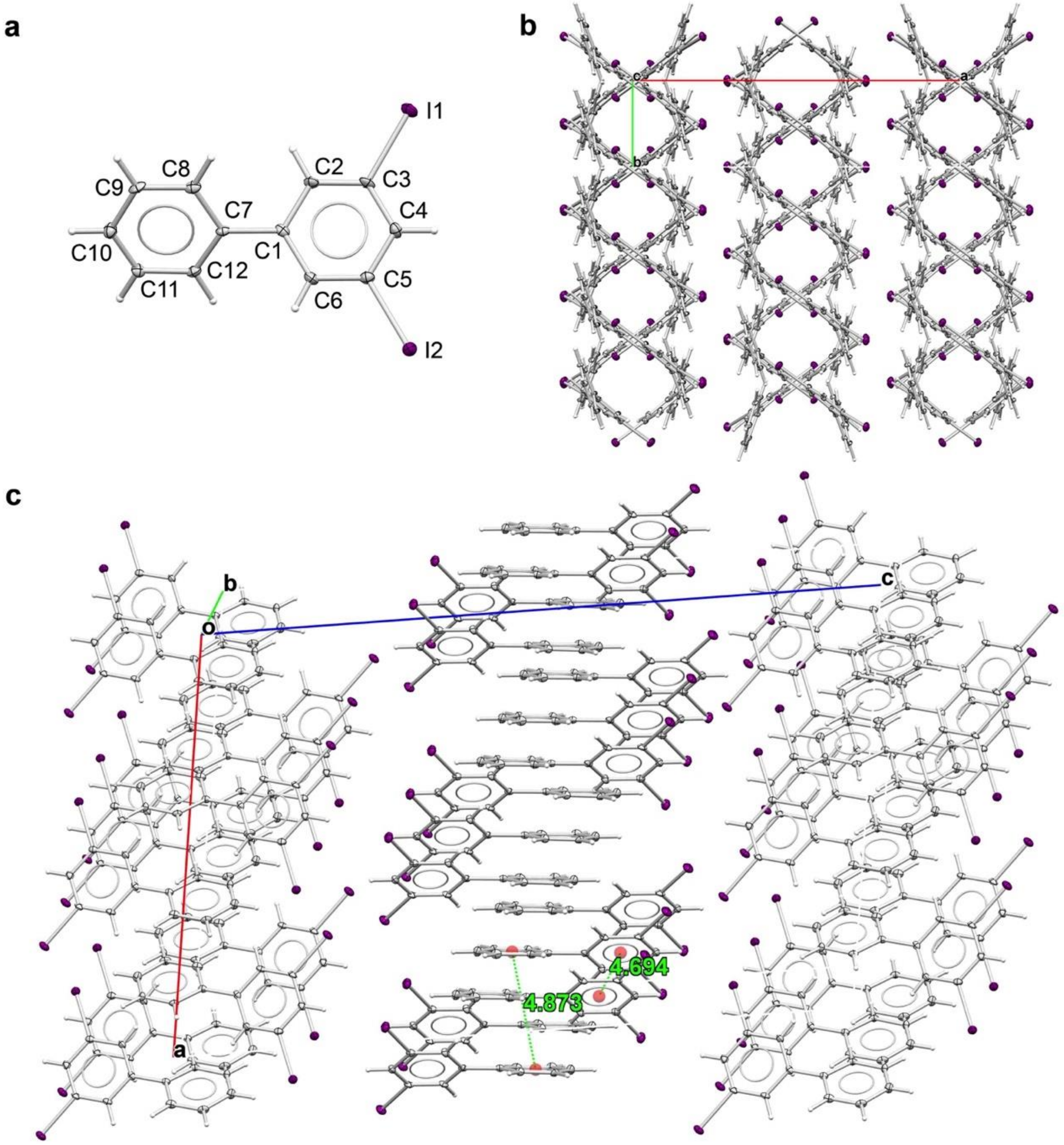

fig. S6. Molecular structure of 3 and its extended packing. (**a**), Molecular structure of **3** showing the contents of the asymmetric unit cell. (**b**), Crystal packing diagram, view along the c-axes. (**c**), Crystal packing diagram showing long π···π stacking interactions [centroid(C_6H_5)····centroid(C_6H_5) and centroid($C_6H_3I_2$)···centroid($C_6H_3I_2$), 4.873 and 4.694 Å, respectively; faced view along the b-axes]. Displacement ellipsoids are displayed at 35% probability. One disordered component of the phenyl C_6H_5 ligand has been omitted for clarity. Selected bond lengths (Å) and angles (º): I(1)-C(3) 2.102(4), I(2)-C(5) 2.092(4), C(1)-C(6) 1.395(6), C(1)-C(2) 1.399(6), C(1)-C(7) 1.478(6), C(2)-C(3) 1.382(7), C(3)-C(4) 1.384(6), C(4)-C(5) 1.383(6), C(5)-C(6) 1.383(7), C(7)-C(12) 1.401(10), C(7)-C(8) 1.412(10), C(10)-C(11) 1.333(10), C(10)-C(9) 1.415(11), C(8)-C(9) 1.382(14), C(11)-C(12) 1.383(13), C(6)-C(1)-C(2) 117.7(4), C(6)-C(1)-C(7) 121.2(4), C(2)-C(1)-C(7) 121.0(4), C(3)-C(2)-C(1) 120.5(4), C(2)-C(3)-C(4) 121.6(4), C(2)-C(3)-I(1) 119.4(3), C(4)-C(3)-I(1) 119.0(4), C(5)-C(4)-C(3) 117.9(5), C(4)-C(5)-C(6) 121.3(4), C(4)-C(5)-I(2) 118.5(4), C(6)-C(5)-I(2) 120.2(3), C(5)-C(6)-C(1) 120.9(4), C(12)-C(7)-C(8) 116.8(6), C(12)-C(7)-C(1) 122.6(5), C(8)-C(7)-C(1) 120.5(6), C(11)-C(10)-C(9) 120.0(7), C(9)-C(8)-C(7) 120.6(8), C(8)-C(9)-C(10) 119.9(8), C(10)-C(11)-C(12) 120.7(8), C(11)-C(12)-C(7) 121.9(7).

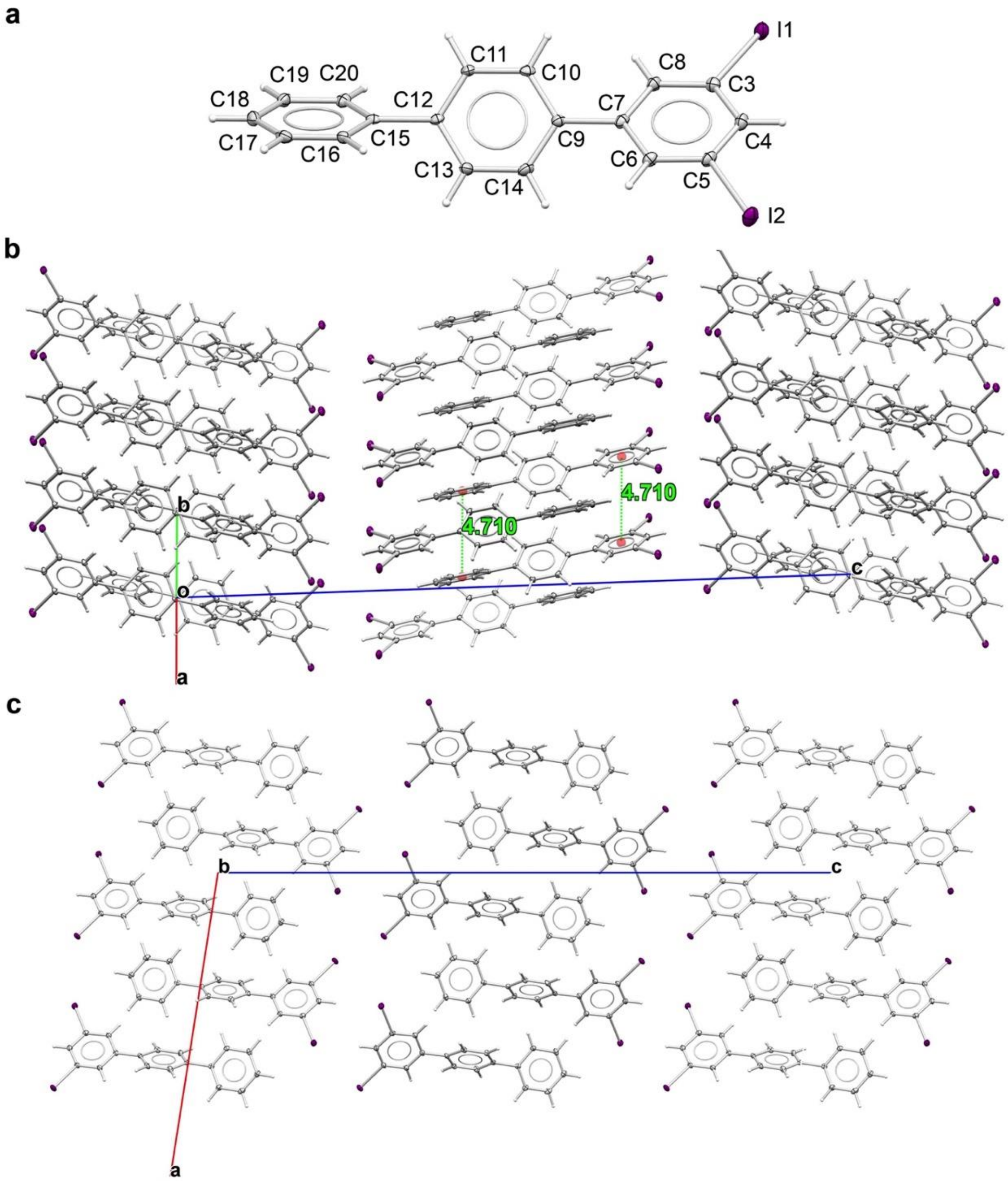

fig. S7. Molecular structure of 5 and its extended packing. (**a**), Molecular structure of **5** showing the contents of the asymmetric unit cell. (**b)**, Crystal packing diagram showing long π···π stacking interactions [centroid(C_6H_5)····centroid(C_6H_5) and centroid($C_6H_3I_2$)···centroid($C_6H_3I_2$), 4.710 Å each; faced view along the b-axes]. (**c)**, Crystal packing diagram, view along the b-axes. Displacement ellipsoids are displayed at 35% probability. One disordered component of the phenyl C_6H_5 and phenylene C_6H_4 groups have been omitted for clarity. Selected bond lengths (Å) and angles (º): I(1)-C(3) 2.097(4), I(2)-C(5) 2.106(4), C(3)-C(8) 1.383(5), C(3)-C(4) 1.391(6), C(4)-C(5) 1.382(6), C(6)-C(5) 1.384(6), C(6)-C(7) 1.398(5), C(7)-C(8) 1.394(6), C(7)-C(9) 1.494(5), C(9)-C(10) 1.403(9), C(9)-C(14) 1.405(10), C(12)-C(11) 1.380(9), C(12)-C(13) 1.388(9), C(12)-C(15) 1.491(5), C(15)-C(16) 1.383(8), C(15)-C(20) 1.398(9), C(18)-C(17) 1.367(10), C(18)-C(19) 1.413(9), C(10)-C(11) 1.392(11), C(13)-C(14) 1.390(11), C(16)-C(17) 1.392(11), C(19)-C(20) 1.393(11), C(8)-C(3)-C(4) 121.7(4), C(8)-C(3)-I(1) 120.0(3), C(4)-C(3)-I(1) 118.2(3), C(5)-C(4)-C(3) 117.6(4), C(5)-C(6)-C(7) 119.5(4), C(4)-C(5)-C(6) 122.2(4), C(4)-C(5)-I(2) 119.1(3), C(6)-C(5)-I(2) 118.7(3), C(8)-C(7)-C(6) 119.1(4), C(8)-

C(7)-C(9) 120.9(3), C(6)-C(7)-C(9) 119.9(4), C(10)-C(9)-C(14) 116.1(5), C(14A)-C(9)-C(7) 119.8(4), C(10A)-C(9)-C(7) 121.5(5), C(10)-C(9)-C(7) 122.1(5), C(14)-C(9)-C(7) 121.7(5), C(3)-C(8)-C(7) 119.9(4), C(11)-C(12)-C(13) 117.9(5), C(11)-C(12)-C(15) 121.5(4), C(13)-C(12)-C(15) 120.5(5), C(16)-C(15)-C(20) 118.9(5), C(16)-C(15)-C(12) 120.4(4), C(20)-C(15)-C(12) 120.7(4), C(17)-C(18)-C(19) 117.4(6), C(11)-C(10)-C(9) 121.5(7), C(12)-C(11)-C(10) 121.5(7), C(12)-C(13)-C(14) 121.0(7), C(13)-C(14)-C(9) 121.9(7), C(15)-C(16)-C(17) 120.2(7), C(18)-C(17)-C(16) 122.5(7), C(20)-C(19)-C(18) 121.0(7), C(19)-C(20)-C(15) 120.1(7).

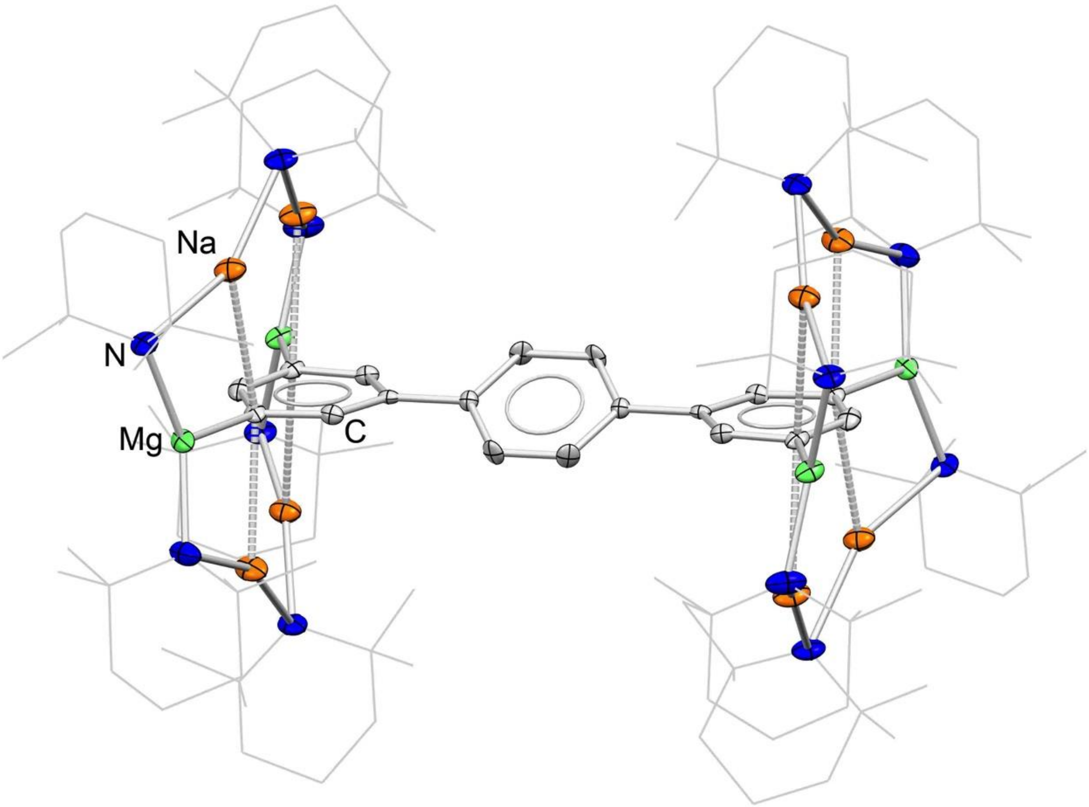

fig. S8. Molecular structure of 6 showing atomic connectivity. Displacement ellipsoids are displayed at 35% probability. One disordered component of one TMP ligand and the phenylene C_6H_4 group have been omitted for clarity. The dashed lines illustrate Na⋯C(aryl) interactions. The TMP C frameworks are pictured as capped thin sticks for clarity.

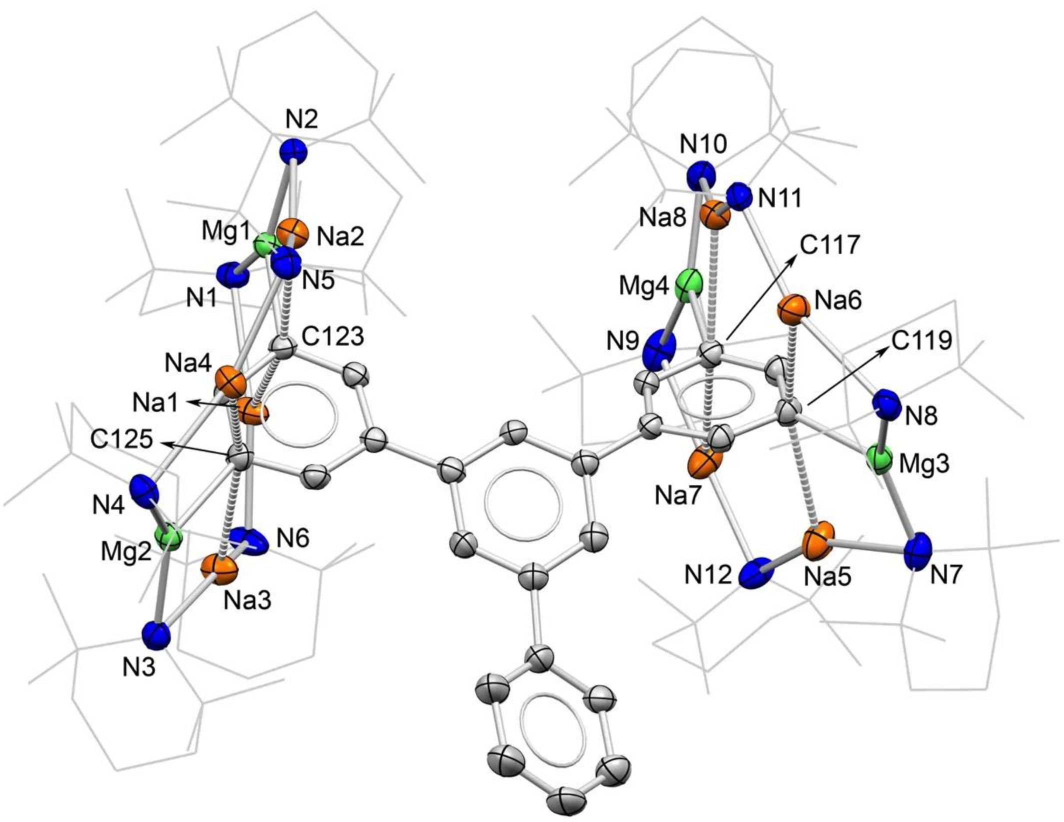

fig. S9. Molecular structure of 12 showing the contents of the asymmetric unit cell. Displacement ellipsoids are displayed at 35% probability. Hydrogen atoms, one disordered methylcyclohexane solvent molecule of crystallization and one disordered component of one TMP ligand have been omitted for clarity. The TMP C framework is pictured as capped thin sticks for clarity. The dashed lines illustrate Na···C(aryl) contacts. Selected bond lengths (Å) and angles (º): Mg(1)-C(123) 2.208(5), Mg(2)-C(125) 2.192(5), Mg(4)-C(117) 2.198(5), Mg(3)-C(119) 2.196(5), Na(1)-C(123) 2.773(5), Na(2)-C(123) 2.797(5), Na(3)-C(125) 2.808(6), Na(4)-C(125) 2.782(6), Na(5)-C(119) 2.830(6), Na(6)-C(119) 2.739(6), Na(7)-C(117) 2.805(5), Na(8)-C(117) 2.755(5), Mg(1)-N(1) 2.034(5), Mg(1)-N(2) 2.037(4), Mg(2)-N(4) 2.033(5), Mg(2)-N(3) 2.055(5), Mg(3)-N(7) 2.052(5), Mg(3)-N(8) 2.055(5), Mg(4)-N(9) 2.043(5), Mg(4)-N(10) 2.015(5), Na(1)-N(1) 2.533(4), Na(1)-N(6) 2.360(5), Na(2)-N(2) 2.524(5), Na(2)-N(5) 2.357(5), Na(3)-N(3) 2.578(4), Na(3)-N(6) 2.349(5), Na(4)-N(4) 2.543(4), Na(4)-N(5) 2.385(5), Na(5)-N(7) 2.509(5), Na(5)-N(12) 2.37(3), Na(6)-N(8) 2.573(5), Na(6)-N(11) 2.33(4), Na(7)-N(9) 2.524(5), Na(7)-N(12) 2.25(3), Na(8)-N(10) 2.598(5), Na(8)-N(11) 2.26(4), N(1)-Mg(1)-N(2) 139.86(18), N(1)-Mg(1)-C(123) 108.85(18), N(2)-Mg(1)-C(123) 111.26(19), N(4)-Mg(2)-N(3) 139.47(18), N(4)-Mg(2)-C(125) 107.85(19), N(3)-Mg(2)-C(125) 112.28(19), N(7)-Mg(3)-N(8) 140.83(19), N(7)-Mg(3)-C(119) 107.8(2), N(8)-Mg(3)-C(119) 110.5(2), N(10)-Mg(4)-N(9) 139.2(2), N(10)-Mg(4)-C(117) 109.5(2), N(9)-Mg(4)-C(117) 111.3(2), N(6)-Na(1)-N(1) 156.45(19), N(6)-Na(1)-C(123) 122.38(17), N(1)-Na(1)-C(123) 81.03(15), N(5)-Na(2)-N(2) 157.39(17), N(5)-Na(2)-C(123) 119.91(16), N(2)-Na(2)-C(123) 82.26(14), N(6)-Na(3)-N(3) 158.32(18), N(6)-Na(3)-C(125) 118.16(16), N(3)-Na(3)-C(125) 81.73(15), N(5)-Na(4)-N(4) 155.79(17), N(5)-Na(4)-C(125) 123.70(16), N(4)-Na(4)-C(125) 79.69(15), N(12)-Na(5)-N(7) 155.4(7), N(12)-Na(5)-C(119) 124.5(6), N(7)-Na(5)-C(119) 79.77(16), N(11)-Na(6)-N(8) 160.1(7), N(11)-Na(6)-C(119) 113.9(8), N(8)-Na(6)-C(119) 82.21(15), N(12)-Na(7)-N(9) 157.3(7), N(12)-Na(7)-C(117) 118.9(8), N(12A)-Na(7)-C(117) 117.4(8), N(9)-Na(7)-C(117) 82.02(16), N(11)-Na(8)-N(10) 155.7(9), N(11)-Na(8)-C(117) 123.7(10), N(10)-Na(8)-C(117) 79.95(15), Mg(1)-N(1)-Na(1) 89.58(17), Mg(1)-N(2)-Na(2) 87.04(15), Mg(2)-N(3)-Na(3) 86.43(15),

Mg(2)-N(4)-Na(4) 90.69(16), Na(2)-N(5)-Na(4) 93.42(16), Na(3)-N(6)-Na(1) 94.77(17), Mg(3)-N(7)-Na(5) 91.64(17), Mg(3)-N(8)-Na(6) 86.31(16), Mg(4)-N(9)-Na(7) 86.97(17), Mg(4)-N(10)-Na(8) 88.71(16), Na(8)-N(11)-Na(6) 99.2(10), Na(7)-N(12)-Na(5) 96.3(9), Mg(4)-C(117)-Na(8) 81.21(16), Mg(4)-C(117)-Na(7) 77.35(16), Na(8)-C(117)-Na(7) 158.2(2), Mg(3)-C(119)-Na(6) 79.64(17), Mg(3)-C(119)-Na(5) 80.62(16), Na(6)-C(119)-Na(5) 160.1(2), Mg(1)-C(123)-Na(1) 80.19(17), Mg(1)-C(123)-Na(2) 77.34(14), Na(1)-C(123)-Na(2) 156.85(19), Mg(2)-C(125)-Na(4) 81.42(17), Mg(2)-C(125)-Na(3) 78.36(16), Na(4)-C(125)-Na(3) 159.74(19).

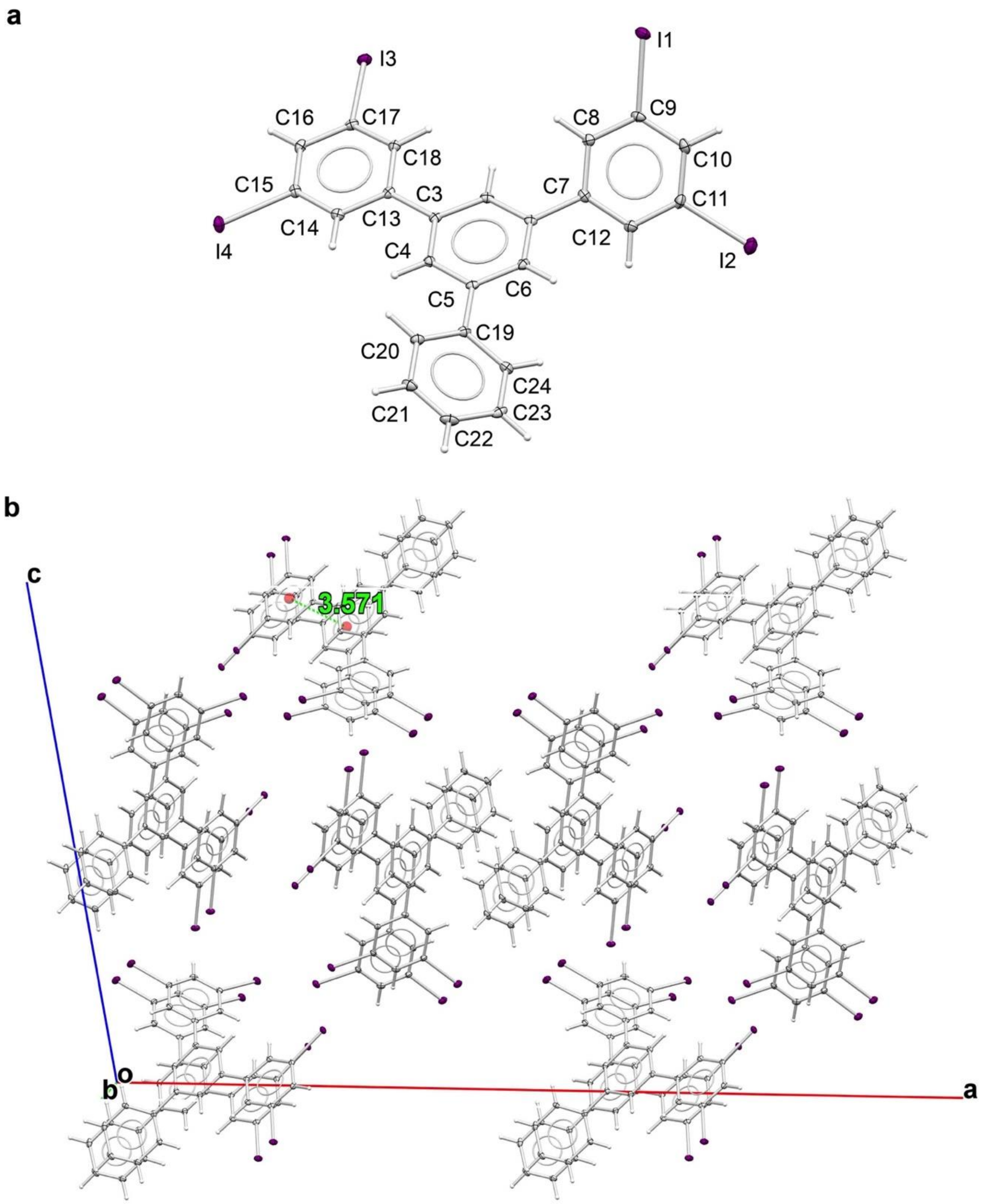

fig. S10. Molecular structure of 13 and its extended packing. (**a**), Molecular structure of **13** showing the contents of the asymmetric unit cell. (**b**), Crystal packing diagram showing π⋯π stacking interactions (centroid-C_6H_3⋯centroid-$C_6H_3I_2$ 3.571 Å, faced view along the b-axes). Displacement ellipsoids are displayed at 35% probability. One disordered component of the phenyl C_6H_5 has been omitted for clarity. Selected bond lengths (Å) and angles (º): I(1)-C(9) 2.101(3), C(1)-C(2) 1.390(4), C(1)-C(6) 1.393(4), C(1)-C(7) 1.489(3), I(2)-C(11) 2.101(3), C(2)-C(3) 1.398(3), I(3)-C(17) 2.102(2), C(3)-C(4) 1.398(3), C(3)-C(13) 1.493(4), I(4)-C(15) 2.101(3), C(4)-C(5) 1.397(4), C(5)-C(6) 1.396(4), C(5)-C(19) 1.491(3), C(7)-C(8) 1.395(3), C(7)-C(12) 1.398(4), C(8)-C(9) 1.387(4), C(9)-C(10) 1.383(4), C(10)-C(11) 1.390(4), C(11)-C(12) 1.383(4), C(13)-C(14) 1.400(3), C(13)-C(18) 1.404(3), C(14)-C(15) 1.377(4), C(15)-C(16) 1.388(4), C(16)-C(17) 1.392(4), C(17)-C(18) 1.381(4), C(19)-C(20) 1.355(6),

C(19)-C(24) 1.405(6), C(22)-C(21) 1.345(7), C(22)-C(23) 1.434(6), C(20)-C(21) 1.405(7), C(23)-C(24) 1.394(7), C(2)-C(1)-C(6) 119.4(2), C(2)-C(1)-C(7) 120.3(2), C(6)-C(1)-C(7) 120.2(2), C(1)-C(2)-C(3) 121.5(2), C(2)-C(3)-C(4) 117.7(2), C(2)-C(3)-C(13) 121.1(2), C(4)-C(3)-C(13) 121.2(2), C(5)-C(4)-C(3) 122.2(2), C(6)-C(5)-C(4) 118.3(2), C(6)-C(5)-C(19) 121.0(2), C(4)-C(5)-C(19) 120.6(2), C(1)-C(6)-C(5) 120.9(2), C(8)-C(7)-C(12) 119.1(2), C(8)-C(7)-C(1) 119.1(2), C(12)-C(7)-C(1) 121.7(2), C(9)-C(8)-C(7) 119.9(2), C(10)-C(9)-C(8) 121.6(2), C(10)-C(9)-I(1) 119.4(2), C(8)-C(9)-I(1) 118.79(19), C(9)-C(10)-C(11) 117.7(2), C(12)-C(11)-C(10) 121.9(2), C(12)-C(11)-I(2) 120.6(2), C(10)-C(11)-I(2) 117.44(19), C(11)-C(12)-C(7) 119.6(2), C(14)-C(13)-C(18) 117.6(2), C(14)-C(13)-C(3) 121.4(2), C(18)-C(13)-C(3) 121.0(2), C(15)-C(14)-C(13) 120.5(2), C(14)-C(15)-C(16) 122.6(2), C(14)-C(15)-I(4) 118.79(18), C(16)-C(15)-I(4) 118.66(19), C(15)-C(16)-C(17) 116.6(2), C(18)-C(17)-C(16) 122.2(2), C(18)-C(17)-I(3) 118.57(18), C(16)-C(17)-I(3) 119.16(19), C(17)-C(18)-C(13) 120.5(2), C(20)-C(19)-C(24) 119.9(4), C(20)-C(19)-C(5) 121.5(3), C(24)-C(19)-C(5) 118.6(3), C(21)-C(22)-C(23) 118.1(4), C(19)-C(20)-C(21) 120.4(5), C(22)-C(21)-C(20) 121.9(5), C(24)-C(23)-C(22) 120.1(5), C(23)-C(24)-C(19) 119.4(4).

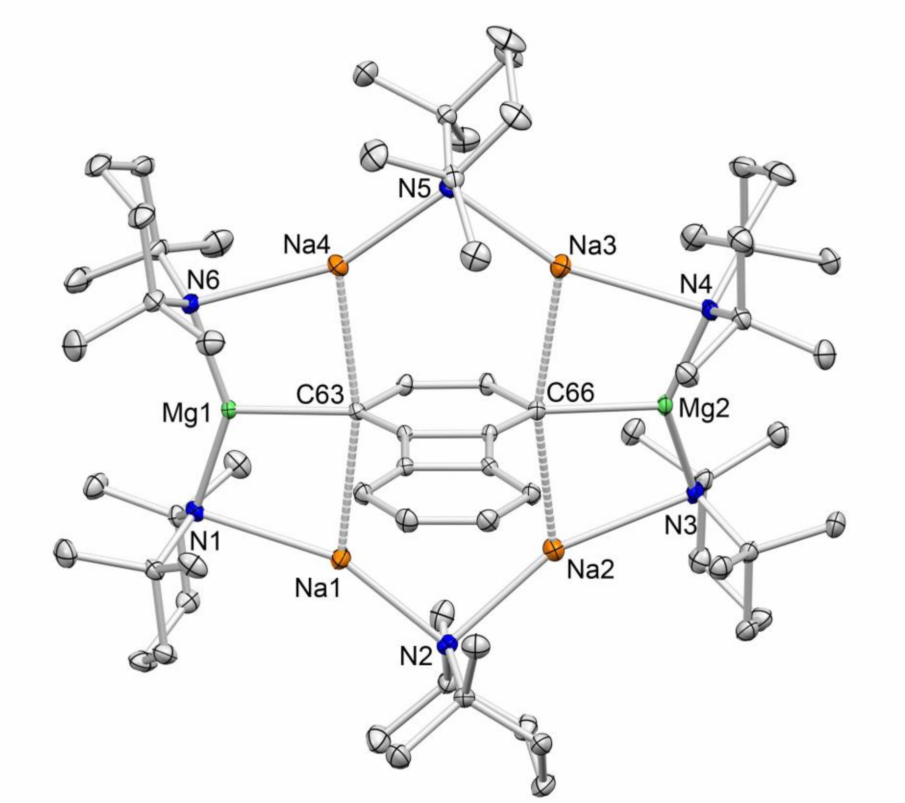

fig. S11. Molecular structure of 14 showing the contents of the asymmetric unit cell. Displacement ellipsoids are displayed at 35% probability. Hydrogen atoms and one disordered component of two TMP ligands have been omitted for clarity. The dashed lines illustrate Na⋯C(aryl) contacts. Selected bond lengths (Å) and angles (º): Mg(1)-C(63) 2.1794(14), Mg(2)-C(66) 2.1938(15), Na(1)-C(63) 2.7011(15), Na(2)-C(66) 2.7224(16), Na(3)-C(66) 2.6644(16), Na(4)-C(63) 2.6614(16), Mg(1)-N(1) 2.0412(13), Mg(1)-N(6) 2.0438(14), Mg(2)-N(3) 2.0427(14), Mg(2)-N(4) 2.0341(14), Na(1)-N(2) 2.3484(14), Na(1)-N(1) 2.6013(14), Na(2)-N(2) 2.4221(14), Na(2)-N(3) 2.6015(14), Na(3)-N(4) 2.6631(14), Na(3)-N(5) 2.3644(14), Na(4)-N(5) 2.3843(14), Na(4)-N(6) 2.6165(14), N(1)-Mg(1)-N(6) 144.56(5), N(1)-Mg(1)-C(63) 108.00(5), N(6)-Mg(1)-C(63) 107.36(5), N(2)-Na(1)-N(1) 156.60(5), N(2)-Na(1)-C(63) 123.15(5), N(1)-Na(1)-C(63) 80.18(4), Mg(1)-N(1)-Na(1) 88.35(5), N(4)-Mg(2)-N(3) 142.00(6), N(4)-Mg(2)-C(66) 109.52(6), N(3)-Mg(2)-C(66) 108.15(6), N(2)-Na(2)-N(3) 159.17(5), N(2)-Na(2)-C(66) 119.79(5), N(3)-Na(2)-C(66) 80.24(5), Na(1)-N(2)-Na(2) 99.19(5), N(5)-Na(3)-N(4) 160.38(5), N(5)-Na(3)-C(66) 118.77(5), N(4)-Na(3)-C(66) 80.84(4), Mg(2)-N(3)-Na(2) 87.96(5), N(5)-Na(4)-N(6) 159.36(5), N(5)-Na(4)-C(63) 120.32(5), N(6)-Na(4)-C(63) 80.31(4), Mg(1)-Na(4)-Na(3) 124.69(2), Mg(2)-N(4)-Na(3) 85.55(5), Mg(1)-C(63)-Na(4) 84.17(5), Mg(1)-C(63)-Na(1) 83.08(5), Na(4)-C(63)-Na(1) 167.26(6), Mg(2)-C(66)-Na(3) 82.52(5), Mg(2)-C(66)-Na(2) 82.00(5), Na(3)-C(66)-Na(2) 163.69(6), Na(3)-N(5)-Na(4) 105.35(5), Mg(1)-N(6)-Na(4) 88.03(5).

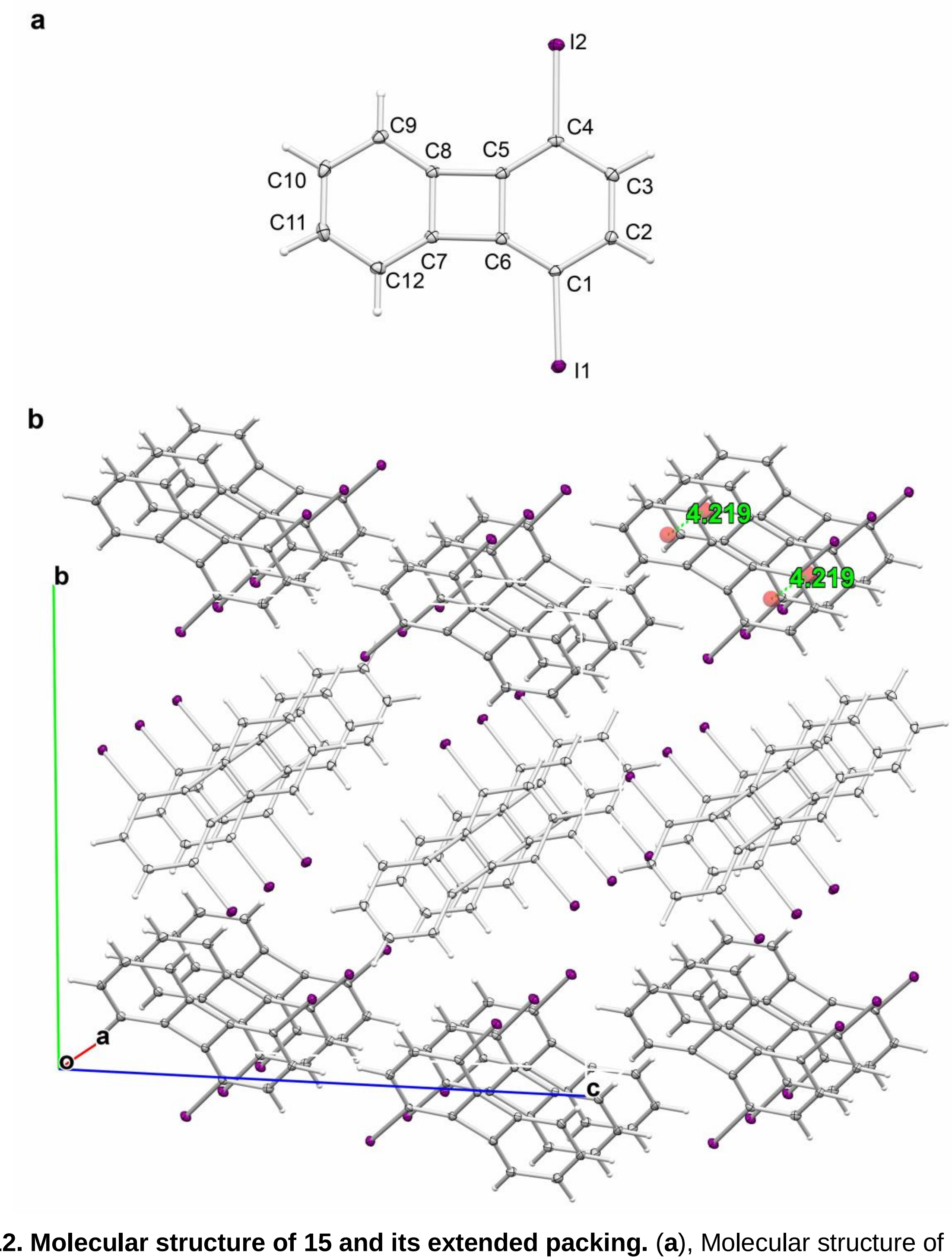

fig. S12. Molecular structure of 15 and its extended packing. (**a**), Molecular structure of **15** showing the contents of the asymmetric unit cell. (**b**), Crystal packing diagram showing π⋯π stacking interactions (centroid-C_6H_4⋯·centroid-C_6H_4, centroid-$C_6H_2I_2$⋯·centroid- $C_6H_2I_2$ 4.219 Å, faced view along the a-axes). Displacement ellipsoids are displayed at 35% probability. Selected bond lengths (Å) and angles (º): I(1)-C(1) 2.091(3), C(1)-C(6) 1.362(4), C(1)-C(2) 1.407(4), C(2)-C(3) 1.385(4), I(2)-C(4) 2.085(3), C(4)-C(5) 1.357(4), C(4)-C(3) 1.411(4), C(5)-C(6) 1.427(4), C(5)-C(8) 1.501(4), C(6)-C(7) 1.506(4), C(8)-C(9) 1.365(4), C(8)-C(7) 1.417(4), C(7)-C(12) 1.365(4), C(9)-C(10) 1.404(4), C(10)-C(11) 1.378(4), C(11)-C(12) 1.421(4), C(6)-C(1)-C(2) 117.2(3), C(6)-C(1)-I(1) 122.9(2), C(2)-C(1)-I(1) 119.9(2), C(3)-C(2)-C(1) 121.8(3), C(5)-C(4)-C(3) 116.8(3), C(5)-C(4)-I(2) 122.1(2), C(3)-C(4)-I(2) 121.0(2), C(2)-C(3)-C(4) 120.9(3), C(4)-C(5)-C(6) 122.5(3), C(4)-C(5)-C(8) 147.6(3), C(6)-C(5)-C(8)

89.9(2), C(1)-C(6)-C(5) 120.7(3), C(1)-C(6)-C(7) 149.5(3), C(5)-C(6)-C(7) 89.7(2), C(9)-C(8)-C(7) 122.7(3), C(9)-C(8)-C(5) 146.9(3), C(7)-C(8)-C(5) 90.3(2), C(12)-C(7)-C(8) 122.3(3), C(12)-C(7)-C(6) 147.6(3), C(8)-C(7)-C(6) 90.0(2), C(8)-C(9)-C(10) 115.3(3), C(11)-C(10)-C(9) 122.4(3), C(10)-C(11)-C(12) 122.1(3), C(7)-C(12)-C(11) 115.1(3).

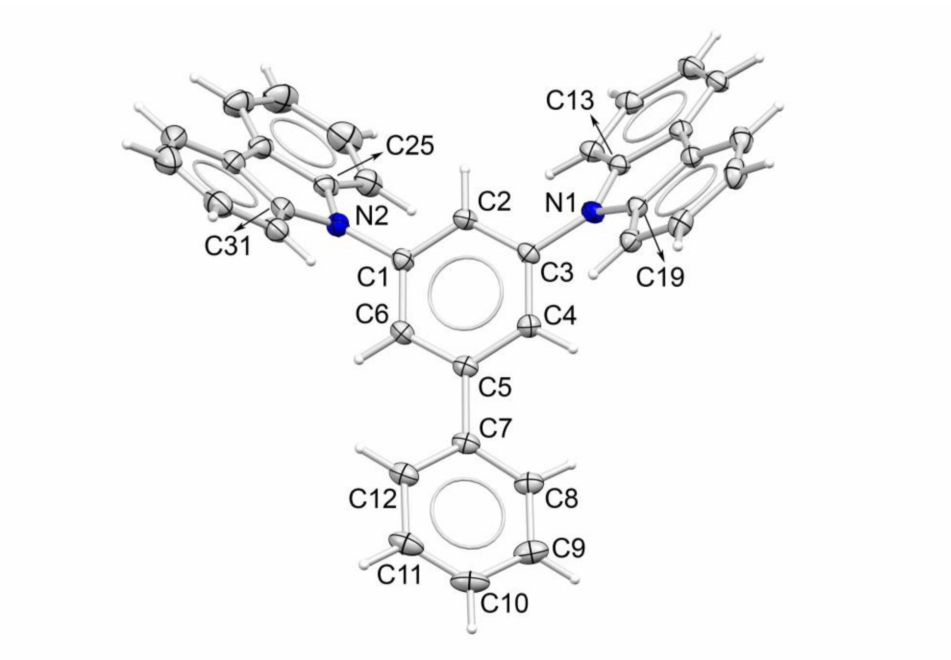

fig. S13. Molecular structure of 16 showing the contents of the asymmetric unit cell. (**a**), Molecular structure of **16** showing the contents of the asymmetric unit cell. Displacement ellipsoids are displayed at 35% probability. Selected bond lengths (Å) and angles (º): N(1)-C(13) 1.3949(17), N(1)-C(19) 1.3979(17), N(1)-C(3) 1.4220(17), C(1)-C(6) 1.3845(19), C(1)-C(2) 1.3897(19), C(1)-N(2) 1.4225(17), N(2)-C(25) 1.3879(18), N(2)-C(31) 1.3975(19), C(2)-C(3) 1.3857(19), C(3)-C(4) 1.3888(18), C(4)-C(5) 1.4009(19), C(5)-C(6) 1.3955(19), C(5)-C(7) 1.4888(18), C(7)-C(8) 1.395(2), C(7)-C(12) 1.400(2), C(8)-C(9) 1.386(2), C(9)-C(10) 1.381(3), C(10)-C(11) 1.383(3), C(11)-C(12) 1.390(2), C(13)-N(1)-C(19) 108.50(11), C(13)-N(1)-C(3) 125.61(11), C(19)-N(1)-C(3) 125.61(11), C(25)-N(2)-C(31) 108.76(12), C(25)-N(2)-C(1) 125.45(12), C(31)-N(2)-C(1) 124.04(12).

Supplementary text

Reactivity studies of biphenyl and NaTMP

Control reactions were conducted between biphenyl and NaTMP (1 and 2 equiv) under the same reaction conditions that yields **2** (methylcyclohexane, 65 °C, 16 h). The corresponding reaction mixtures were analysed by ^{1}H NMR spectroscopy in [D_{12}]cyclohexane showing no reaction between NaTMP and biphenyl when 1 and 2 equiv of NaTMP were used. No evidence of either *ortho*-metalation or 3,5-dimetalation of biphenyl were observed by ^{1}H NMR spectroscopy under these reaction conditions (see fig. S14).

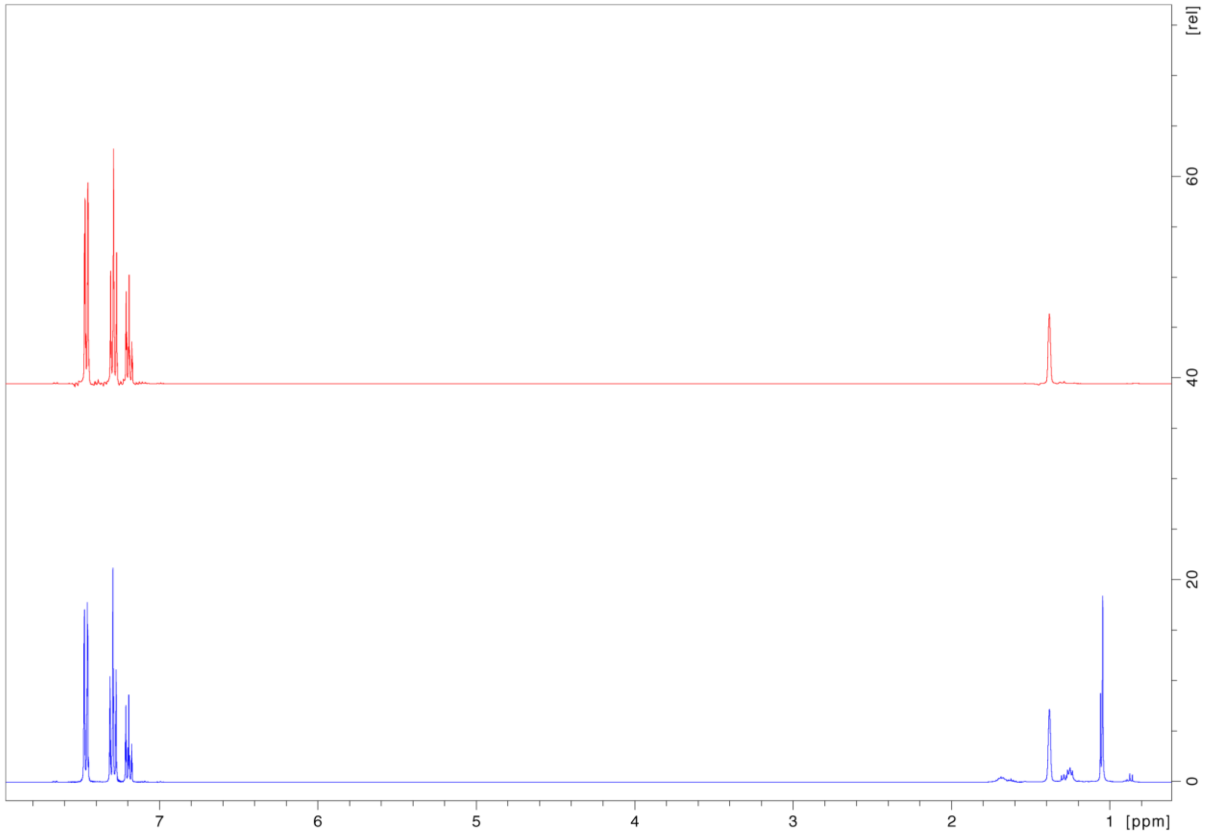

fig. S14. ^{1}H NMR study (400.1 MHz; [D_{12}]cyclohexane, 300 K) of biphenyl (top; red) and a control reaction of biphenyl and NaTMP in a 1:2 M ratio in methylcyclohexane after 24 hours at 65°C (bottom; blue). The reaction was monitored by ^{1}H NMR spectroscopic analyses of *in situ* aliquots. Under these reaction conditions, no metalation reaction was observed. Present within the *in situ* reaction mixture aliquots in [D_{12}]cyclohexane solution also small amounts of TMP(H) were present which is presumably a result of unavoidable hydrolysis of NaTMP.

Reactivity studies of biphenyl and TMPMg(^{n}Bu)

Control reactions were conducted between biphenyl and TMPMg(^{n}Bu) (1 and 2 equiv) under the same reaction conditions that yields **2** (methylcyclohexane, 65 °C, 16 h). The corresponding reaction mixtures were analysed by ^{1}H NMR spectroscopy in [D_{12}]cyclohexane showing no reaction between TMPMg(^{n}Bu) and biphenyl when 1 and 2 equiv of TMPMg(^{n}Bu) were used. No evidence of either *ortho*-metalation or 3,5-dimetalation of biphenyl were observed by ^{1}H NMR spectroscopy under these reaction conditions (see fig. S15).

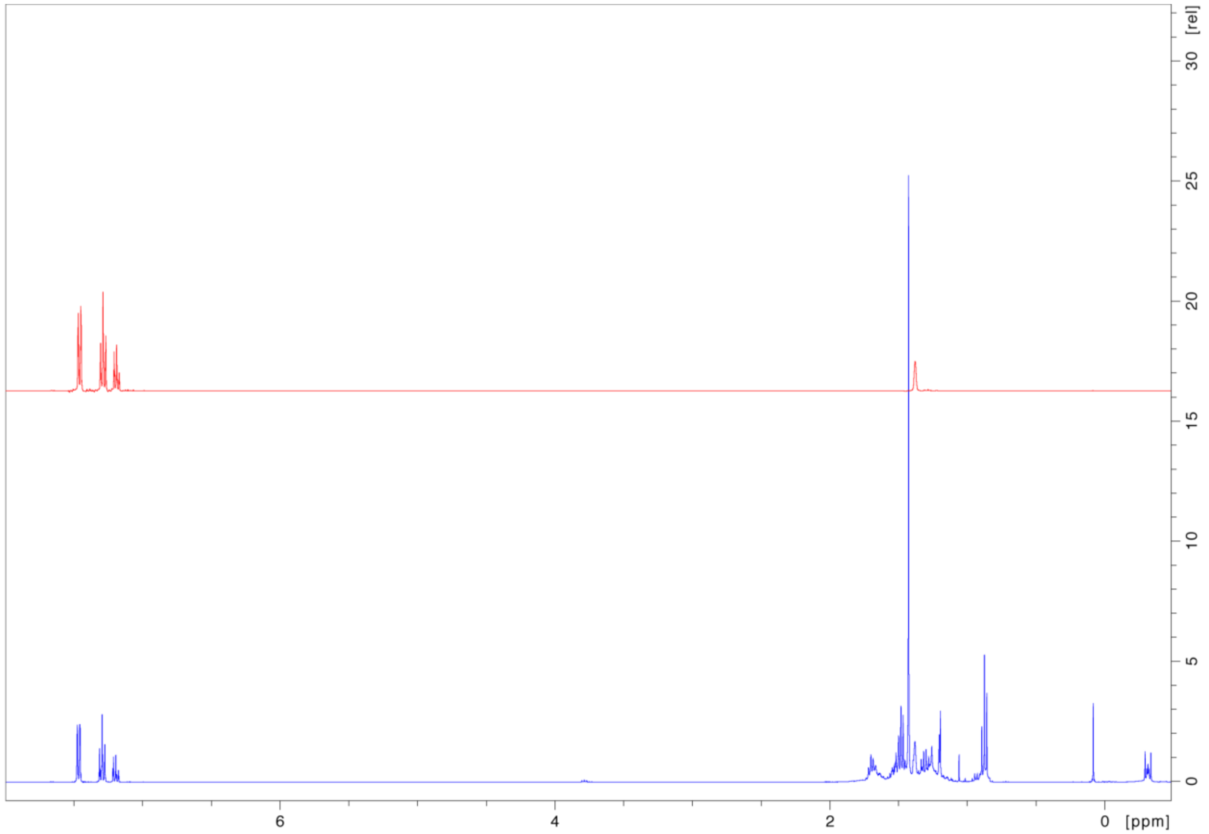

fig. S15. ^{1}H NMR study (400.1 MHz; [D_{12}]cyclohexane, 300 K) of biphenyl (top; red) and a control reaction of biphenyl and ^{n}BuMgTMP in a 1:2 M ratio in methylcyclohexane after 16 hours at 65°C (bottom; blue). The reaction was monitored by 1H NMR spectroscopic analyses of *in situ* aliquots. Under these reaction conditions no metalation reaction was observed. Present within the *in situ* reaction mixture aliquots in [D_{12}]cyclohexane solution also small amounts of TMP(H) were present which is presumably a result of unavoidable hydrolysis of *n*BuMgTMP.

Reactivity study of biphenyl and the metalating reagent [$Na_4Mg_2(TMP)_6(^nBu)_2$] (1)

Biphenyl was reacted with and equimolar amount and excess (2 equiv) of **1** at 65 °C for 16 h. Aliquots of the corresponding reaction mixtures were analyzed by ^{1}H NMR spectroscopy in [D_{12}]cyclohexane showing consumption of biphenyl and **1** by an equivalent increasing of the amount of the

organometallic derivative **2** in the reaction mixtures. In all the cases, the organometallic compound **2** was the main component present in the *in situ* reaction mixtures. This was confirmed by ^{1}H NMR analyses of the two representative singlets pattern at 7.92 and 8.05 ppm in a 2:1 ratio, corresponding to the remaining aromatic protons in the 3,5-dimetalated phenyl 3,5-Mg_2-C_6H_3 ring, and a set of resonances for the corresponding protons in the non metalated phenyl ring observed in the range 7.15-7.39 ppm (see fig. S16 for a ^{1}H NMR spectroscopic study on the aforementioned *in situ* reaction mixtures). Additionally, the organometallic derivative **2** was successfully isolated in 61% yield when biphenyl was reacted with 2 equivalents of **1** at 65 °C for 16 h after cooling down the reaction mixture and room temperature for 24 h. Subsequent iodination of the corresponding *in situ* reaction mixture resulted in isolation of 3,5-diiodobiphenyl **3** in 52% (isolated yield).

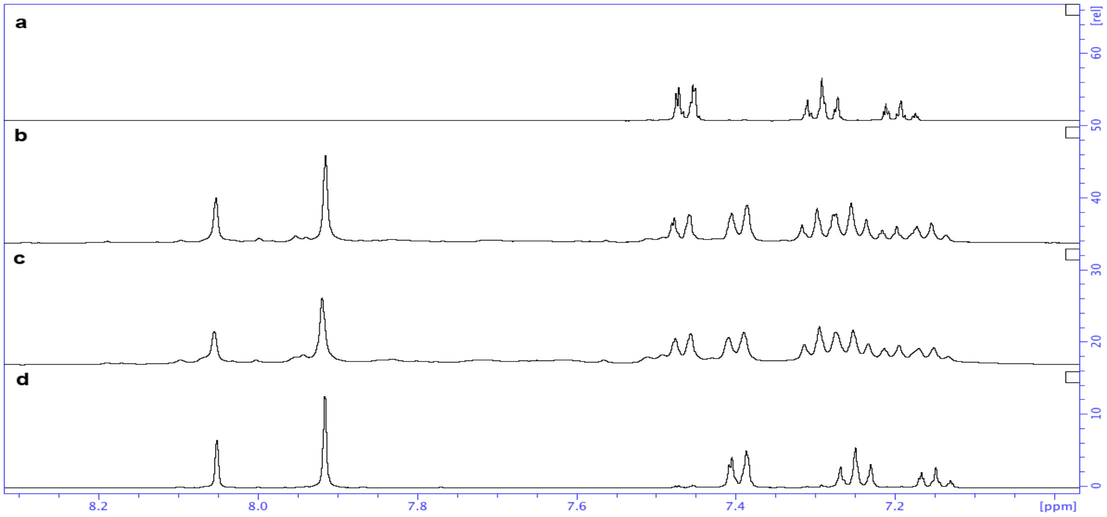

fig. S16. Representative example of 3,5-dimetalation of biphenyl to give 2. ^{1}H NMR (400.1 MHz, [D_{12}]cyclohexane, 300 K) spectrum of (**a**) biphenyl; (**b**) *in situ* reaction mixture of equimolar amounts of biphenyl and **1** in methylcyclohexane after 16 h at 65 °C; (**c**) *in situ* reaction mixture of biphenyl and **1** in a 1:2 molar ratio in methylcyclohexane after 16 h at 65ºC; and (**d**) isolated organometallic compound **2**. The reactions of biphenyl and **1** in equimolar amounts (b) and in a 1:2 molar ratios (c) at 65 °C were monitored by ^{1}H NMR spectroscopic analyses of *in situ* aliquots. After 16 h the metalation of biphenyl to give **2** as the main organometallic species in the reaction mixture was observed in both cases. Small amounts of TMP(H) and biphenyl were also present which is presumably as a result of unavoidable hydrolysis of **2**. The highly reactive nature of **2** and **1** with commonly used internal standards, and the low solubility of **2** in [D_{12}]cyclohexane hampered the determination of *in situ* conversions of biphenyl to **2** under these reactions conditions (b and c).

NMR spectra

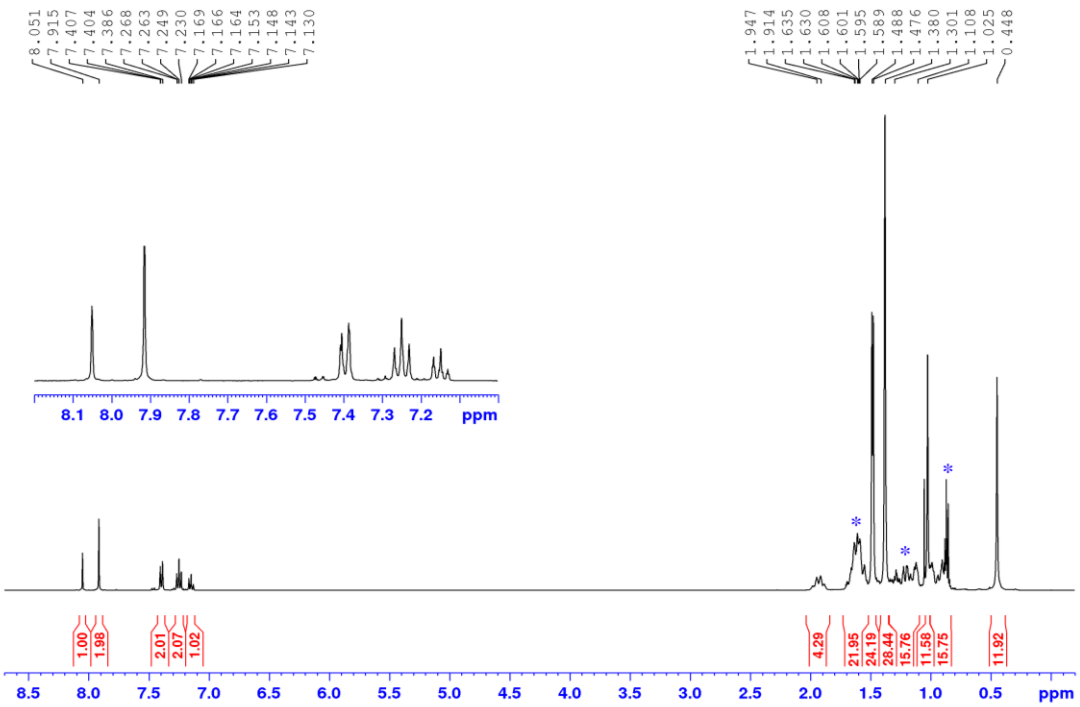

fig. S17. ^{1}H NMR (400.1 MHz; [D_{12}]cyclohexane, 300 K) spectrum of 2. Residual signals for methylcylohexane (C_7H_{14}, solvent of crystallization denoted with blue asterisks) are also observed.

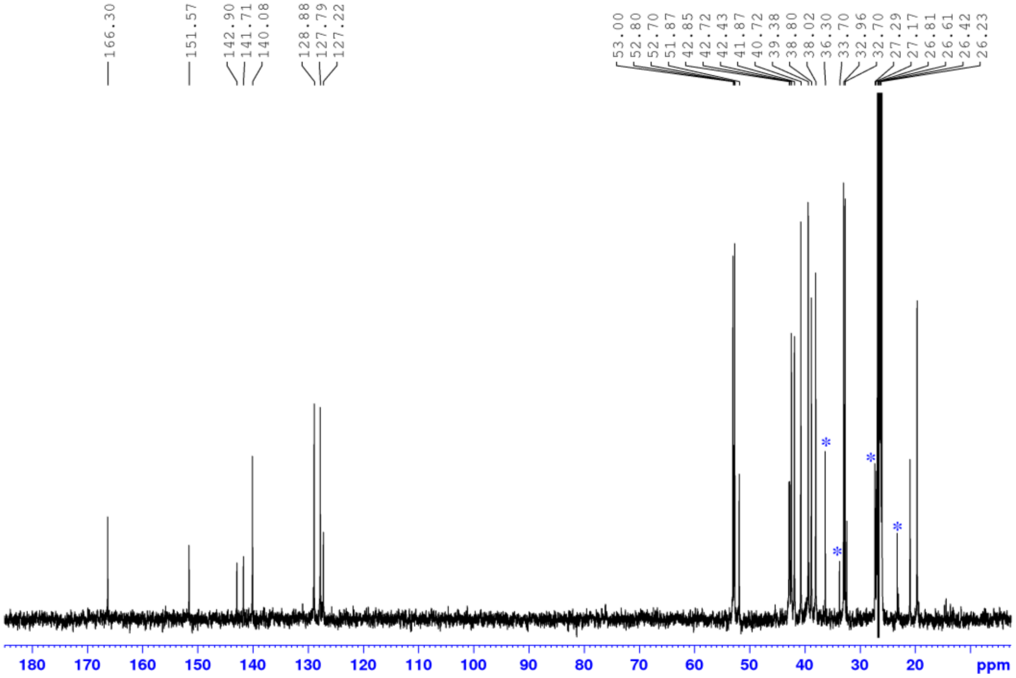

fig. S18. ^{13}C{^{1}H} NMR (100.6 MHz; [D_{12}]cyclohexane, 300 K) spectrum of 2. Residual signals for methylcylohexane (C_7H_{14}, solvent of crystallization denoted with blue asterisks) are also observed.

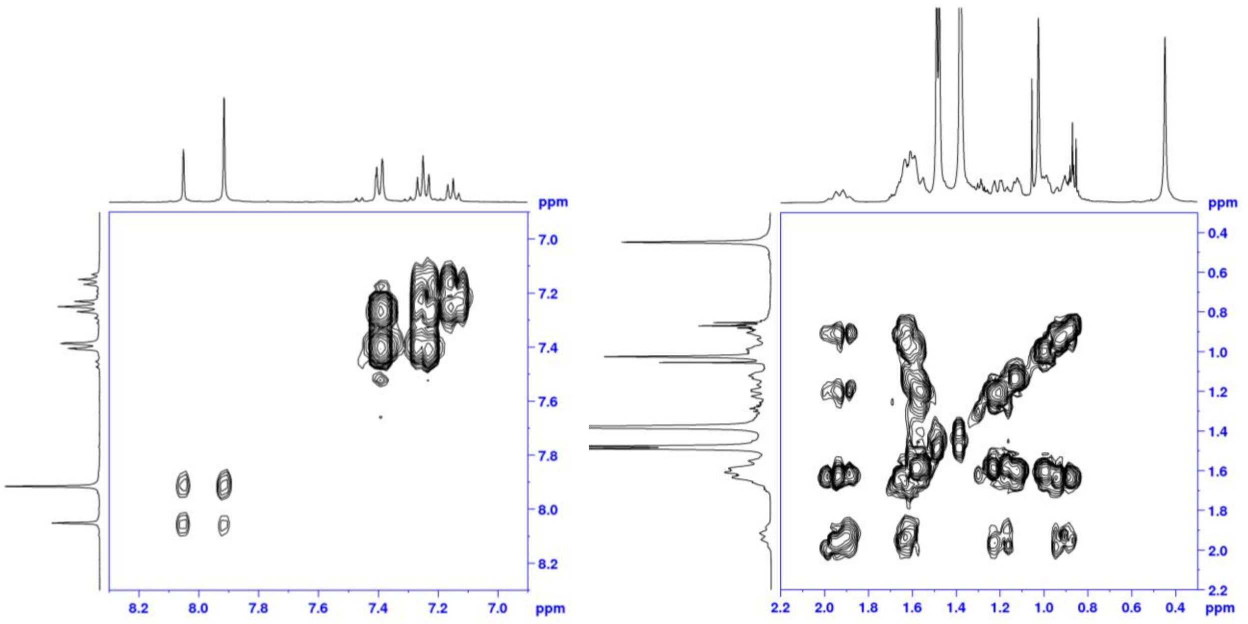

fig. S19. Sections of the $^{1}H,^{1}H$-COSY NMR (400.1 MHz; [D_{12}]cyclohexane, 300 K) spectrum of 2.

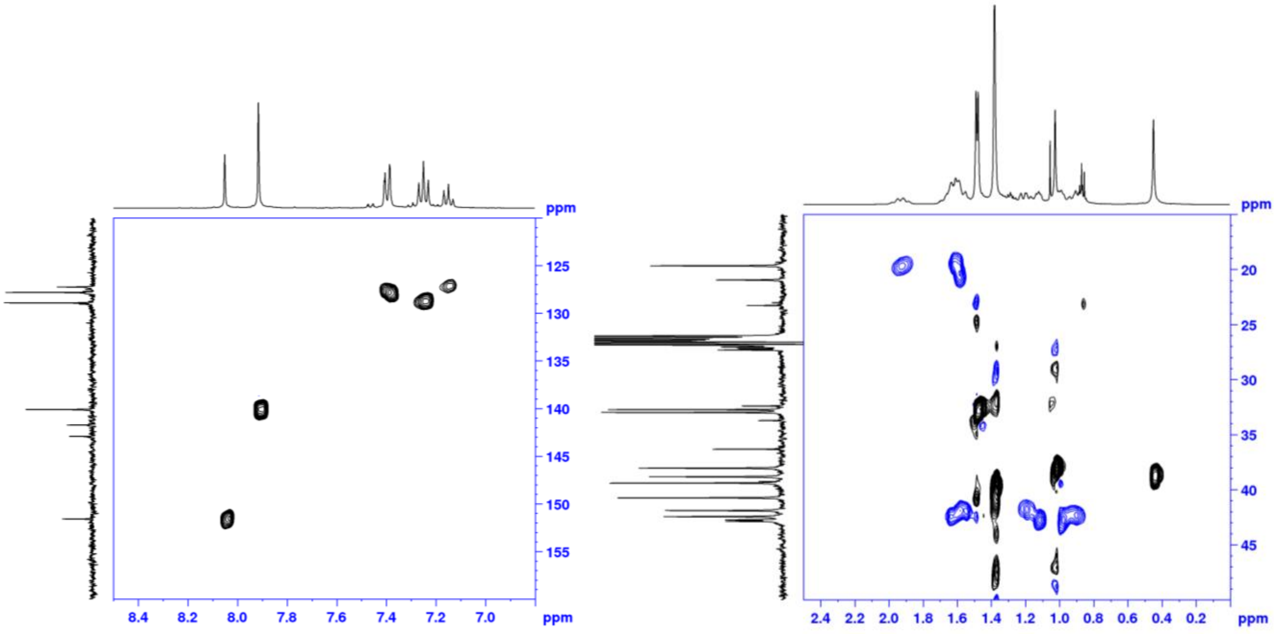

fig. S20. Sections of the phase-sensitive $^{1}H,^{13}C$-HSQC NMR (400.1 MHz; [D_{12}]cyclohexane, 300 K) spectrum of 2. Cross-peaks corresponding to CH_3 and CH groups are pictured in black whilst they are blue for CH_2 groups.

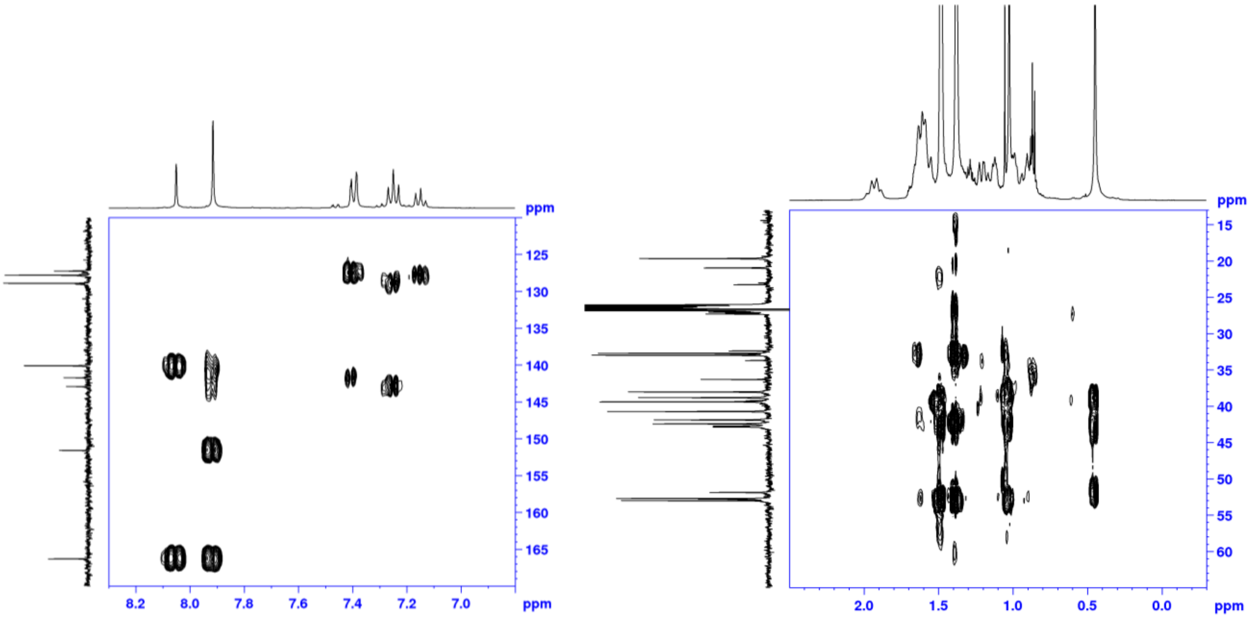

fig. S21. Sections of the ^{1}H,^{13}C-HMBC NMR (400.1 MHz; [D_{12}]cyclohexane, 300 K) spectrum of 2.

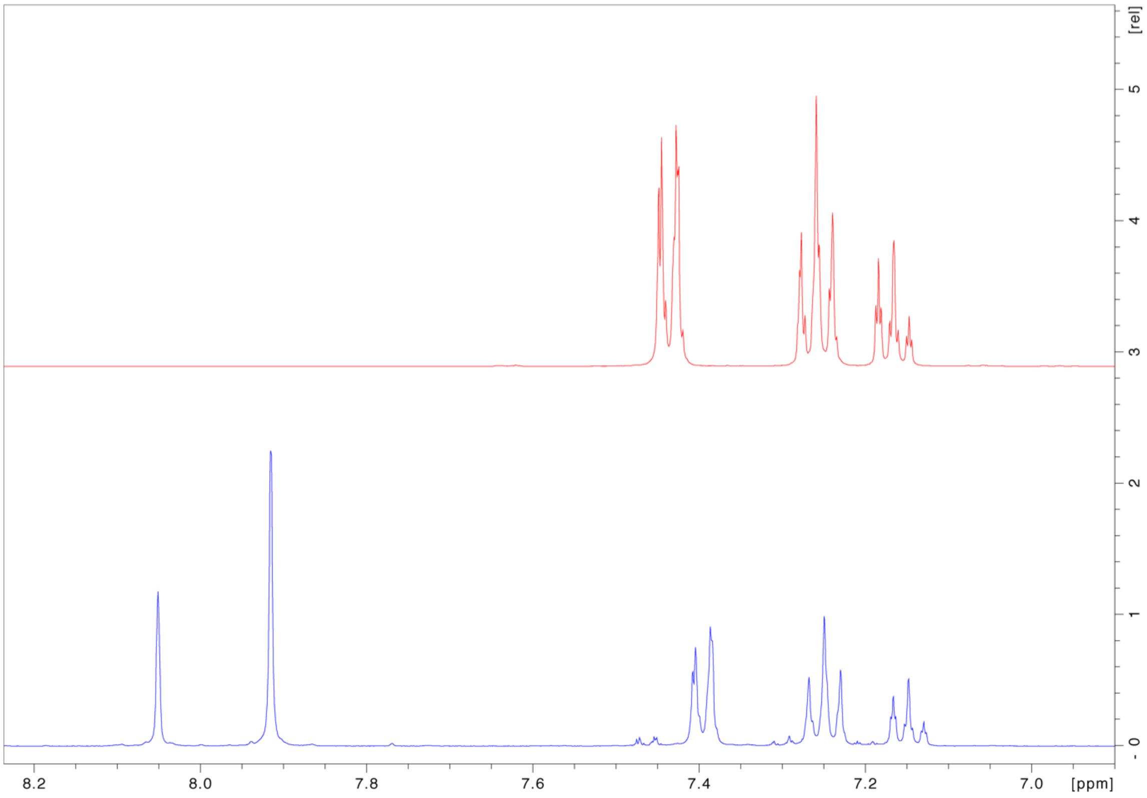

fig. S22. Sections of the ^{1}H NMR (400.1 MHz; [D_{12}]cyclohexane, 300 K) spectra of biphenyl (top; red) and 2 (bottom; blue) showing the aromatic resonances. Residual signals for biphenyl are observed in the ^{1}H NMR spectrum of **2** in [D_{12}]cyclohexane solution due to unavoidable hydrolysis of the NMR sample.

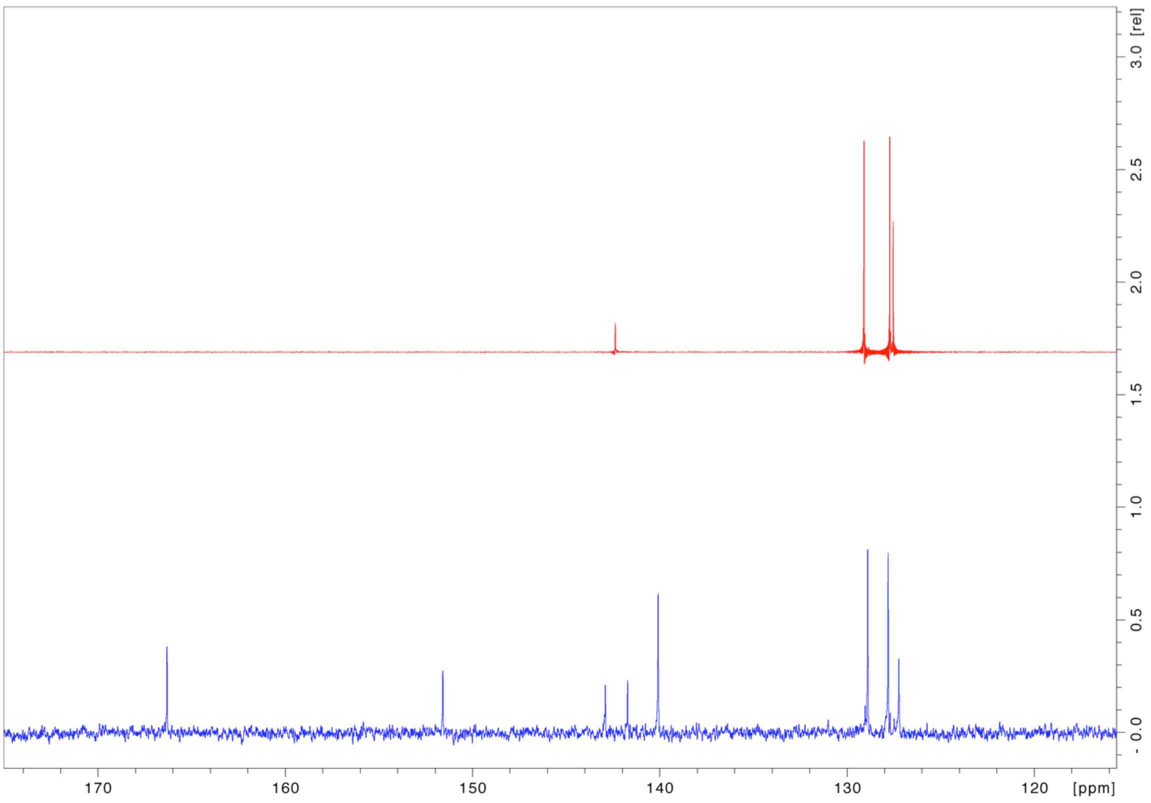

fig. S23. Sections of the $^{13}C\{^{1}H\}$ NMR (100.6 MHz; [D_{12}]cyclohexane, 300 K) spectra of biphenyl (top; red) and 2 (bottom; blue) showing the aromatic resonances.

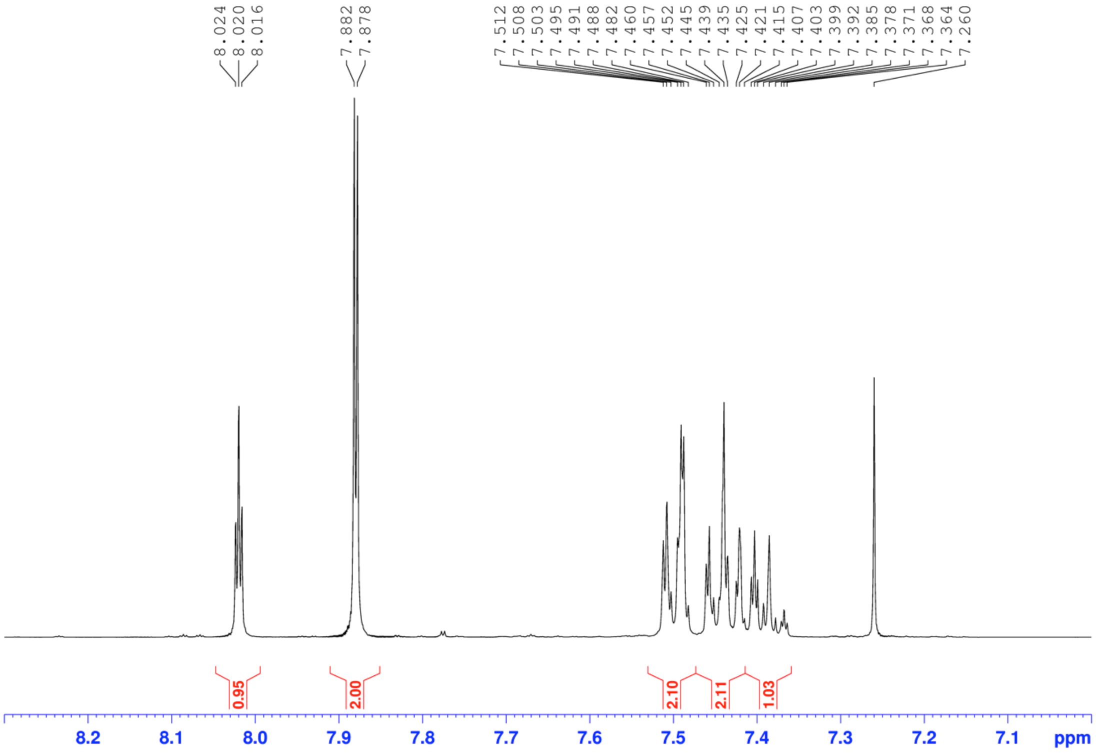

fig. S24. ^{1}H NMR (400.1 MHz; $CDCl_3$, 300 K) spectrum of 3.

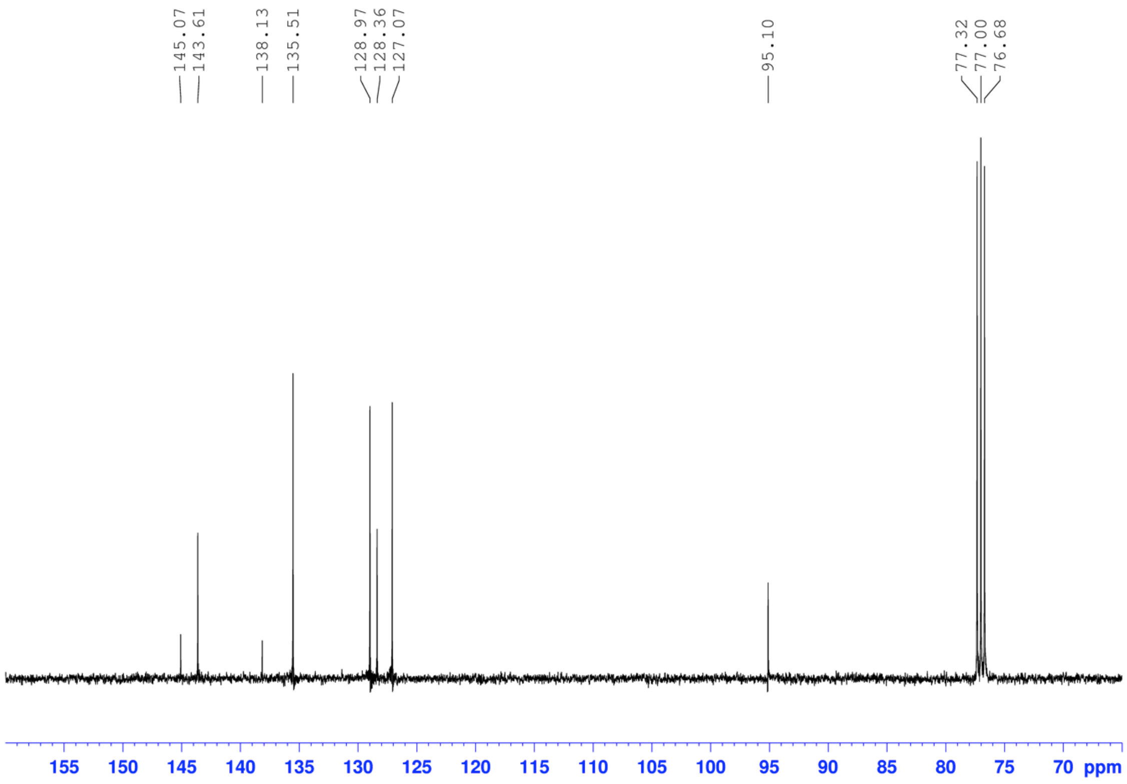

fig. S25. $^{13}C\{^{1}H\}$ **NMR (100.6 MHz; $CDCl_3$, 300 K) spectrum of 3.**

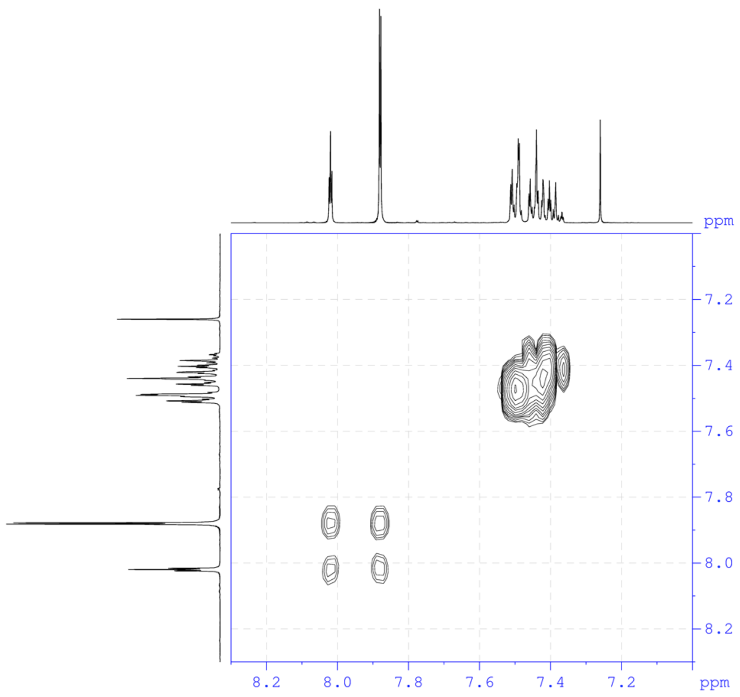

fig. S26. $^{1}H,^{1}H$**-COSY NMR (400.1 MHz; $CDCl_3$, 300 K) spectrum of 3.**

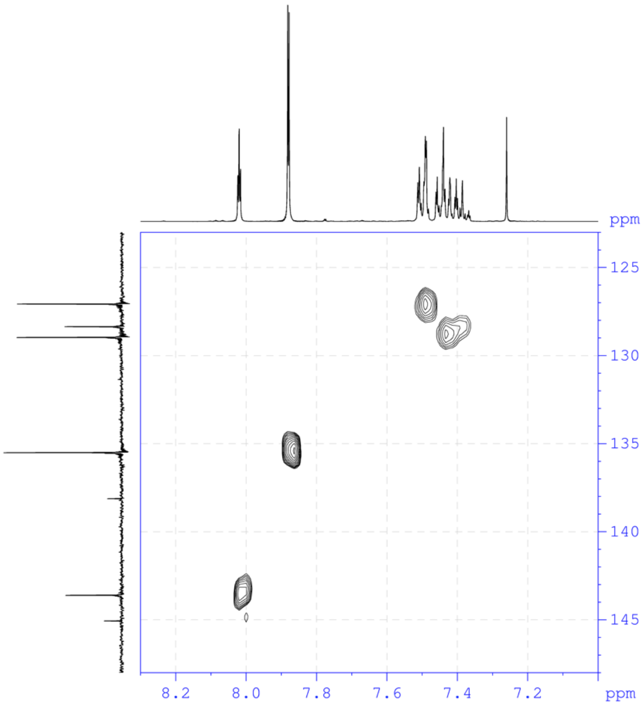

fig. S27. **$^{1}H,^{13}C$-HSQC NMR (400.1 MHz; $CDCl_3$, 300 K) spectrum of 3.**

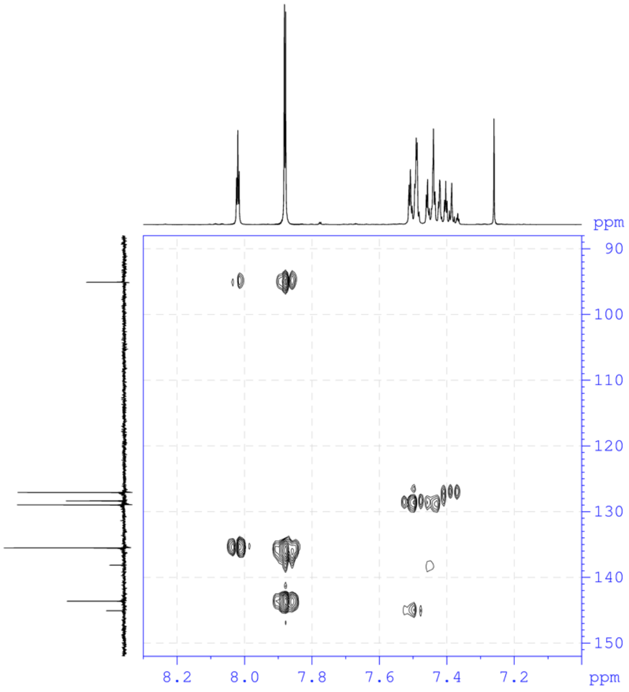

fig. S28. **$^{1}H,^{13}C$-HMBC NMR (400.1 MHz; $CDCl_3$, 300 K) spectrum of 3.**

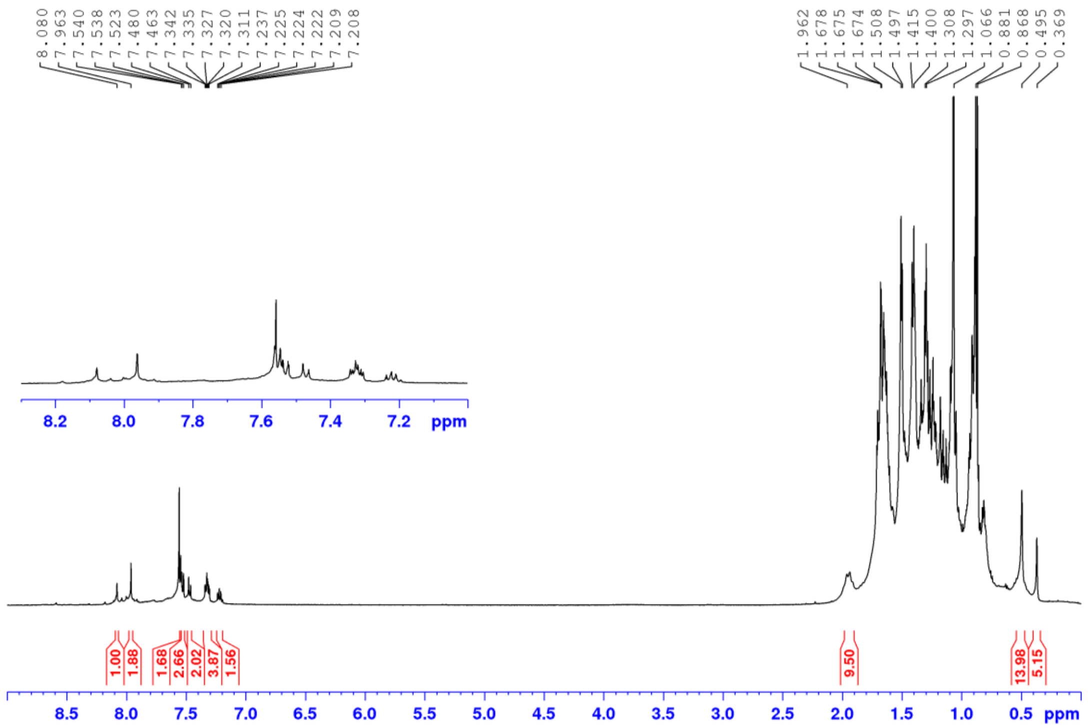

fig. S29. ^{1}H NMR (400.1 MHz; [D_{12}]cyclohexane, 300 K) spectrum of an in situ sample of 4. Residual signals corresponding to *para*-terphenyl and TMP(H) are present within the [D_{12}]cyclohexane solution of **4** due to unavoidable hydrolysis.

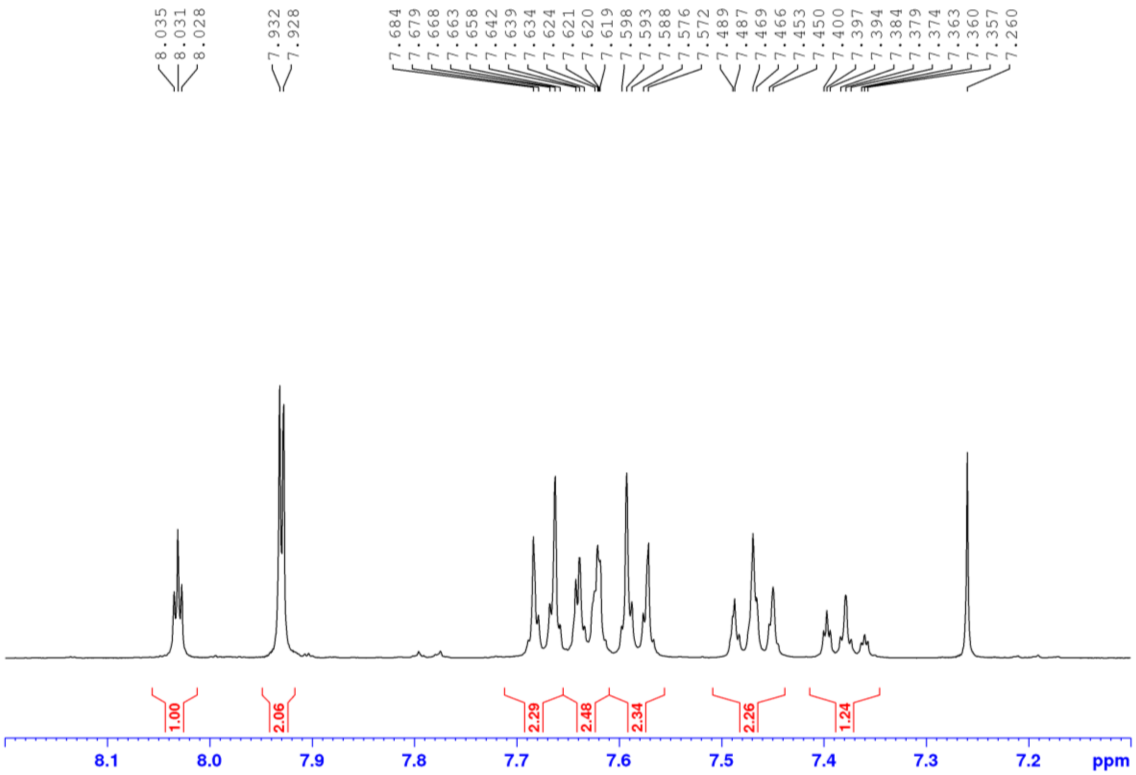

fig. S30. ^{1}H NMR (400.1 MHz; $CDCl_3$, 300 K) spectrum of 5.

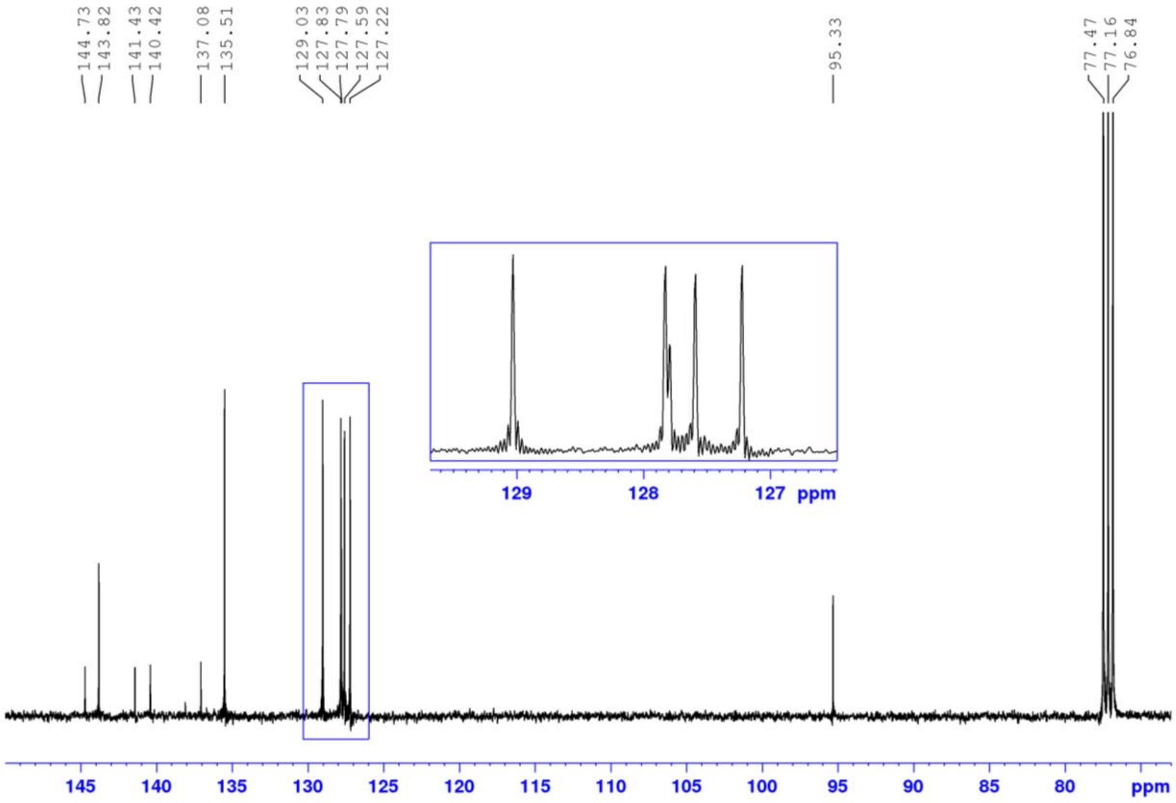

fig. S31. $^{13}C\{^{1}H\}$ NMR (100.6 MHz; $CDCl_3$, 300 K) spectrum of 5.

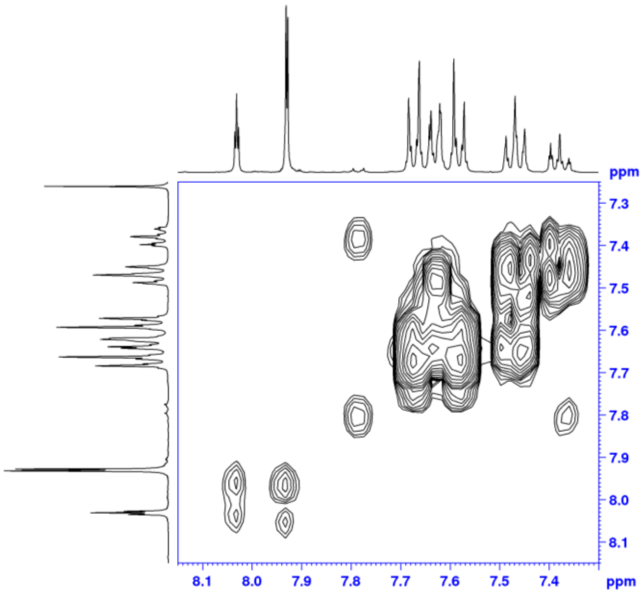

fig. S32. $^{1}H,^{1}H$-COSY NMR (400.1 MHz; $CDCl_3$, 300 K) spectrum of 5.

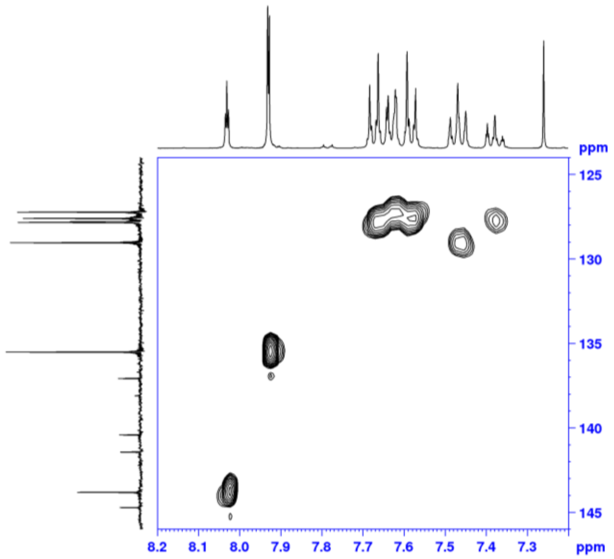

fig. S33. $^{1}H,^{13}C$-HSQC NMR (400.1 MHz; $CDCl_3$, 300 K) spectrum of 5.

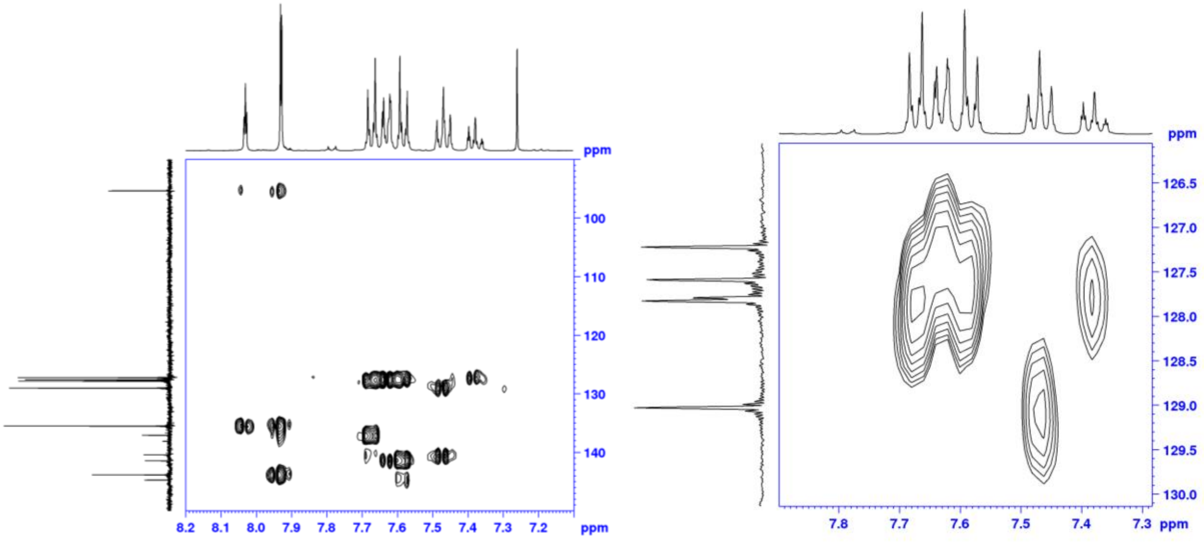

fig. S34. Sections of the $^{1}H,^{13}C$-HMBC NMR (400.1 MHz; $CDCl_3$, 300 K) spectrum of 5.

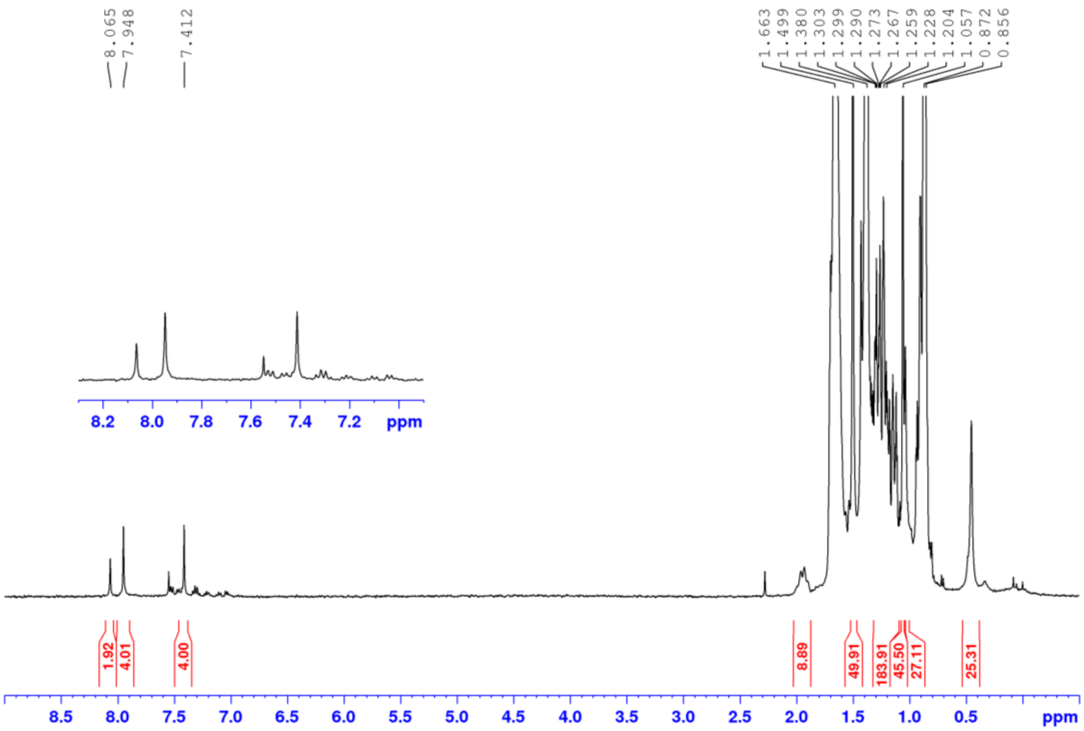

fig. S35. ^{1}H NMR (400.1 MHz; [D_{12}]cyclohexane/C_7H_{14}, 300 K) spectrum of [$Na_8Mg_4(TMP)_{12}$(3,3′,5,5′-*para*-terphenyl-tetra-ide)] 6. Residual signals for methylcylohexane (C_7H_{14}, solvent of crystallization) and *para*-terphenyl and TMP(H) are observed in the ^{1}H NMR spectrum of **4** in [D_{12}]cyclohexane solution due to unavoidable hydrolysis.

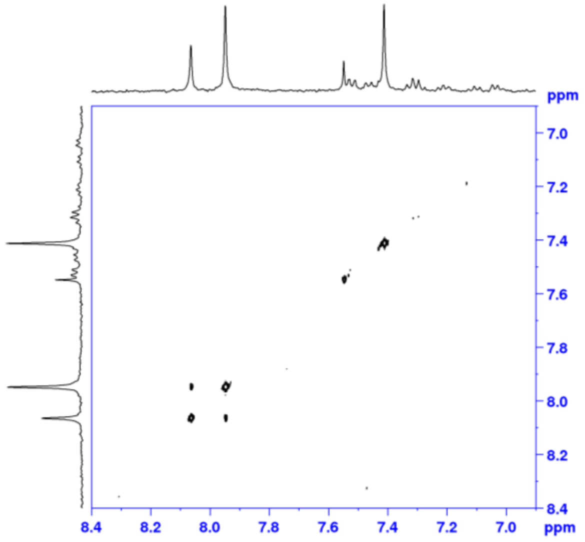

fig. S36. Section of the ^{1}H,^{1}H-COSY NMR (400.1 MHz; [D_{12}]cyclohexane/C_7H_{14}, 300 K) spectrum of [$Na_8Mg_4(TMP)_{12}$(3,3′,5,5′-*para*-terphenyl-tetra-ide)] 6 showing the cross peaks for the aromatic resonances.

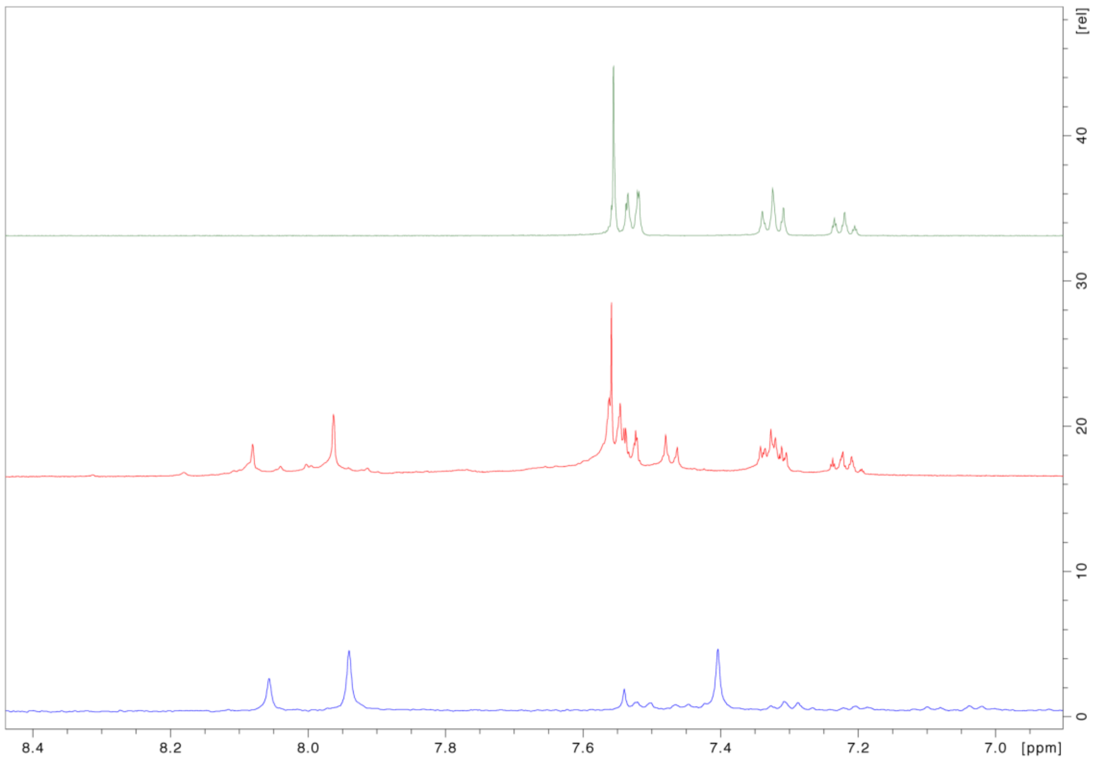

fig. S37. Sections of the ^{1}H NMR (400.1 MHz; [D$_{12}$]cyclohexane, 300 K) spectra of *para*-terphenyl (top; green), 4 (middle; red), and [$Na_8Mg_4(TMP)_{12}$(3,3′,5,5′-*para*-terphenyl-tetra-ide)] 6 (bottom; blue). Residual signals *para*-terphenyl are observed in the ^{1}H NMR spectra of **4** and **6** in [D$_{12}$]cyclohexane solution due to unavoidable hydrolysis of the NMR samples.

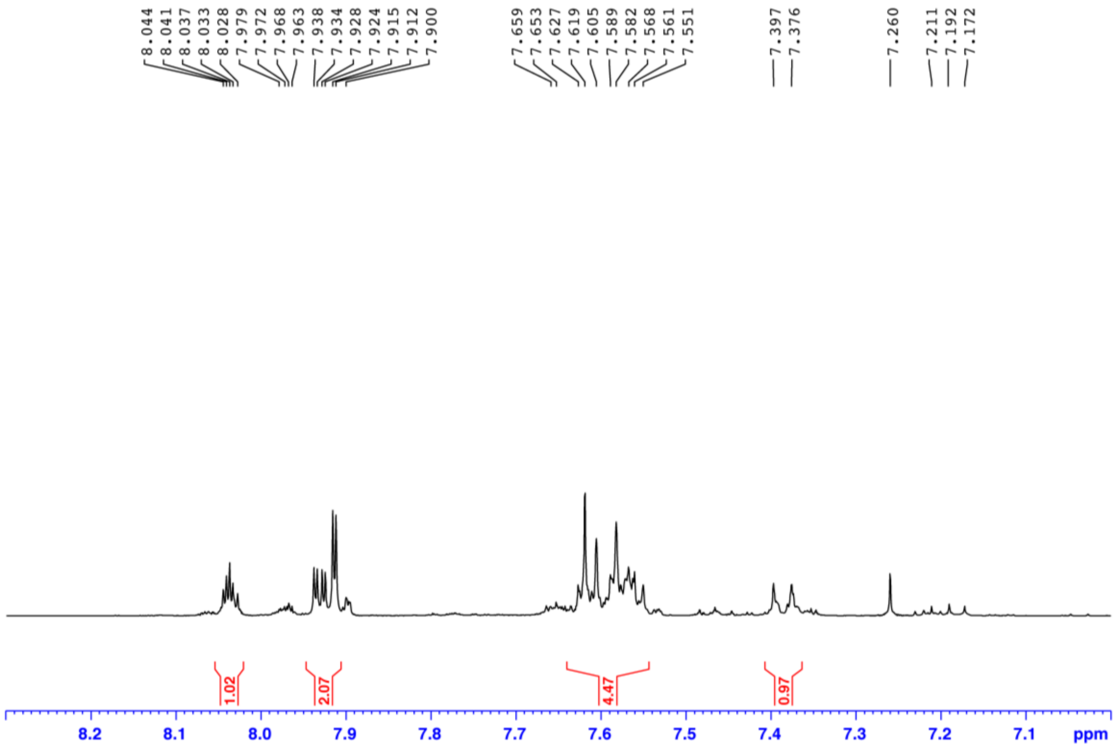

fig. S38. ^{1}H NMR (400.1 MHz; $CDCl_3$, 300 K) spectrum of 7.

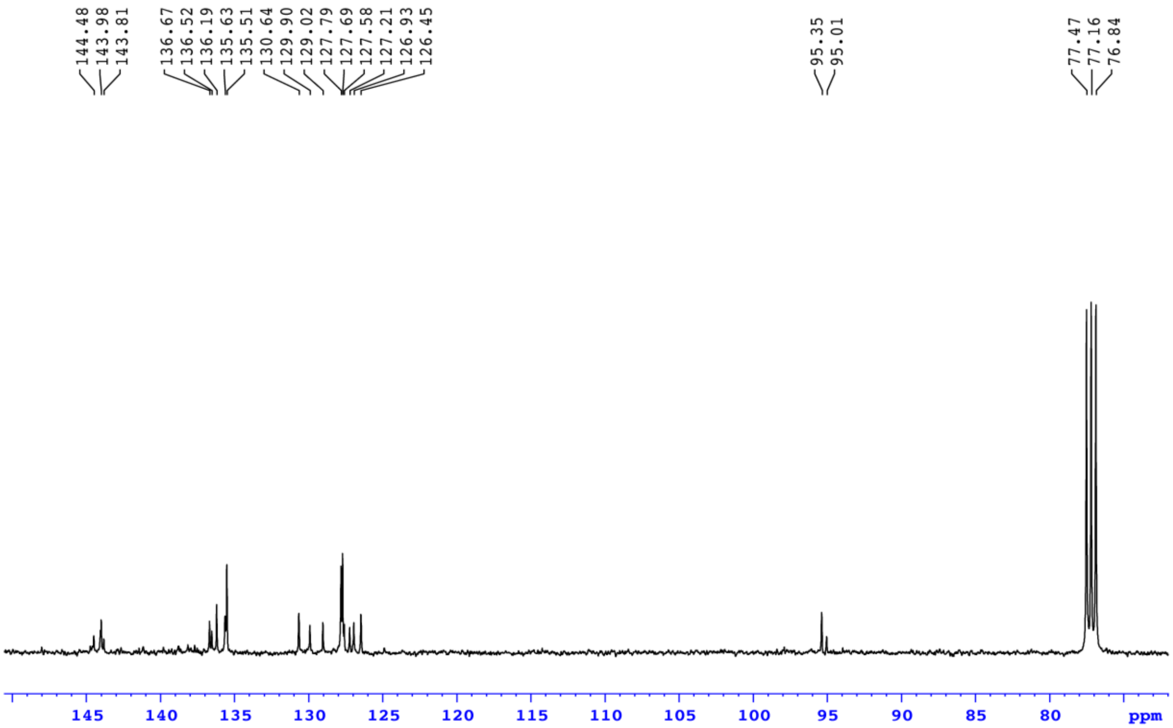

fig. S39. ^{13}C NMR (100.6 MHz; $CDCl_3$, 300 K) spectrum of 7.

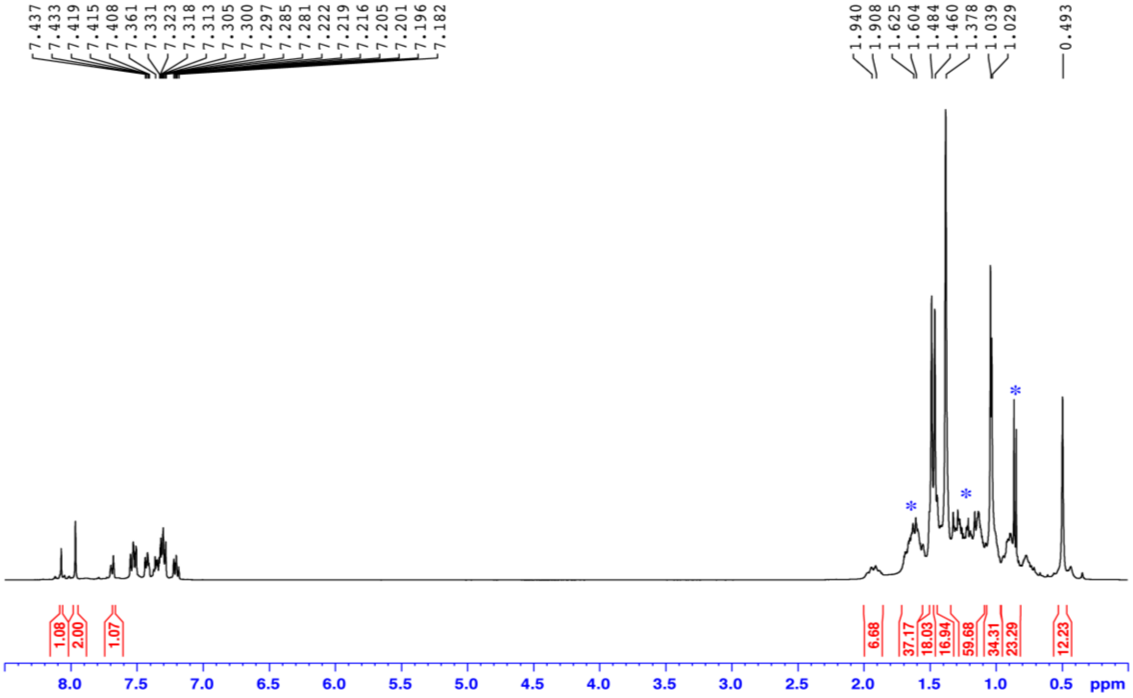

fig. S40. ^{1}H NMR (400.1 MHz; [D_{12}]cyclohexane, 300 K) spectrum of [$Na_4Mg_2(TMP)_6$(3,5-*meta*-terphenyl-di-ide)] 8. Signals corresponding to methylcylohexane (C_7H_{14}, solvent of *in situ* preparation) are denoted with blue asterisks. Residual signals corresponding to *meta*-terphenyl and TMP(H) are present within the [D_{12}]cyclohexane solution of **8** due to unavoidable hydrolysis.

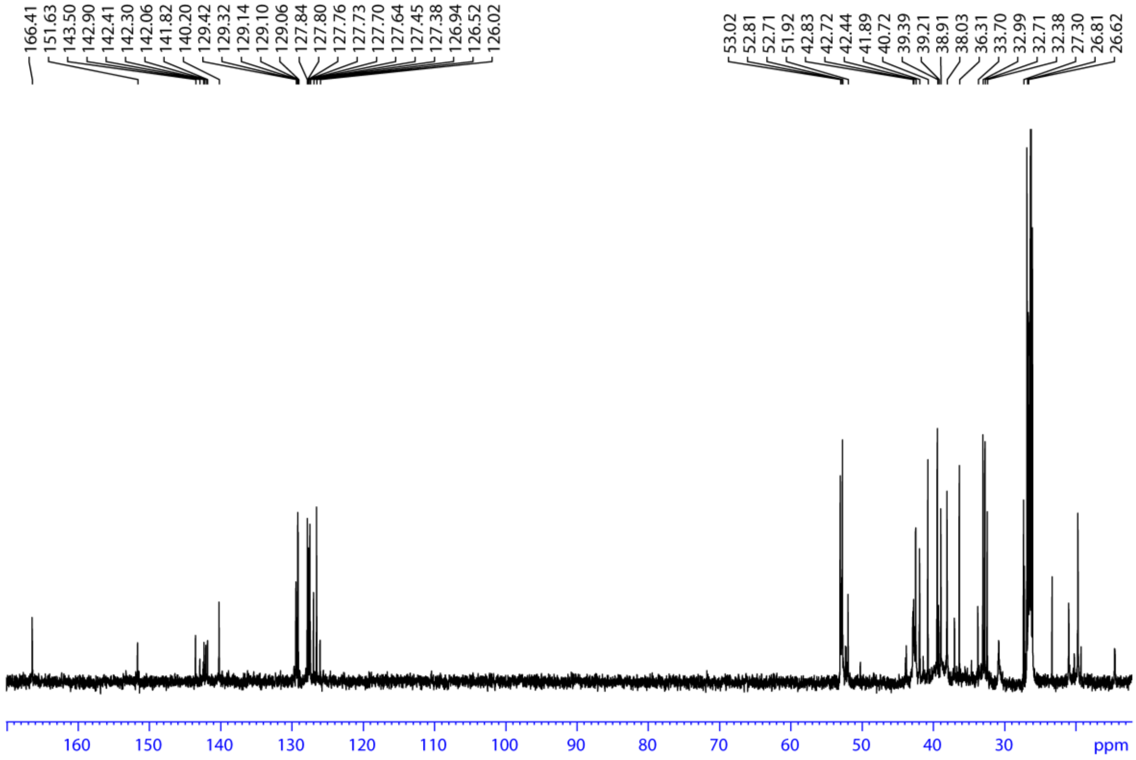

fig. S41. $^{13}C\{^{1}H\}$ NMR (100.6 MHz; [D_{12}]cyclohexane, 300 K) spectrum of [$Na_4Mg_2(TMP)_6$(3,5-*meta*-terphenyl-di-ide)] 8. Residual signals corresponding to *meta*-terphenyl and TMP(H) are present within the [D_{12}]cyclohexane solution of **8** due to unavoidable hydrolysis.

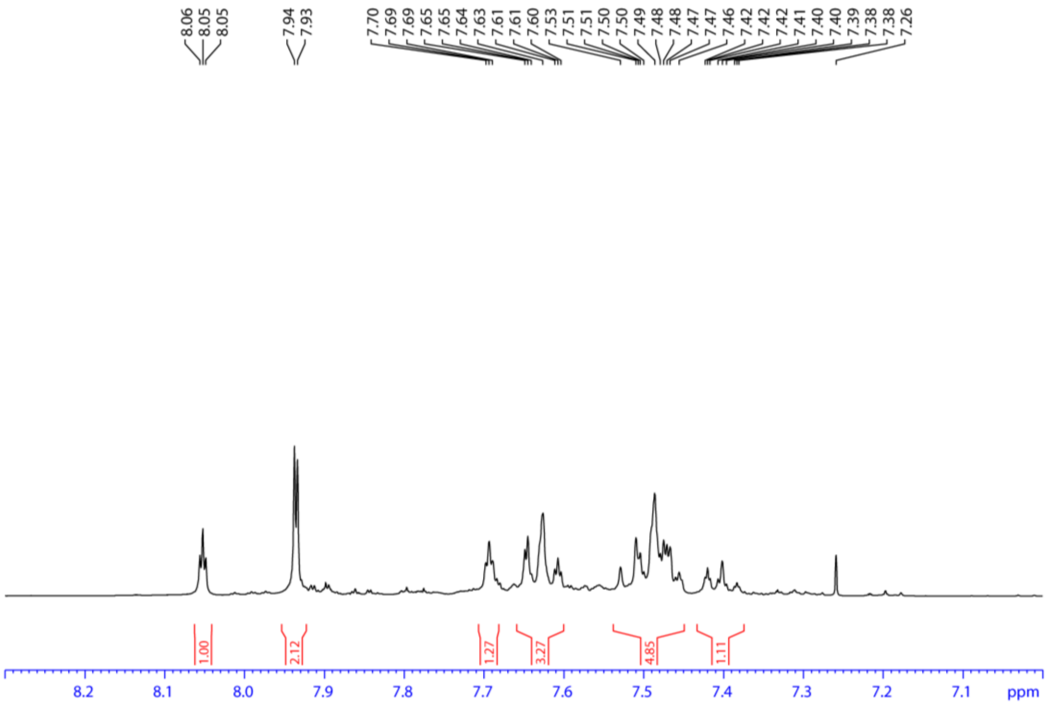

fig. S42. ^{1}H NMR (400.1 MHz; 300 K, $CDCl_3$) spectrum of 9.

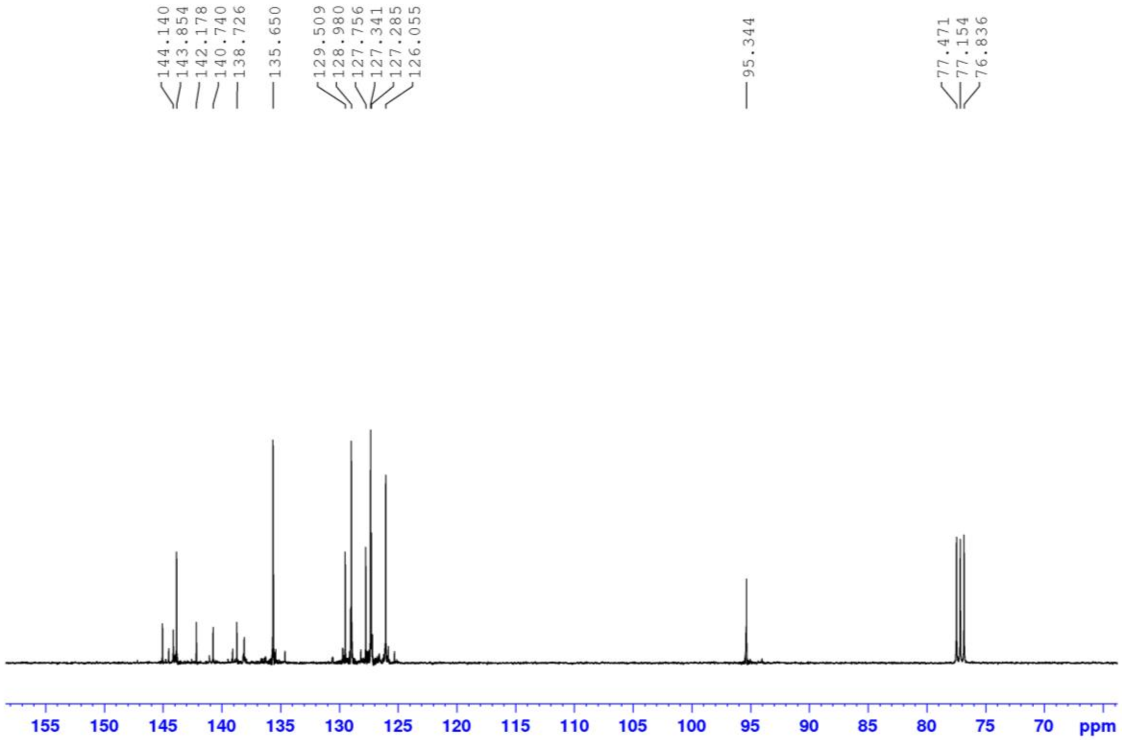

fig. S43. $^{13}C\{^{1}H\}$ NMR (100.6 MHz; 300 K, $CDCl_3$) spectrum of 9.

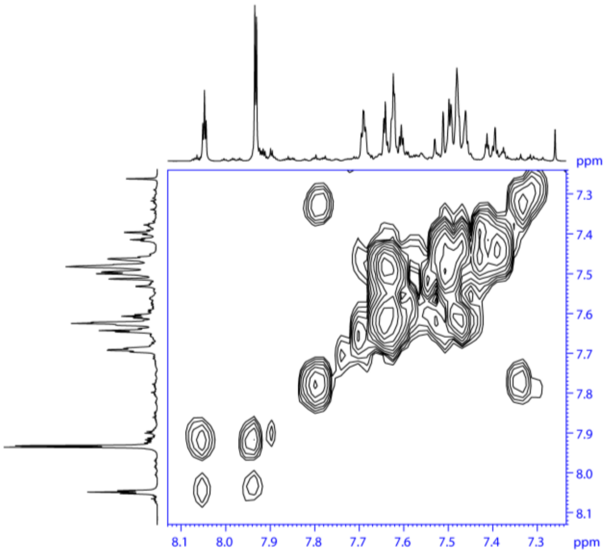

fig. S44. $^{1}H,^{1}H$-COSY NMR (400.1 MHz; 300 K, $CDCl_3$) spectrum of 3,5-diiodo-*meta*-terphenyl 9.

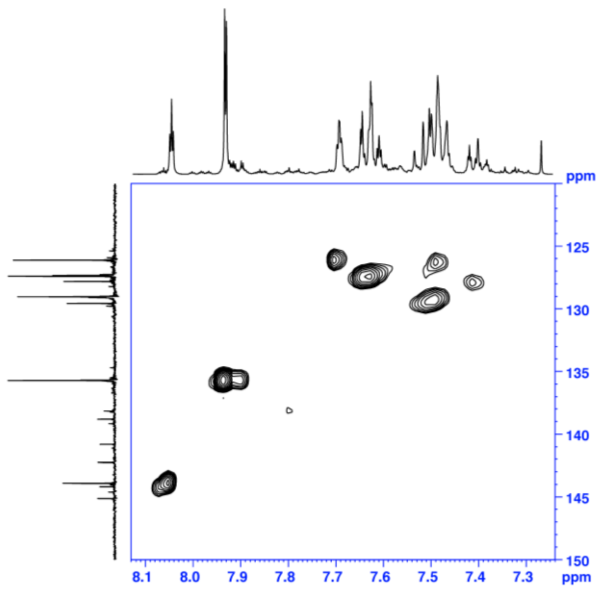

fig. S45. $^{1}H,^{13}C$-HSQC NMR (400.1 MHz; 300 K, $CDCl_3$) spectrum of 9.

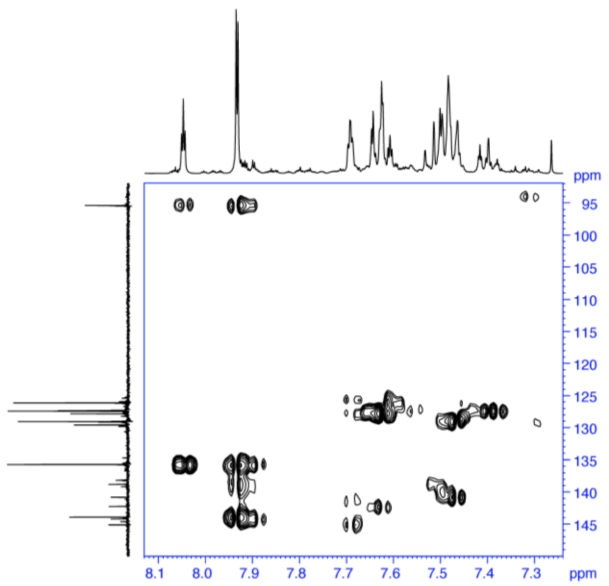

fig. S46. $^{1}H,^{13}C$-HMBC NMR (400.1 MHz; 300 K, $CDCl_3$) spectrum of 9.

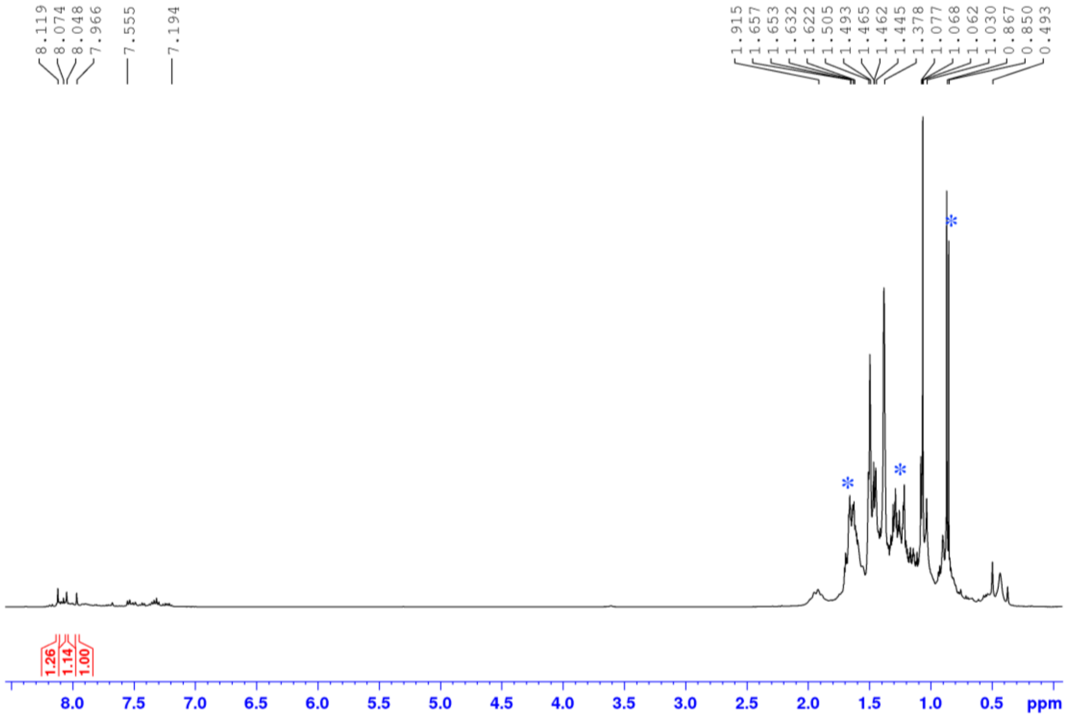

fig. S47. ¹H NMR (400.1 MHz; [D_{12}]cyclohexane, 300 K) spectrum of [$Na_8Mg_4TMP_{12}$(3,3′,5,3′-*meta*-terphenyl-tetra-ide)] 10. Signals corresponding to methylcylohexane (C_7H_{14}, solvent of *in situ* preparation) are denoted with blue asterisks. Residual signals corresponding to *meta*-terphenyl and TMP(H) are present within the [D_{12}]cyclohexane solution of **10** due to unavoidable hydrolysis.

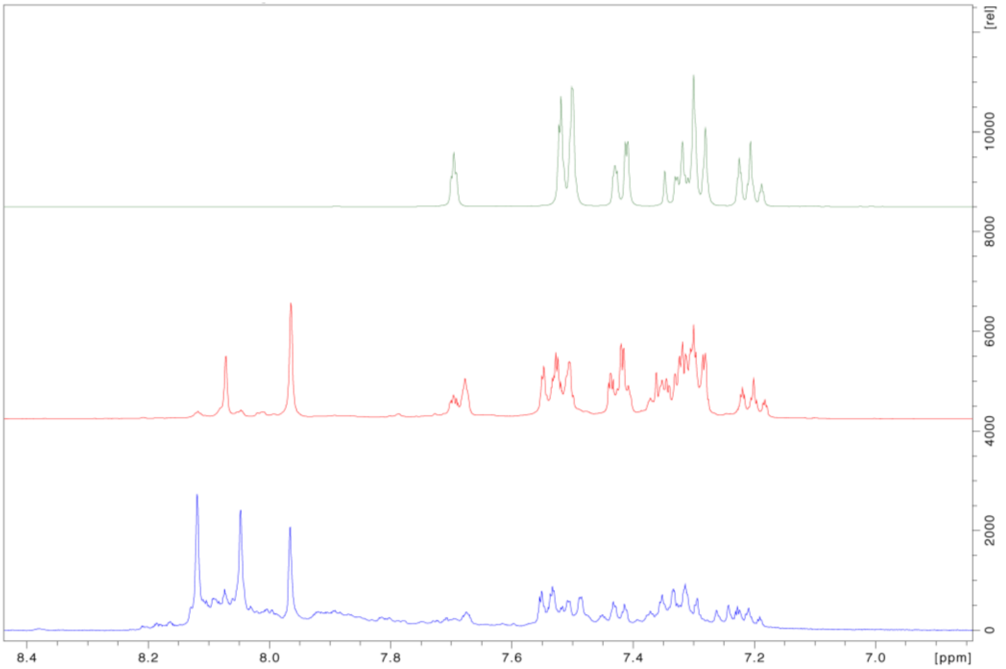

fig. S48. Sections of the ¹H NMR (400.1 MHz; [D_{12}]cyclohexane, 300 K) spectra of *meta*-terphenyl (top; green), [$Na_4Mg_2(TMP)_6$(3,5-*meta*-terphenyl-di-ide)] 8 (middle; red), and [$Na_8Mg_4TMP_{12}$(3,3′,5,3′-*meta*-terphenyl-tetra-ide)] 10 (bottom; blue) showing the aromatic resonances. Residual signals for *meta*-terphenyl are observed in the ¹H NMR spectrum of **8** and **10** in [D_{12}]cyclohexane solution due to unavoidable hydrolysis.

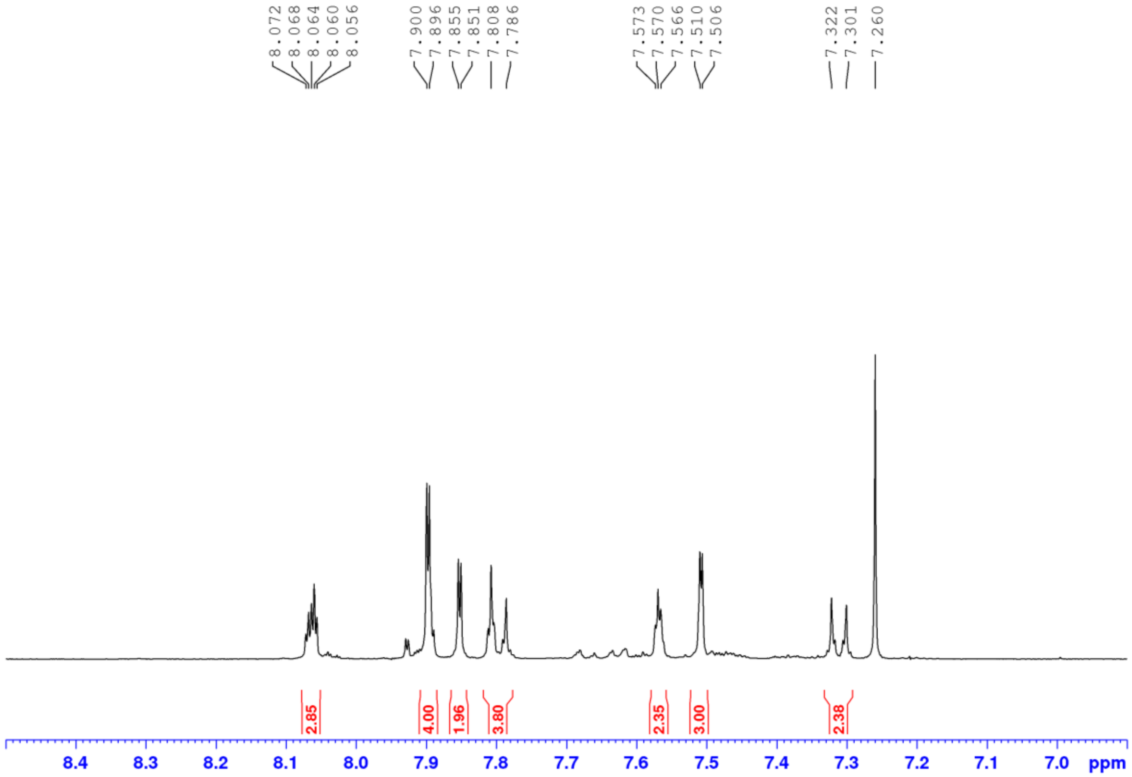

fig. S49. **^{1}H NMR (400.1 MHz; $CDCl_3$, 300 K) spectrum of 11.**

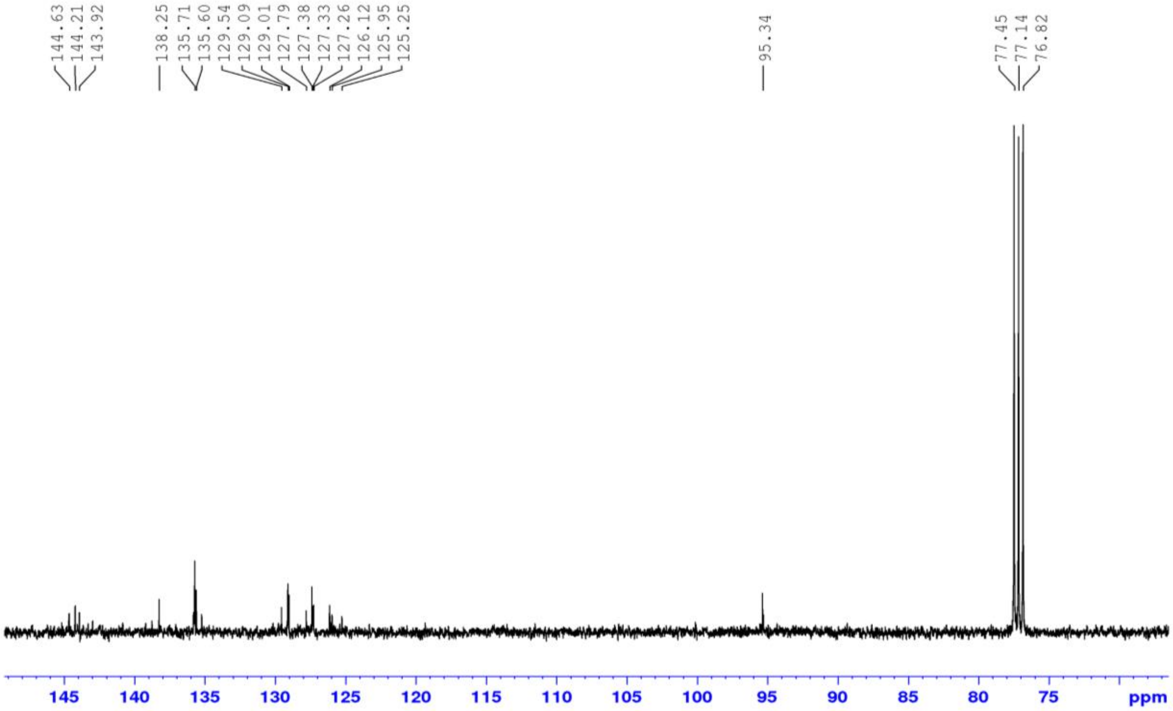

fig. S50. **$^{13}C\{^{1}H\}$ NMR (100.6 MHz; $CDCl_3$, 300 K) spectrum of 11.**

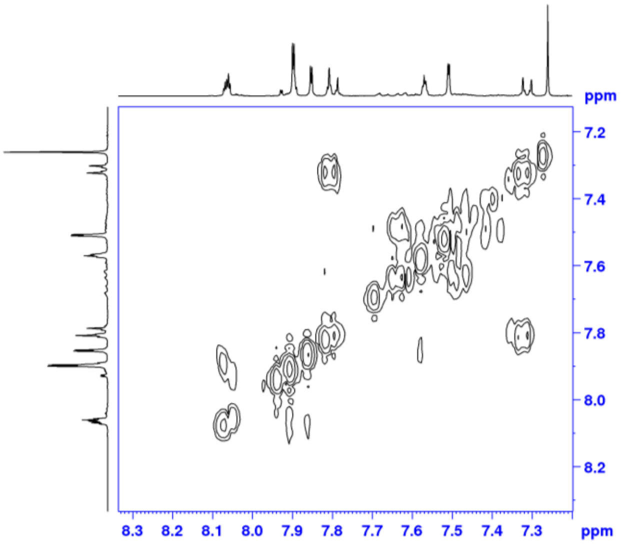

fig. S51. **^{1}H,^{1}H-COSY NMR (400.1 MHz; $CDCl_3$, 300 K) spectrum of 9.**

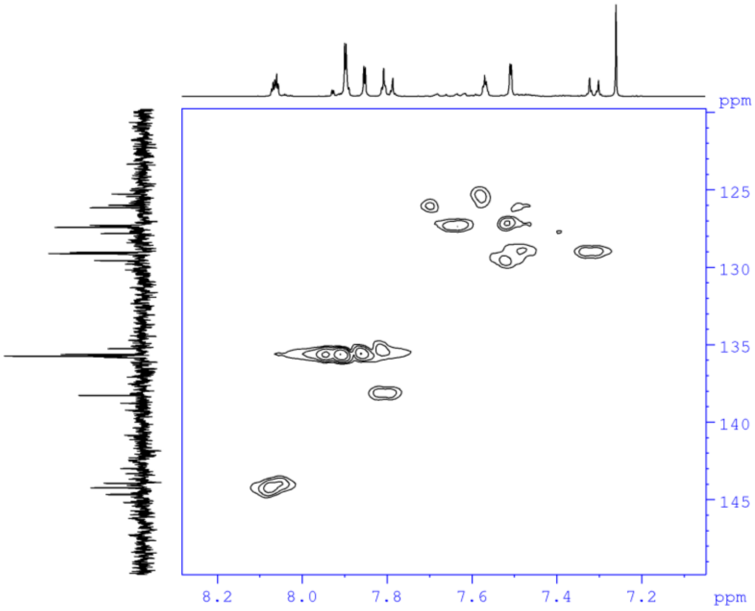

fig. S51. **^{1}H,^{1}H-COSY NMR (400.1 MHz; $CDCl_3$, 300 K) spectrum of 9.**

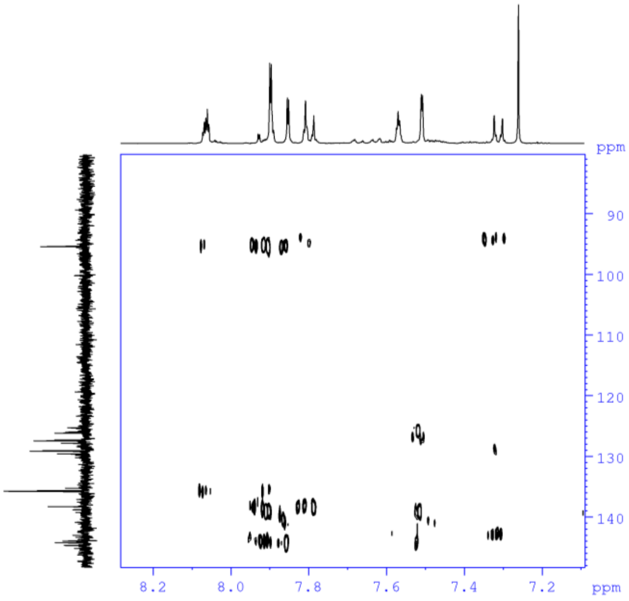

fig. S53. ^{1}H,^{13}C-HMBC NMR (400.1 MHz; $CDCl_3$, 300 K) spectrum of 9.

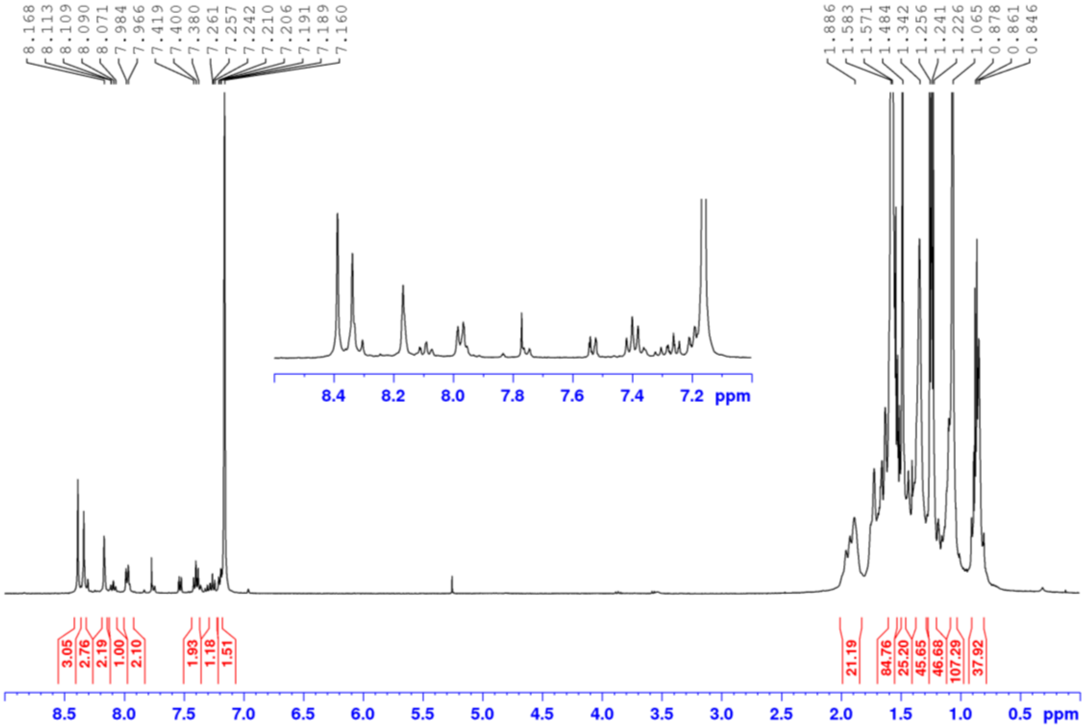

fig. S54. ^{1}H NMR (400.1 MHz; [D_6]benzene, 300 K) spectrum of {Na_8Mg_4[TMP]$_{12}$[3,3″,5,5″-(1′,3′,5′-triphenylbenzene-tetra-ide)]} 12. Residual signals for methylcyclohexane (C_7H_{14}), solvent of crystallization, and TMP(H) and 1,3,5-triphenylbenzene are observed in the ^{1}H NMR spectra of **12** in [D_6]benzene solution due to unavoidable hydrolysis of the NMR spectroscopic sample and its low solubility.

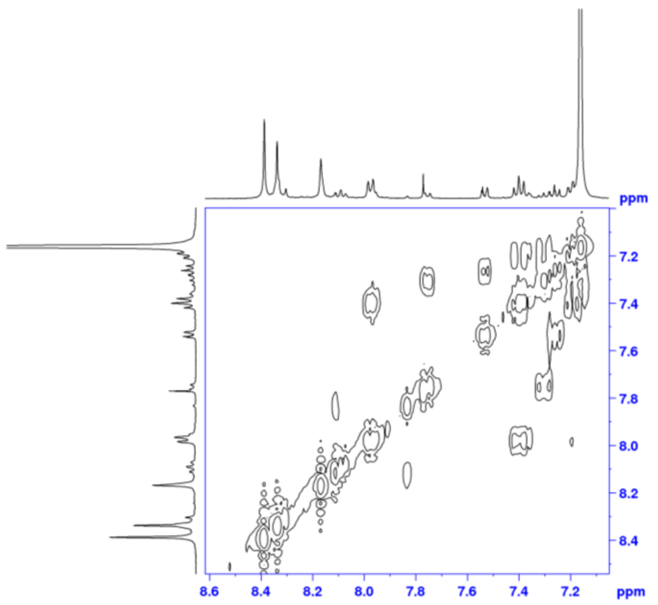

fig. S55. Section of the ^{1}H,^{1}H-COSY NMR (400.1 MHz; [D$_6$]benzene, 300 K) spectrum of 12 showing the cross peaks for the aromatic resonances.

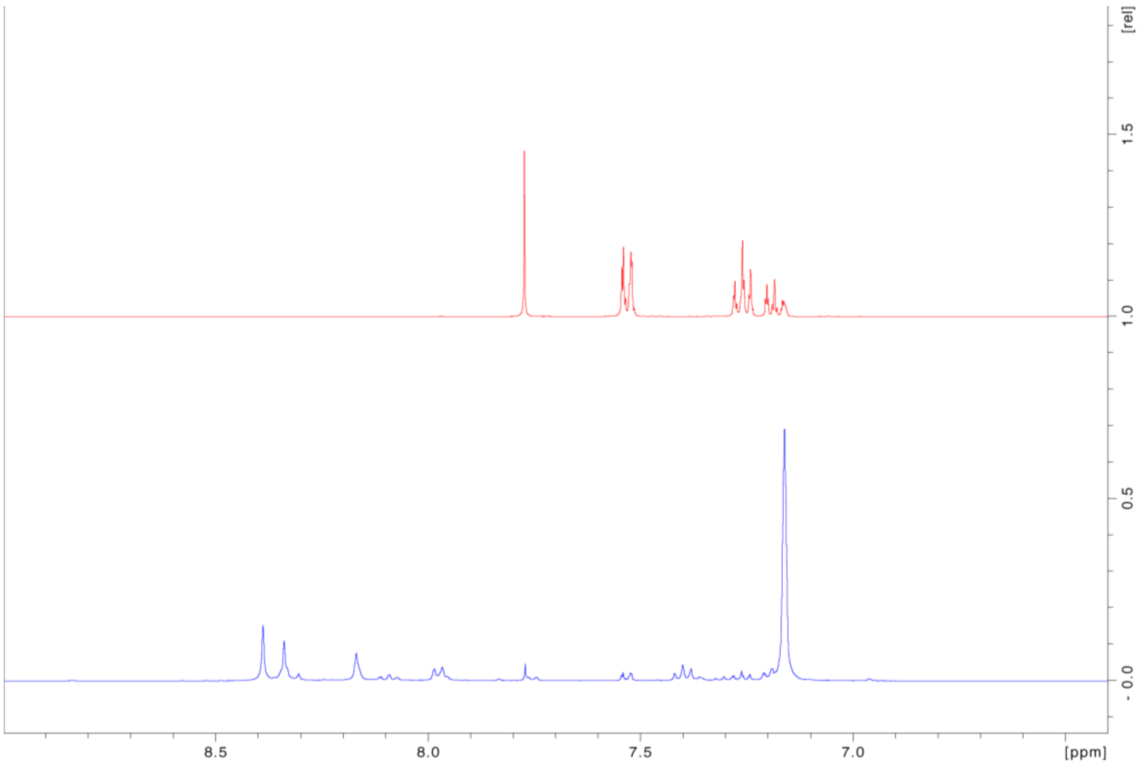

fig. S56. ^{1}H NMR (400.1 MHz; [D$_6$]benzene, 300 K) spectra of tpb (top; red) and 12 (bottom; blue). Residual signals for 1,3,5-triphenylbenzene are observed in the ^{1}H NMR spectrum of **12** in [D$_6$]benzene solution due to unavoidable hydrolysis of the NMR sample.

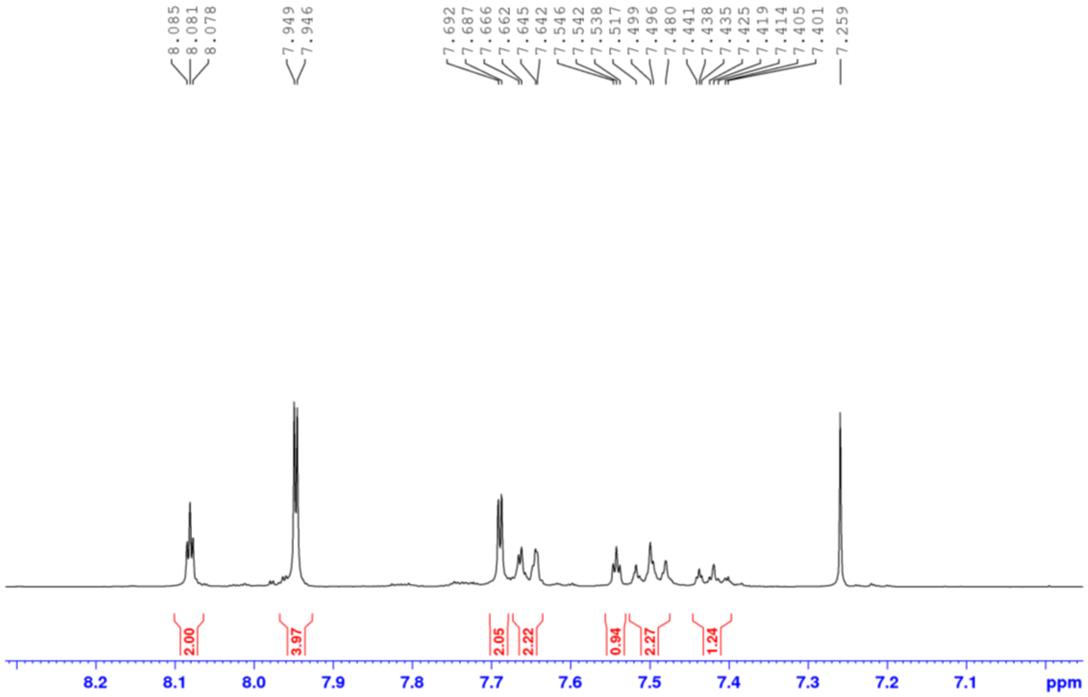

fig. S57. **^{1}H NMR (400.1 MHz; $CDCl_3$, 300 K) spectrum of 3,3″,5,5″-tetraiodo-5′-phenyl-benzene 13.**

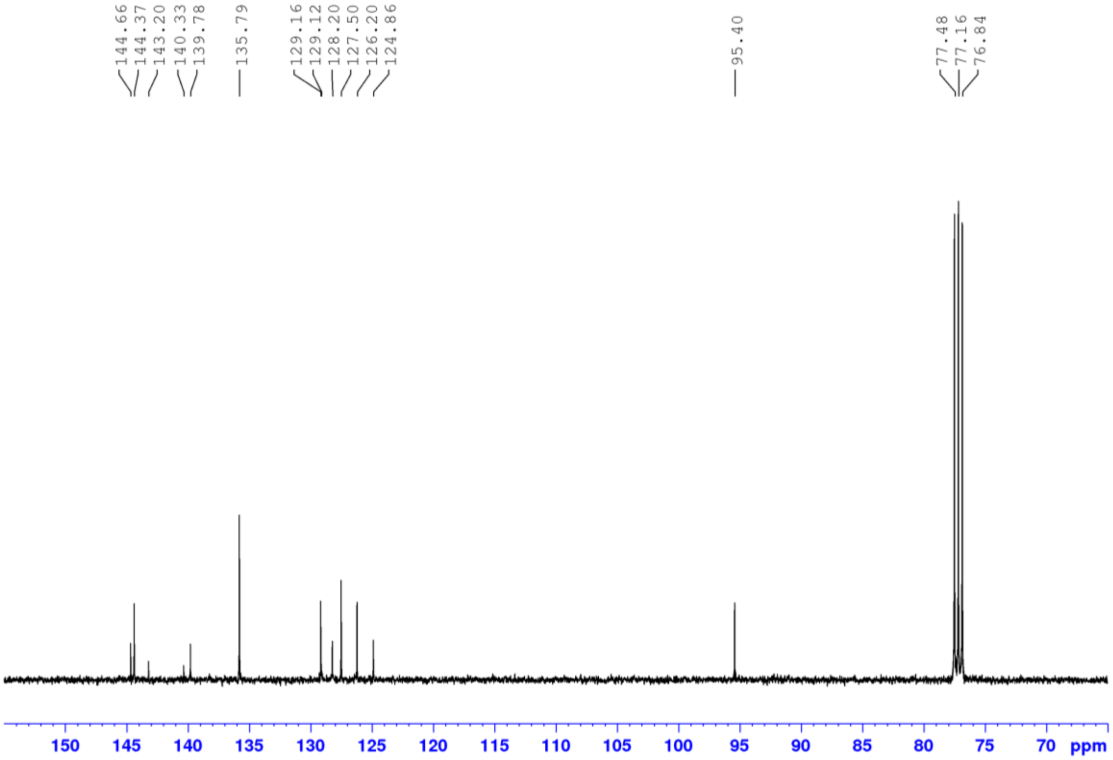

fig. S58. **$^{13}C\{^{1}H\}$ NMR (100.6 MHz; $CDCl_3$, 300 K) spectrum of 13.**

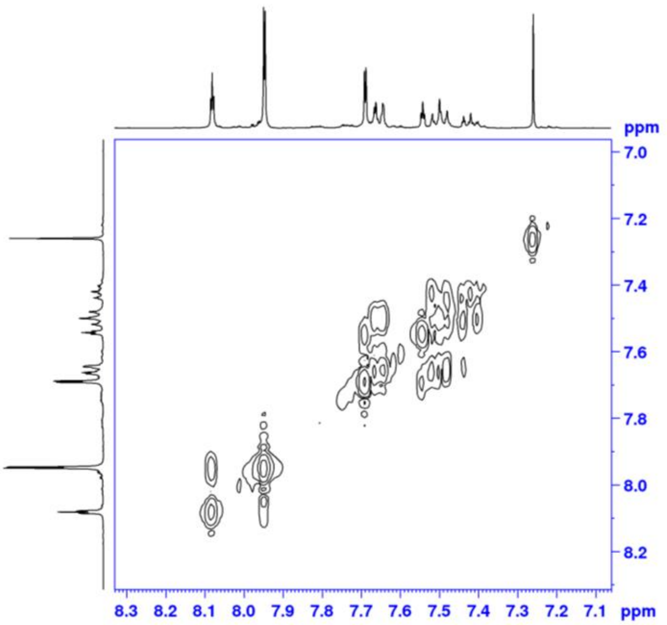

fig. S59. $^{1}H,^{1}H$-COSY NMR (400.1 MHz; $CDCl_3$, 300 K) spectrum of 13.

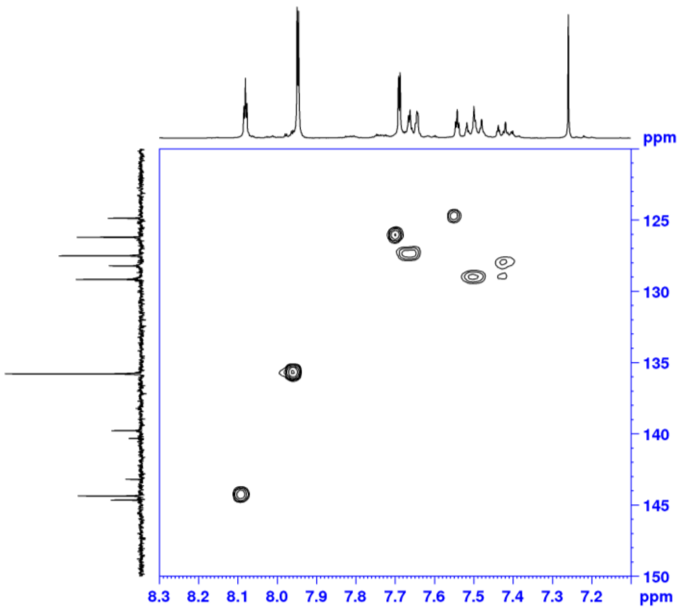

fig. S60. $^{1}H,^{13}C$-HSQC NMR (400.1 MHz; $CDCl_3$, 300 K) spectrum of 13.

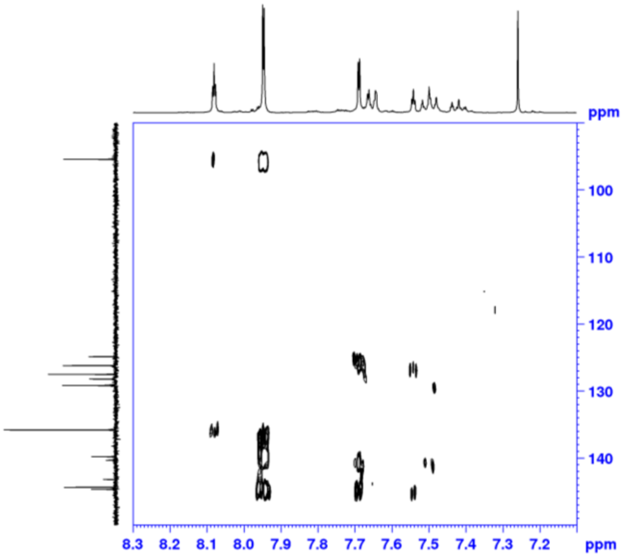

fig. S61. $^{1}H,^{13}C$-HMBC NMR (400.1 MHz; $CDCl_3$, 300 K) spectrum sections of 13.

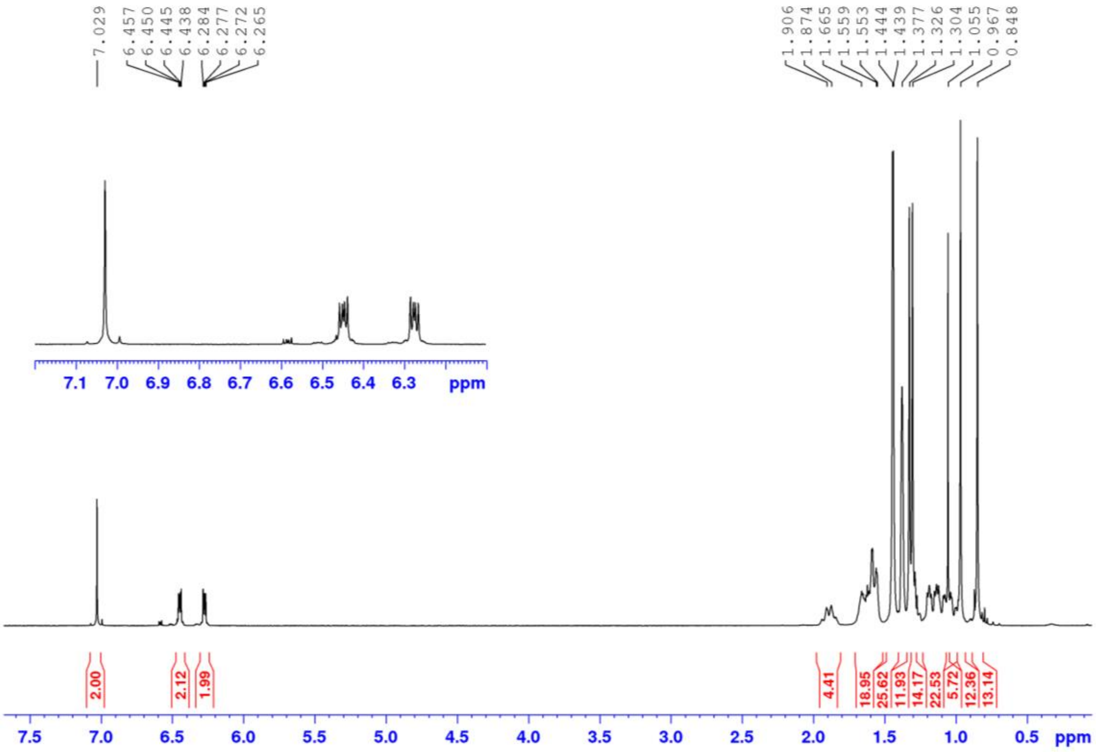

fig. S62. ^{1}H NMR (400.1 MHz; $[D_{12}]$cyclohexane, 300 K) spectrum of 14.

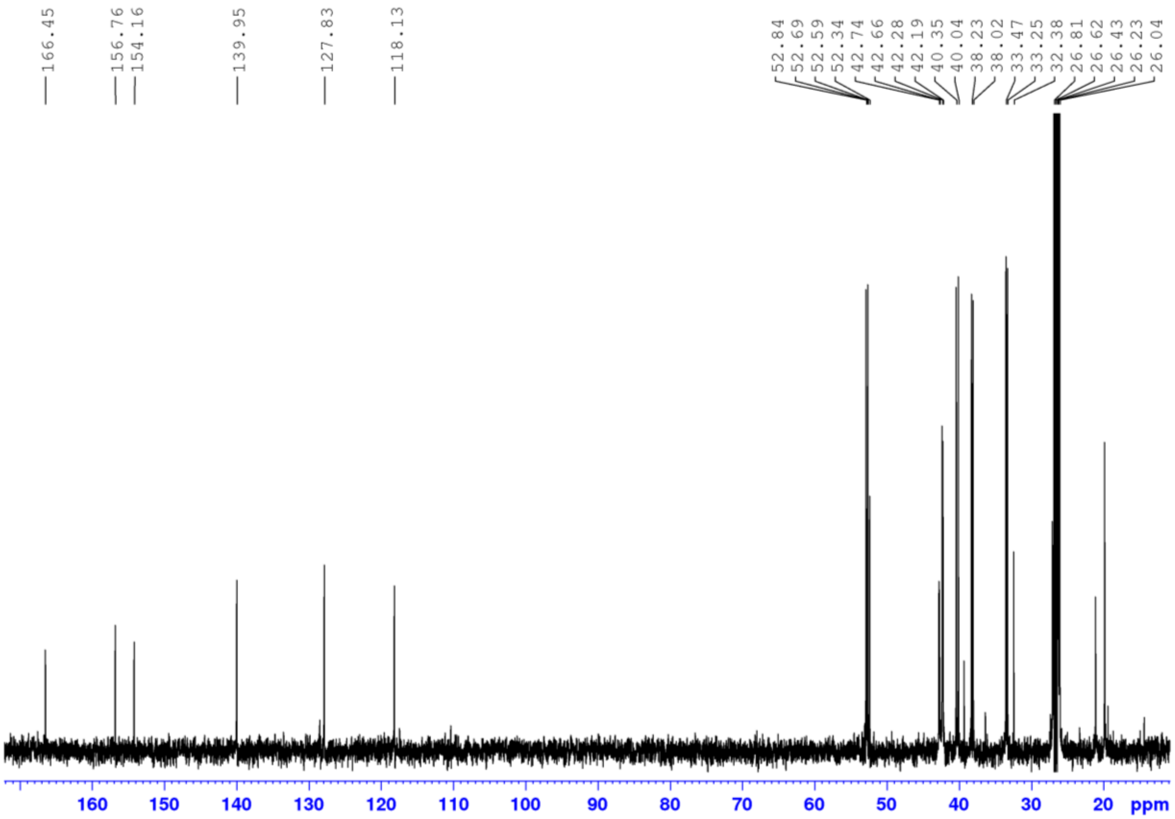

fig. S63. $^{13}C\{^{1}H\}$ **NMR (100.6 MHz; [D_{12}]cyclohexane, 300 K) spectrum of 14.**

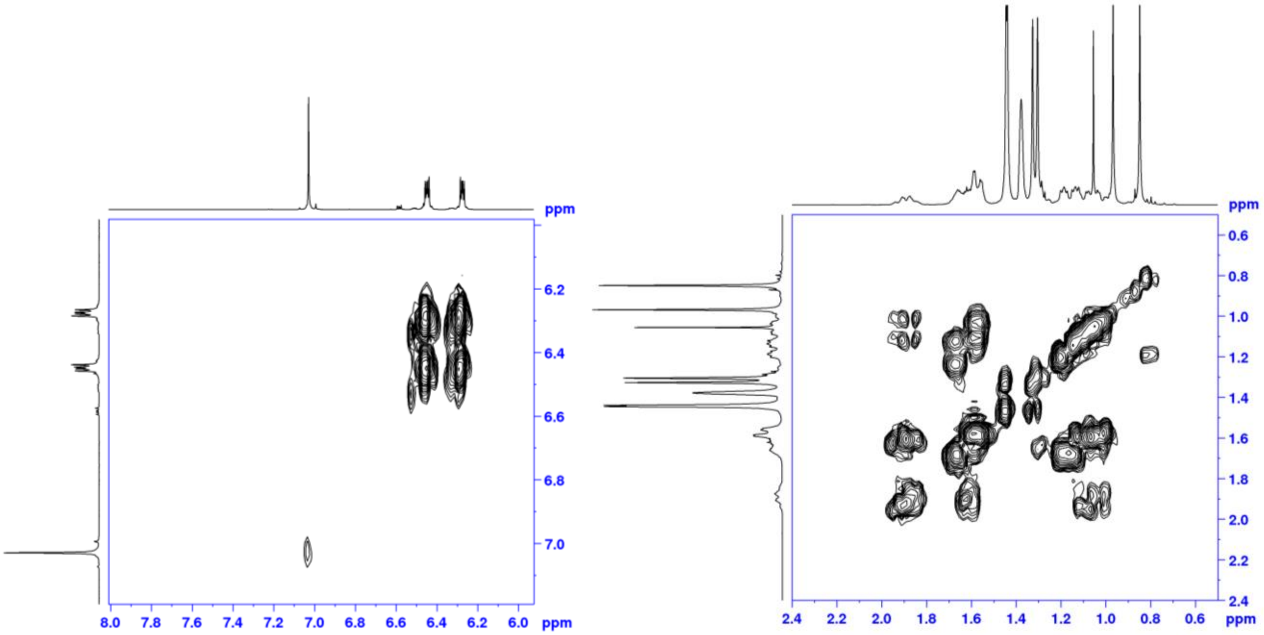

fig. S64. Sections of the ^{1}H,^{1}H-COSY NMR (400.1 MHz; [D_{12}]cyclohexane, 300 K) spectrum of 14.

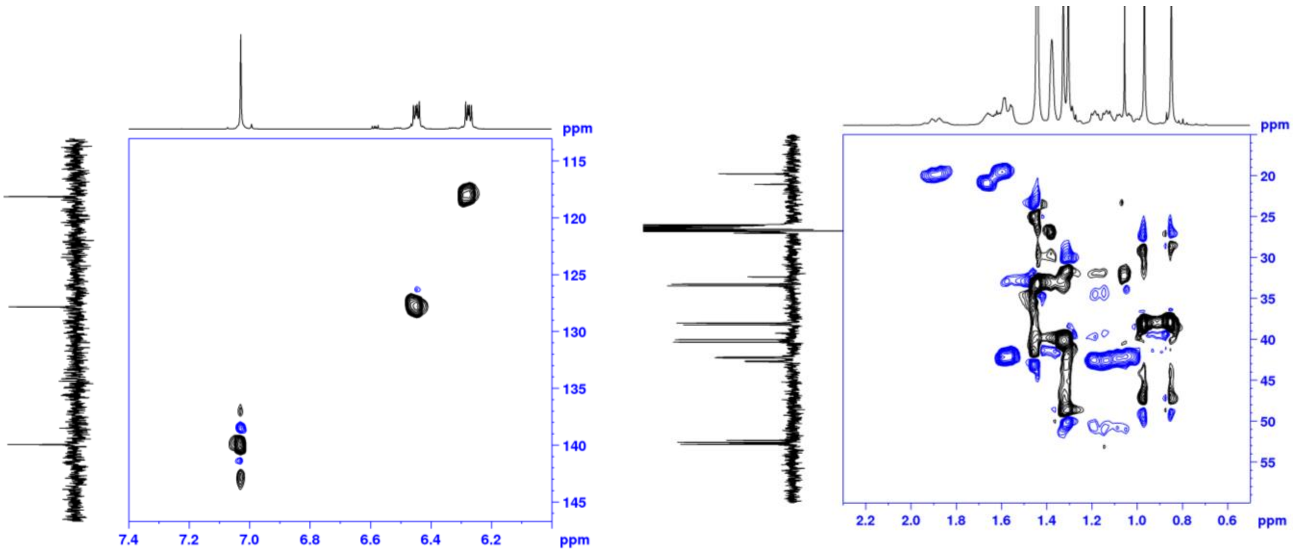

fig. S65. Sections of the phase-sensitive $^{1}H,^{13}C$-HSQC NMR (400.1 MHz; [D_{12}]cyclohexane, 300 K) spectrum of 14. Cross-peaks corresponding to CH_3 and CH groups are pictured in black whilst they are pictured in blue for CH_2 groups.

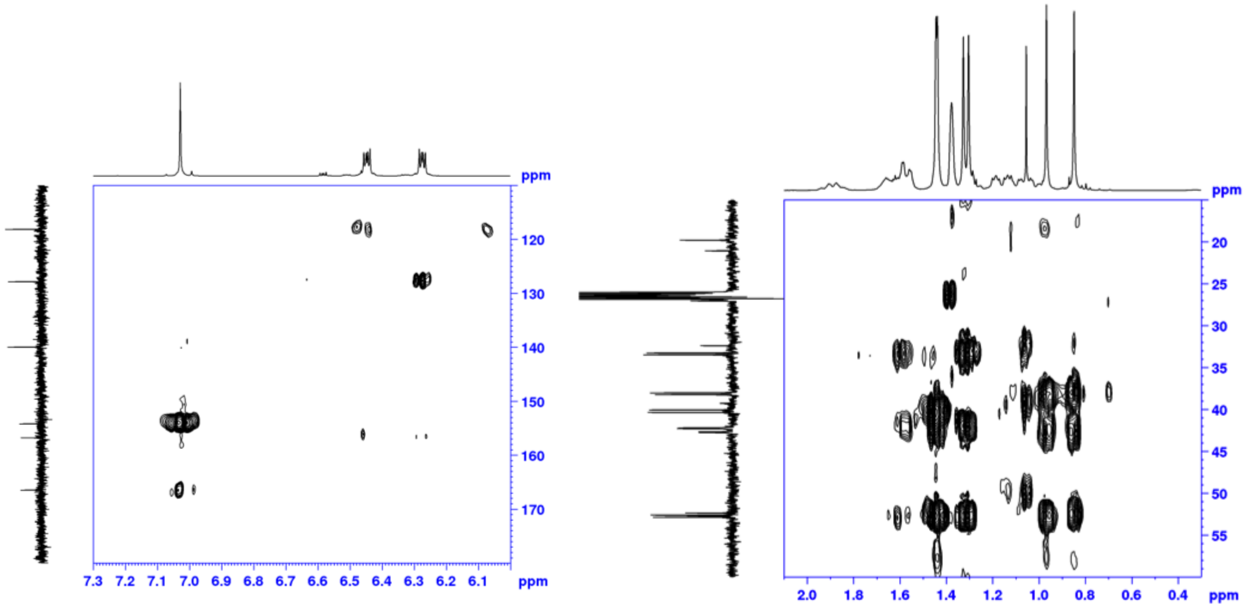

fig. S66. Sections of the $^{1}H,^{13}C$-HMBC NMR (400.1 MHz; [D_{12}]cyclohexane, 300 K) spectrum of 14.

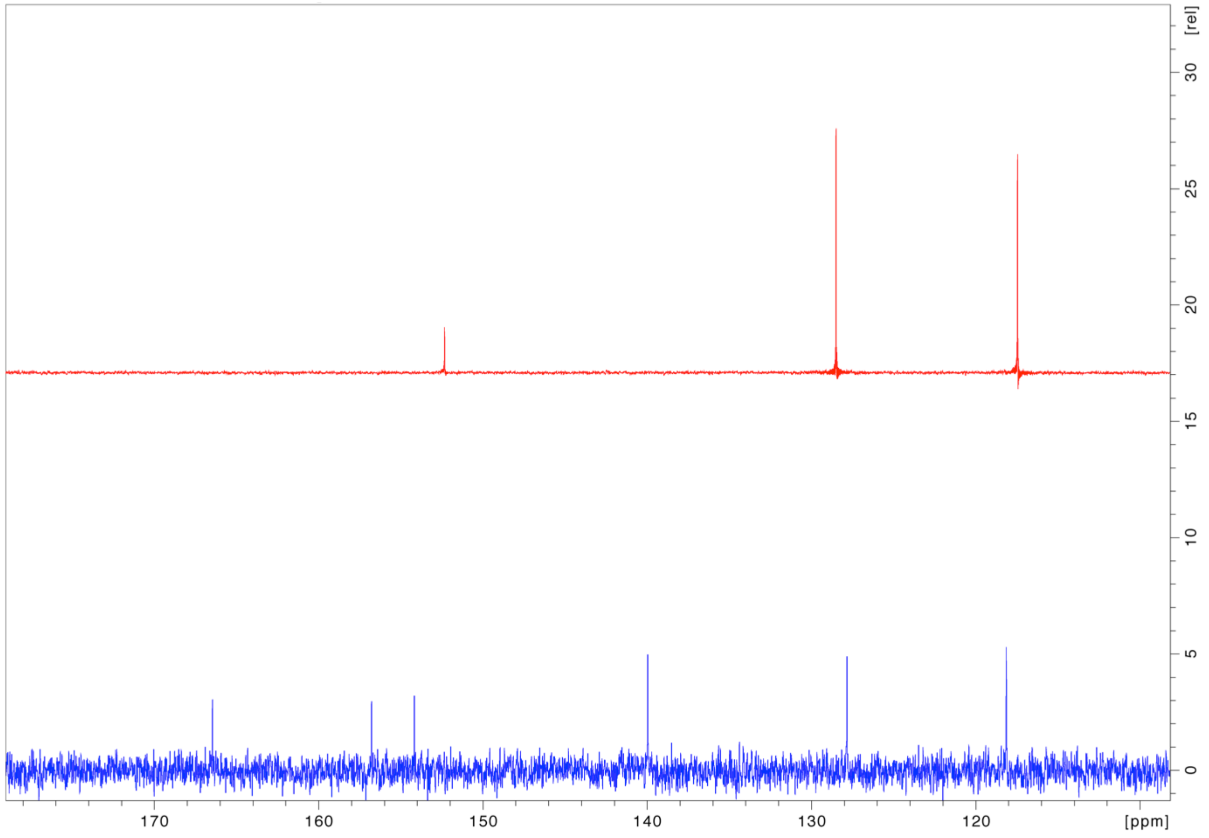

fig. S67. $^{13}C\{^{1}H\}$ NMR (100.6 MHz; [D_{12}]cyclohexane, 300 K) section of the spectra of biphenylene (top; red) and isolated 14 (bottom; blue, major conformer).

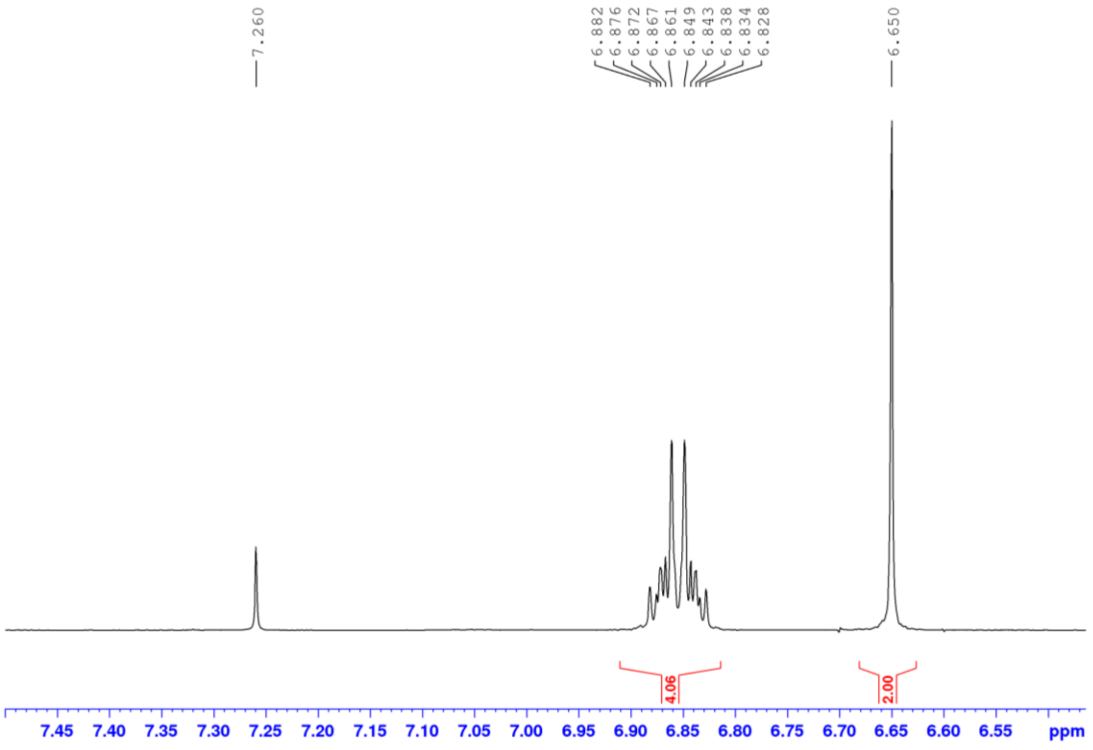

fig. S67. $^{13}C\{^1H\}$ NMR (100.6 MHz; [D_{12}]cyclohexane, 300 K) section of the spectra of biphenylene (top; red) and isolated 14 (bottom; blue, major conformer).

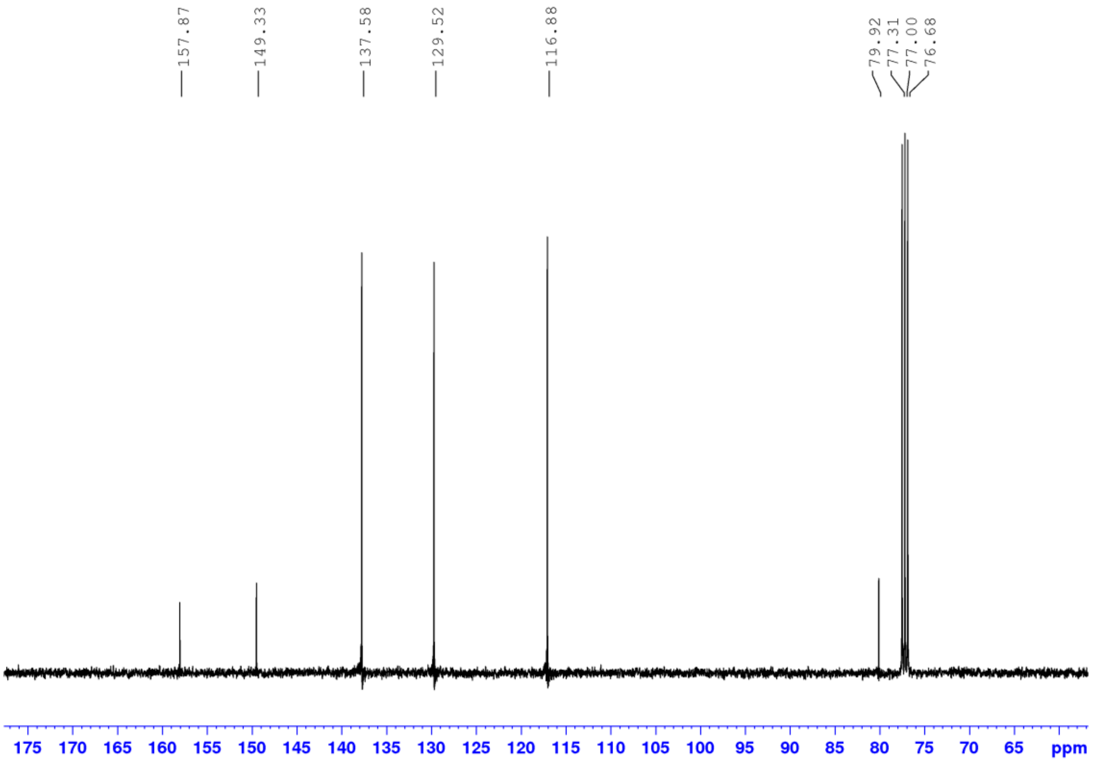

fig. S69. $^{13}C\{^1H\}$ NMR (100.6 MHz; $CDCl_3$, 300 K) spectrum of 15.

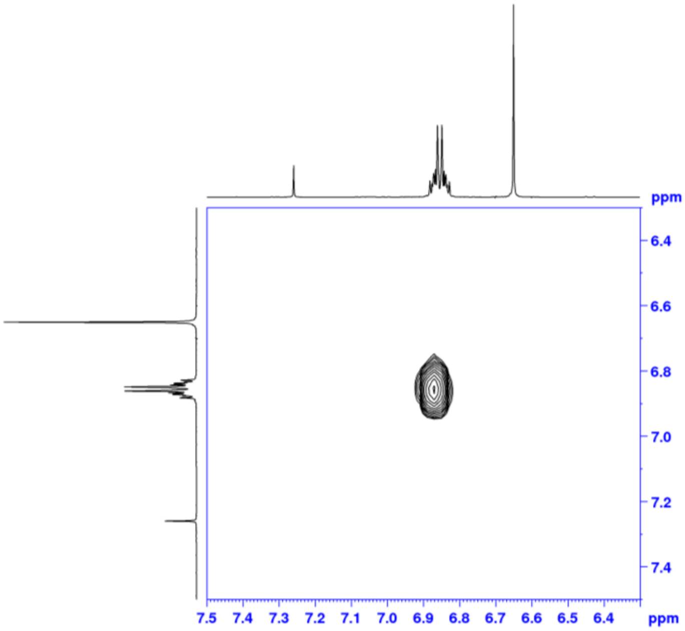

fig. S70. $^1H,^1H$-COSY NMR (400.1 MHz; $CDCl_3$, 300 K) spectrum of 15.

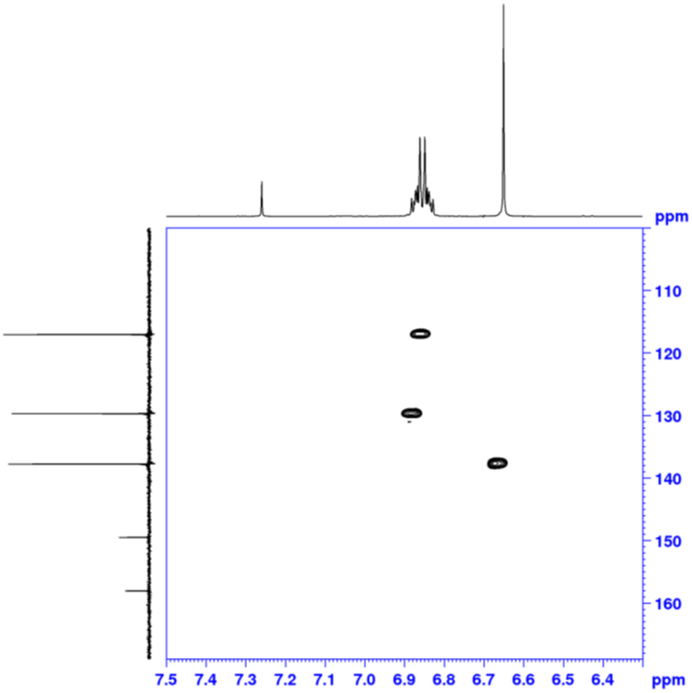

fig. S71. $^{1}H,^{13}C$-HSQC NMR (400.1 MHz; $CDCl_3$, 300 K) spectrum of 15.

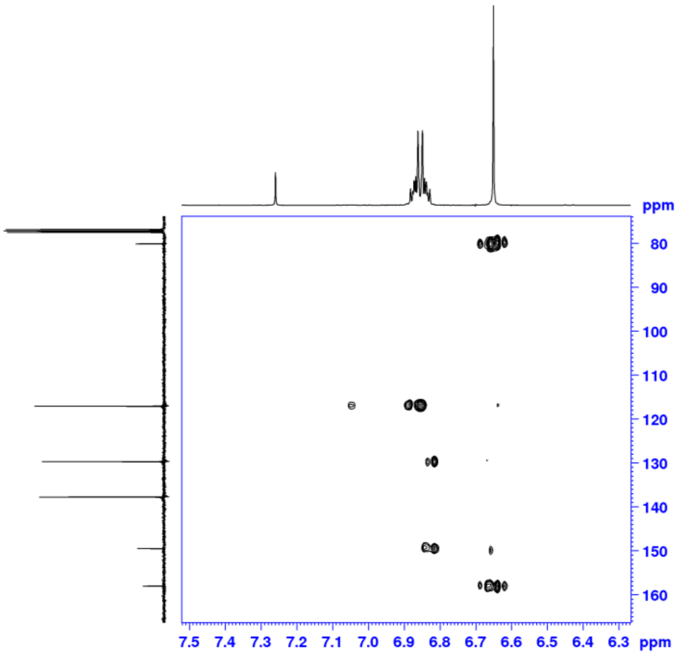

fig. S72. $^{1}H,^{13}C$-HMBC NMR (400.1 MHz; $CDCl_3$, 300 K) spectrum of 15.

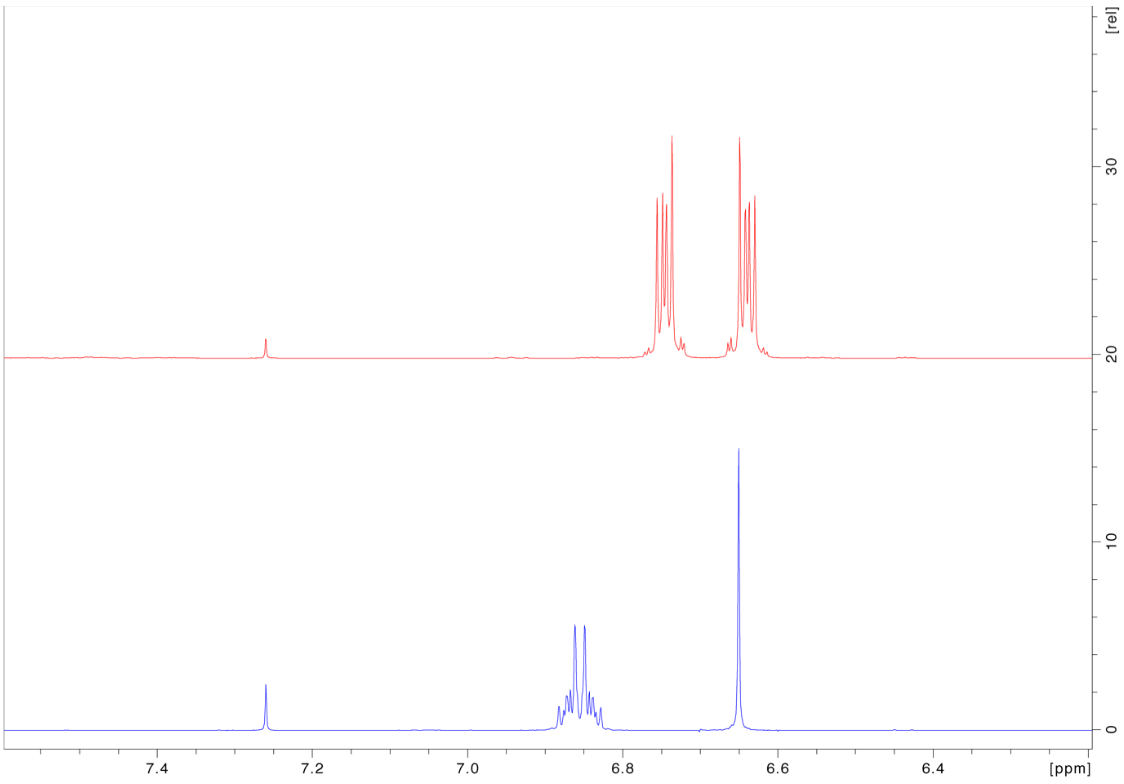

fig. S73. **^{1}H NMR (400.1 MHz; $CDCl_3$, 300 K) spectra of biphenylene (top; red) and 15 (bottom; blue).**

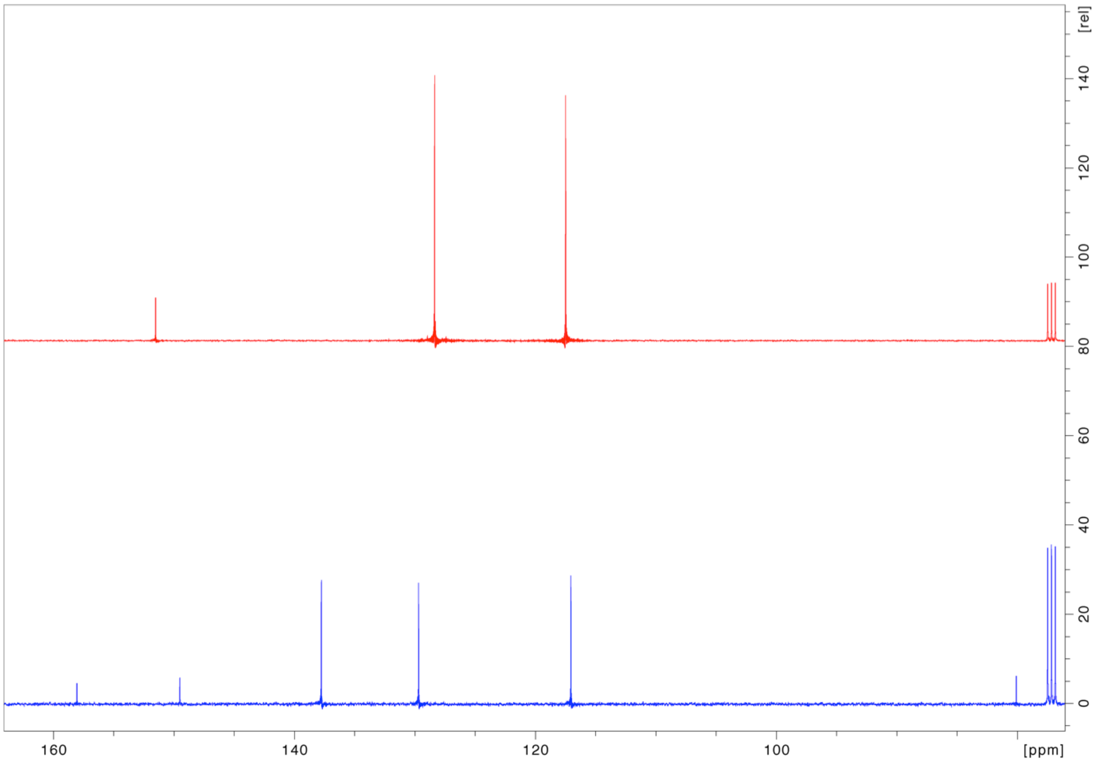

fig. S74. **$^{13}C\{^{1}H\}$ NMR (100.6 MHz; $CDCl_3$, 300 K) spectra of biphenylene (top; red) and 15 (bottom; blue).**

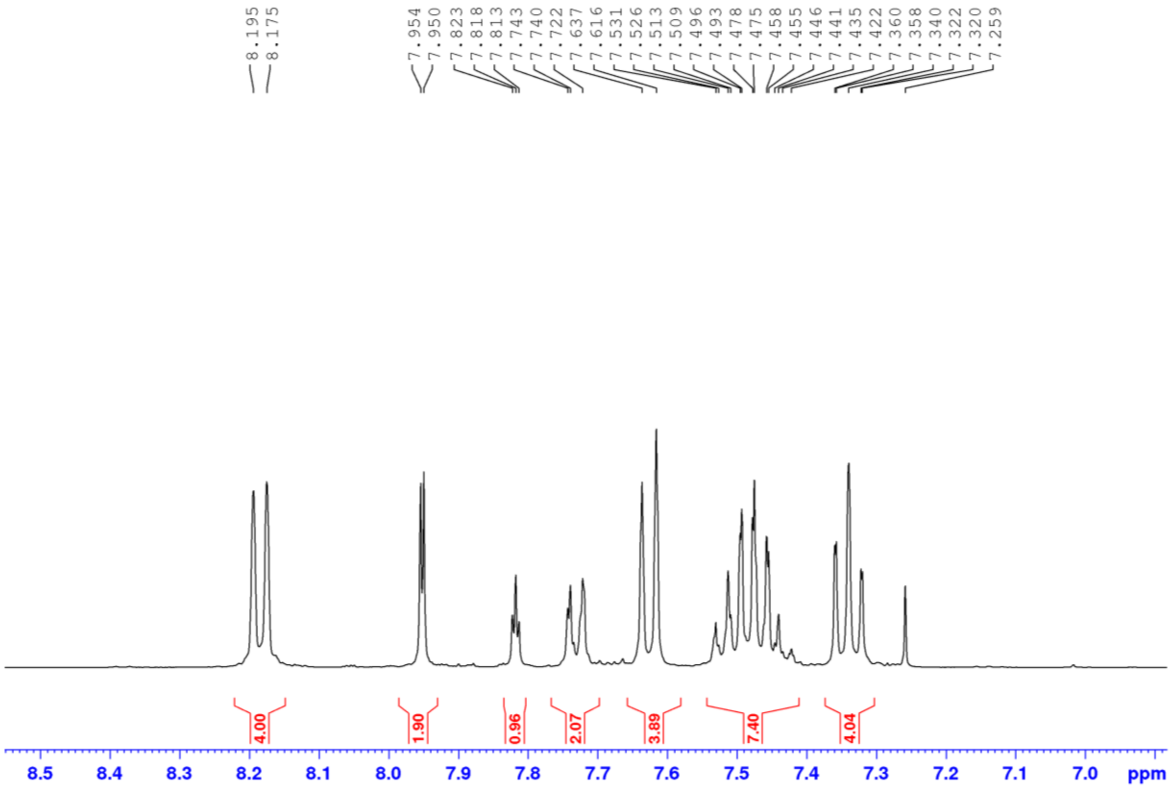

fig. S75. ^{1}H NMR (400.1 MHz; $CDCl_3$, 300 K) spectrum of 16.

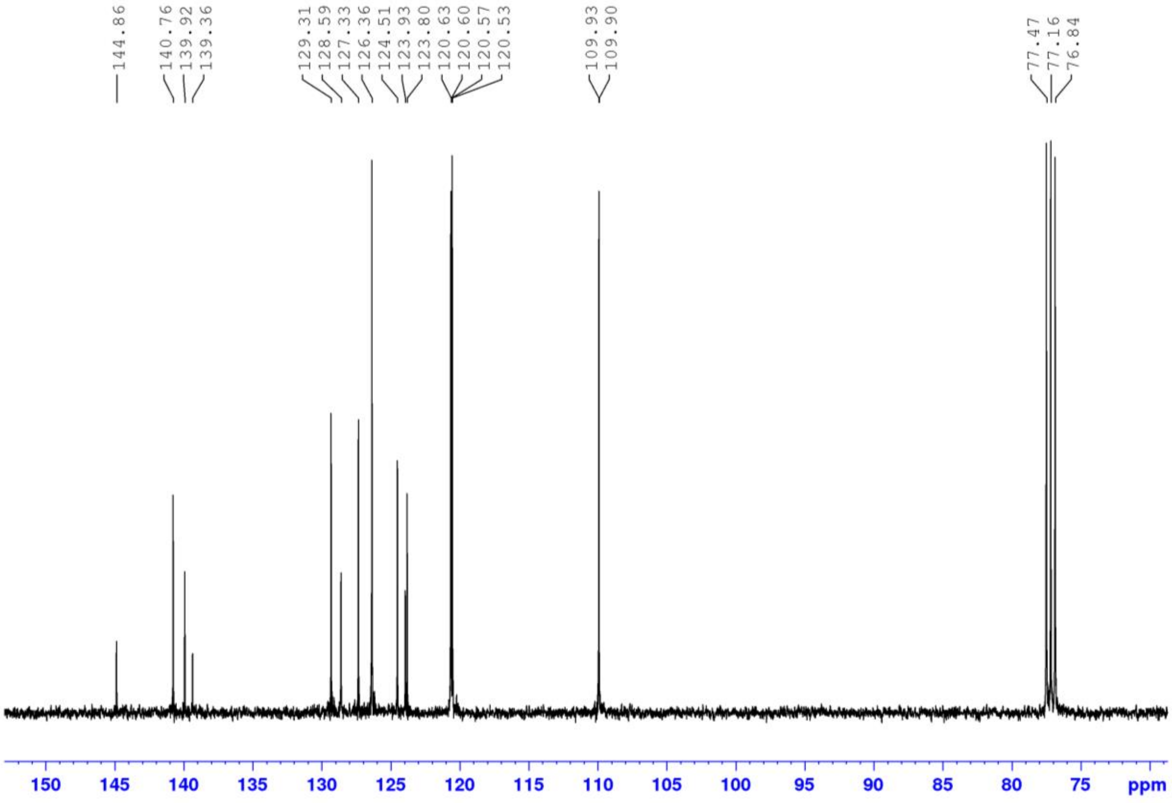

fig. S76. $^{13}C\{^{1}H\}$ **NMR (100.6 MHz; $CDCl_3$, 300 K) spectrum of 16.**

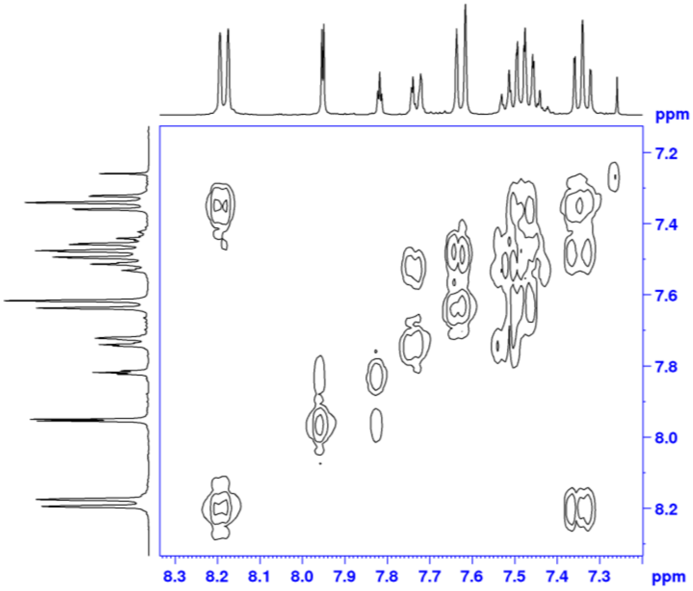

fig. S77. $^{1}H,^{1}H$**-COSY NMR (400.1 MHz; $CDCl_3$, 300 K) spectrum of 16.**

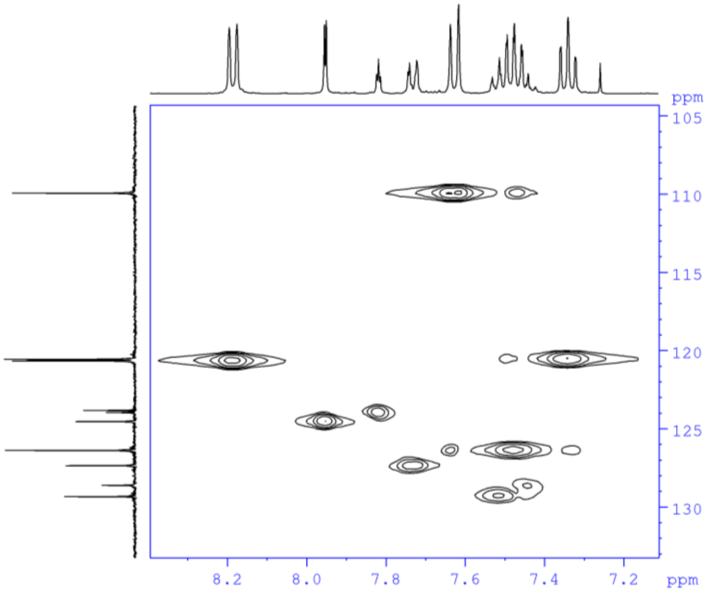

fig. S78. $^{1}H,^{13}C$**-HSQC NMR (400.1 MHz; $CDCl_3$, 300 K) spectrum of 16.**

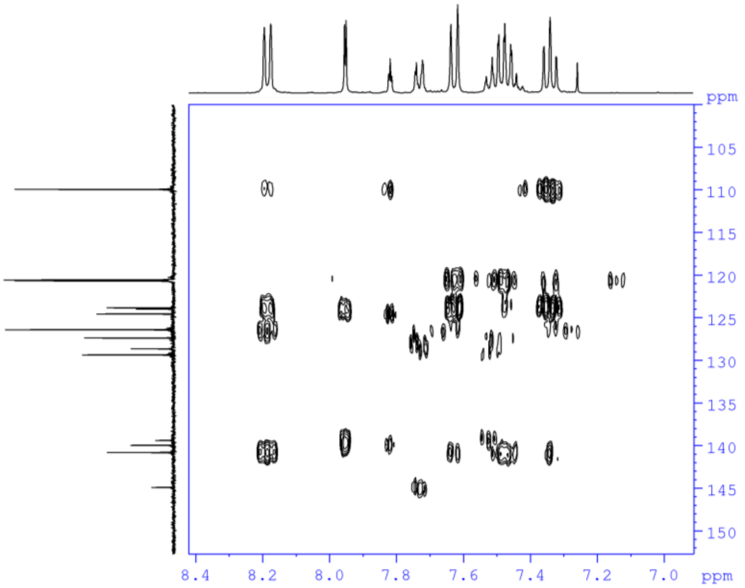

fig. S79. $^{1}H,^{13}C$-HMBC NMR (400.1 MHz; $CDCl_3$, 300 K) spectrum of 16.
